# Dominance and food availability alter avian space use at army ant swarms

**DOI:** 10.1093/beheco/arag111

**Published:** 2026-10-08

**Authors:** Elizabeth J Howard, Andrés F Ramírez-Mejía, Michael Castaño-Diaz, Mary De Aquino, Henry S Pollock, Jeferson Vizentin-Bugoni, Corey E Tarwater

**Affiliations:** Department of Zoology and Physiology, University of Wyoming, 1000 E University Ave., Laramie, WY 82071, United States; Department of Zoology and Physiology, University of Wyoming, 1000 E University Ave., Laramie, WY 82071, United States; Graduate Program in Animal Biodiversity, Department of Ecology, Zoology and Genetics, Universidade Federal de Pelotas, Rua Gomes Carneiro, 01- Centro, Pelotas, RS 96010-610, Brazil; Department of Zoology and Physiology, University of Wyoming, 1000 E University Ave., Laramie, WY 82071, United States; Department of Zoology and Physiology, University of Wyoming, 1000 E University Ave., Laramie, WY 82071, United States; Southern Plains Land Trust, P.O. Box 1016, Lamar, CO 81052, United States; Graduate Program in Animal Biodiversity, Department of Ecology, Zoology and Genetics, Universidade Federal de Pelotas, Rua Gomes Carneiro, 01- Centro, Pelotas, RS 96010-610, Brazil; Department of Zoology and Physiology, University of Wyoming, 1000 E University Ave., Laramie, WY 82071, United States

**Keywords:** army ant swarm, mixed-species group, space use, dominance hierarchy, profitability, newotropical birds

## Abstract

Interspecific competition influences the behavior of species, often altering space use and the ability to forage optimally, particularly for subdominant species. In mixed-species animal groups, differences in dominance among species and prey resource variation across space may result in small-scale variation in space use. Almost 50 yr ago, (Willis and Oniki 1978. Birds and army ants. Ann Rev Ecol Syst. 9:243–263) proposed that space use of birds that follow army ant swarms is driven by dominance hierarchies and spatial variation in food availability at swarms, though this hypothesis remains untested. To test this paradigm, we investigated impacts of interspecific competition and food availability in space use of ant-following birds in Panamá. We conducted surveys of potential prey and of birds to estimate food availability across space (based on prey biomass) and bird space use. We also conducted behavioral observations to evaluate interspecific dominance. We found that food availability varied across space. Contrary to (Willis and Oniki 1978. Birds and army ants. Ann Rev Ecol Syst. 9:243–263) prediction, the swarm perimeter had 43 to 79% more food available than areas within the swarm. Birds generally tended to use areas of higher food availability, dominance was partially based upon dependency on swarms and body size, and the presence of more dominant species (*Phaenostictus mcleannani* and *Eucometis penicillata*) reduced the subordinate species' (*Gymnopithys bicolor*) use of higher food locations by up to half. Future work should examine more ant-following species. Our findings highlight that while species typically forage in locations with more food, dominant species alter space use of subdominants, potentially resulting in different costs and benefits of these species attending mixed-species animal groups (MSAGs).

## Teaser

We looked at how competition and food availability influenced small-scale variation in space use for groups of birds that follow army ant swarms. We found that the more dominant bird species altered space use for subdominant species, resulting in subdominant species being excluded from the locations with more food. This helps confirm that competition between species can influence behaviors, such as how a given species use space.

## Introduction

Interspecific competition plays an important role in structuring communities and in influencing the behavior of species, such as altering space use and the ability to obtain resources (Hairston et al. 1960; Tilman 1982; Kotler and Brown 1988; Ziv et al. 1993). While optimal foraging theory predicts that species should forage in locations where energy intake is maximized (Acevedo et al. 2006; Curras et al. 2021; Brivio et al. 2022), competition may affect the ability of individuals or species to forage optimally. In particular, displacement or chasing between 2 species (defined here as competitive interactions) will alter space use and access to food resources, particularly of subordinates (Vanak et al. 2013; Curras et al. 2021; Basham et al. 2022; Brivio et al. 2022).

Mixed-species animal groups (MSAG), such as mixed-species flocks or herds, are composed of multiple species at the same trophic level that forage together. MSAGs provide a useful system for testing how food and competition alter space use. Dominance hierarchies in MSAGs, combined with spatial variation in food availability, may result in variable space use for species (Carey and Peeler 1995; Jablonski and Lee 2002; Karanth et al. 2017; Chen et al. 2022; Coppinger et al. 2023; Vander Meiden et al. 2024). For example, in mixed-species bird flocks, the presence of dominant species has been shown to result in subdominant species foraging lower in trees or foraging in more marginal habitats, compared with when dominant species are rare or absent (Jablonski and Lee 2002; Vanak et al. 2013). Although some studies have demonstrated that dominant species occupy the best locations and impact the spatial distribution of other species (Jablonski and Lee 2002; Vanak et al. 2013; Curras et al. 2021), studies have rarely addressed how space use is impacted by the interplay between dominance and food availability. Further, studies often do not directly assess dominance based on observations of competitive interactions, and instead body mass is often used as a proxy for dominance (Murton et al. 1971). Here, we use one type of MSAG, army-ant-following birds, to directly evaluate competitive interactions and to understand how food availability and dominance change how birds use space at ant swarms.

Throughout the Neotropics, the predatory army ant species *Eciton burchellii* creates mass swarm raids (hereafter, “swarms”) where they consume and flush prey primarily on the forest floor (Willis and Oniki 1978; Otis et al. 1986). These swarms are akin to slow moving food trucks, and many taxa, most notably birds, take advantage of the arthropods and small vertebrates being flushed by the ants. This results in groups of up to 60 individuals and 20 bird species at a single swarm (Willis and Oniki 1978; Chaves-Campos 2005). Further, more than 465 bird species have been documented attending swarms in the Neotropics alone (Martínez et al. 2021). Within these MSAGs, bird species vary in their dependence on swarms, often defined by how much time they spend at ant swarms, and dominance, defined as their ability to outcompete others. Bird species have been historically classified into 3 categories (obligate, facultative, occasional) based upon these behaviors (Willis and Oniki 1978; Martínez et al. 2018). Obligate species are predicted to be the most dependent upon swarms and the most dominant guild, often being the largest species at swarms and showing aggression to other species (Willis and Oniki 1978; Martínez et al. 2018). Facultative species use swarms on a regular basis, but they are not as dominant or dependent upon swarms as obligates (Willis and Oniki 1978; Swartz 2001; Martínez et al. 2018). Occasional species are the least dependent and dominant, only attending swarms when they pass through their territories (Willis and Oniki 1978; Swartz 2001; Martínez et al. 2018).

The hypothesis for how ant-following birds partition food resources at swarms is based on a dominance hierarchy and food availability diagram proposed in an early, influential review of ant-following birds from Panamá (Willis and Oniki 1978; Fig. 1a is their hypothetical scenario of space use). Willis and Oniki (1978) proposed that larger, more obligate species were behaviorally dominant and obtained the best foraging locations at the swarm, excluding subordinate species to the areas of lower food availability. Despite little to no empirical evidence being available to support or refute these ideas, this paradigm has become broadly accepted in the literature (Martínez et al. 2021). In the Willis and Oniki (1978) diagram, they argue that only birds with similar foraging behaviors (eg, clingers, ground, perchers, and climbers) would influence each other's space use. Here, we work in Central Panama and focus on the same 3 obligate and facultative clingers they show in Fig. 1a (*Phaenostictus mcleannani*, a dominant obligate; *Gymnopithys bicolor*, a subdominant obligate; and *Hylophylax naevioides*, a subdominant facultative). We tested a new dominance hierarchy by adding 1 additional species (*Eucometis penicillata*) that was observed foraging in the same location as the other species, interacting with the other species frequently, and was the third most common species observed at swarms (De Aquino, unpublished data). While *Eucometis penicillata* is defined as a percher (and thus was predicted to interact infrequently with clingers) by Willis and Oniki (1978); Willis (1985) found in a separate study that they were subdominant to *Gymnopithys bicolor* in an area where *Phaenostictus mcleannani* did not exist. In our location of study, we have all 4 species and wanted to understand the role of *Eucometis penicillata* in the dominance hierarchy.

**Figure 1:**
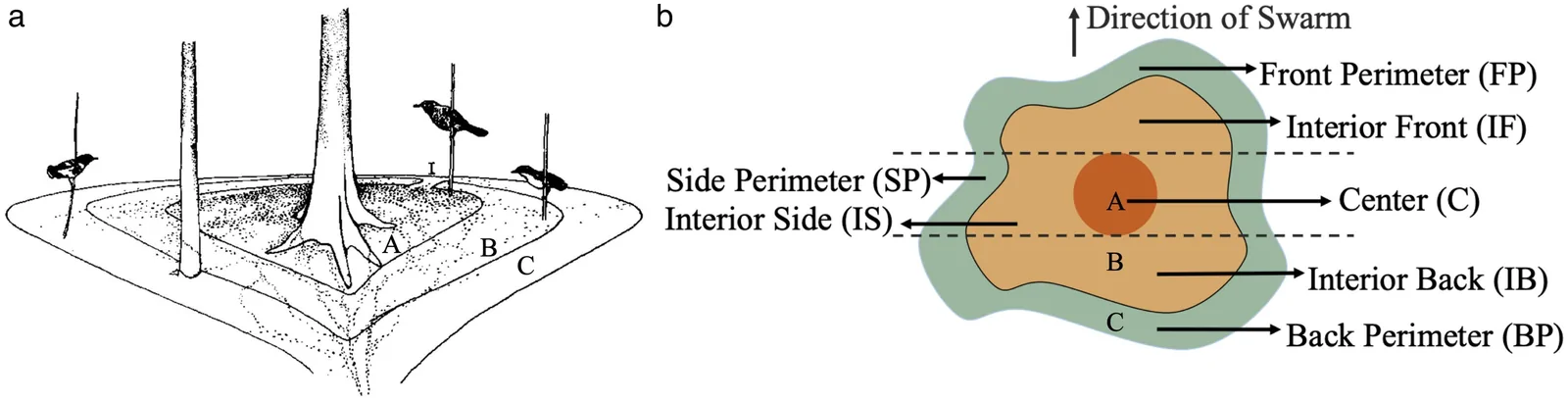
Diagram of an *E. burchellii* ant swarm a) according to Willis and Oniki (1978) and b) our swarm section diagram, with the following areas—FP, IF, center (C), interior back (IB), BP, SP, and IS. In 1a, A represents the center of the swarm, B represents the edge of the swarm, and C represents the area surrounding the swarm. These sections by Willis and Oniki are depicted on our diagram as a comparison, with A representing the center of the swarm (C), B representing the interior of the swarm (IF, IS, IB), and C representing the perimeter of the swarm (FP, SP, BP). In ***a***, the swarm direction is the same as it is in ***b***. 1a is directly taken from Willis and Oniki (1978).

The hypotheses of Willis and Oniki (1978) assume that (i) spatial variation in resources should be partitioned among attendant species based on their dominance (Fig. 1a) and (ii) that food availability (ie, the amount of prey biomass available at a given location) should vary spatially. Here, we tested these key assumptions by making the following predictions based on their hypotheses: (i) a dominance hierarchy exists within prominent ant-following birds in Central mainland Panamá, with more obligate and larger-bodied species winning more competitive interactions compared with nonobligate and smaller-bodies species, (ii) food availability will vary spatially across a swarm, with the highest prey biomass found in the center, then decreasing to the periphery, (iii) ant-following birds tend to use the areas of higher food availability at swarms, and (iv) dominant species will displace subdominant species (in this case, *Gymnopithys bicolor*) to areas of lower food availability. Our study provides a novel test of how food availability and dominance alter space use in complex, MSAG.

## Materials and methods

### Study sites and focal species

The study was conducted from February to July of 2023 in Parque Nacional Soberanía, a 22,000 ha lowland tropical moist forest in Central Panamá. Study sites included locations along Pipeline Road (9°9′35″N, 79°44′36″W) and Plantation Road (9°4′35″N, 79°39′32″W). Climate is marked by a dry season from January to March, and a wet season from April to December (Windsor 1990). Pipeline Road has an average yearly rainfall of 2,331 mm, while Plantation Road has an average yearly rainfall of 2,167 mm (data derived from the Smithsonian Tropical Research Institute's Physical Monitoring Program). Forests are primarily mature secondary forest, with some patches of old-growth forest.

In Panamá, there are 2 main swarm-raiding army-ant species whose swarms ant-following birds attend (*Eciton burchellii* and *Labidus praedator*), but *E. burchellii* is the only species that forms above-ground living nests called bivouacs, making them easier for a suite of other taxa to detect and follow (Willis and Oniki 1978; Rettenmeyer et al. 2011; Gómez-Murillo and Tarwater 2026). *E. burchellii* go through nomadic and statary (ie, stationary) phases that align with their life cycle (Rettenmeyer et al. 2011). During the 20-day statary phase, the ants keep the bivouac in the same location, they may produce swarms that birds follow, or they may be completely dormant (Rettenmeyer et al. 2011). After the statary phase, there is a 15-day nomadic phase during which time, the colony swarms almost daily and moves the bivouac almost every night (Schneirla 1940; Schneirla 1971; Rettenmeyer et al. 2011).

During raids, *E. burchellii* move across the forest floor to flush up any possible prey, most notably larvae, pupae, and arthropods they come across, which attracts many different taxa to join these swarms (Schneirla 1971; Rettenmeyer et al. 2011). Ant-following birds are some of the most notable attendees at these swarms. We have documented nearly 70 species attending swarms at our study sites (Table S3). Here, we focus on the 3 species included in Willis and Oniki (1978; Fig. 1a) and 1 additional species to test the purported dominance hierarchy and space use among bird species foraging near the ground that follow swarms regularly: *Phaenostictus mcleannani* (Ocellated Antbirds, the dominant obligate species, average mass of 48.76 g; Wilcox et al. 2024), *Gymnopithys bicolor* (Bicolored Antbirds, the subdominant obligate species, average mass of 29.63 g; Wilcox et al. 2024), *Hylophylax naevioides* (Spotted Antbirds, the least dominant facultative species, average mass of 16.08 g; Wilcox et al. 2024), and the new species not discussed in Willis and Oniki (1978), *Eucometis penicillata* (gray-headed Tanager, a facultative species, 30.41 g, Wilcox et al. 2024).

### Locating and tracking ant swarms

We located army ant colonies by walking rectangular transects (500 m long × 200 m wide) from an initial starting point. We located colonies by looking for ants moving in lines or raids and listening for vocalizations from birds who actively follow swarms. Once we located the ants, we tracked them back to the bivouac, which we marked with a GPS device and flagging. We tracked bivouacs twice a day, once during the day for focal behavioral observations and once at night to find their new bivouac locations. We rotated between Pipeline Road and Plantation Road approximately every 10 days, and during each 10-day period, we followed the same swarm for up to 3 days in a row and searched for more swarms as needed.

### Dominance hierarchy among ant-following bird species

We used behavioral observations to determine pairwise dominance interactions among the 4 species of ant-following birds. Here we define a competitive interaction as occurring when there is displacement or chasing between 2 species. We base the hierarchy on win-loss records between the pair of species, as was done by Willis (1973); Willis and Oniki (1978) and is commonly done in other studies of dominance hierarchies (Pratt et al. 2003; Tibbetts et al. 2022). Two observers conducted behavioral observations during 8, 10-min windows. Observations took the form of focal sampling (Altmann 1974), with each observer focusing on one randomly chosen bird for approximately 30 s before changing their attention to another bird. We aimed to perform observations on different individuals of the same species, rather than repeated observations on the same individual. During each 30-s focal period, observers recorded the following information on competitive interactions: the species involved, the species that initiated the interaction, the identities of the winner and loser, and the interaction type (displacement *versus* chasing). Displacements involved 1 bird physically displacing another, causing the displaced individual to fly away and leave their original location, whereas chasing involved active pursuit of 1 individual by another. Here we are not interested in what is changing the amount of competition at swarms or even the amount of competition between the different species, but rather who wins in an aggressive encounter (following Willis 1973).

### Space use of birds and food availability at swarms

We conducted observations of swarms daily between 07:00 and 14:00 to evaluate spatial variation in attendant birds and their potential prey. Only one observer collected all space use data, ensuring no potential impacts of interobserver variation. We conducted observations only at colonies that were actively raiding. The front of the swarm, in other words, the direction in which the ants were headed, was considered the leading edge of the swarm. The back of the swarm was considered the place in which the ants transitioned from a large mass to lines. Swarm shape was estimated by walking the perimeter of the swarm and drawing the swarm to scale on a gridded piece of paper, with each cell representing 1 m^2^ (Figure S1). We verified the accuracy of the drawings using rangefinders and by taking multiple GPS points around the swarm to estimate swarm area.

We conducted multiple rounds of point counts and potential prey surveys for each swarm on a given day. In each round, first we estimated the swarm area and then we waited 5 min before collecting bird data in case we disturbed any birds when estimating the area. To facilitate observation, each swarm was broken up into sections based on areas that were visible to the observer to minimize interference from obstructive vegetation. We then conducted a 1-min point count of bird species in each section (or the entire swarm, depending upon size). The number of point counts conducted was based on the number of sections in a swarm and ranged from 1 to 3 point counts (average = 1.72, SE = 0.0732). Bird species, number of individuals, and location were written down on the gridded paper. We observed all birds within the swarm and up to 15 m outside the swarm edge (following Jullien and Thiollay 1998; Martínez et al. 2018). We only included birds that were 2 meters or less off the ground because these are typically the birds foraging on the ground (which is the area we surveyed for potential prey). We did not include the birds foraging higher in the trees because we could not easily conduct potential prey surveys up in the trees and these species (eg, the woodcreepers) likely have a separate dominance hierarchy owing to their different foraging locations (Willis and Oniki 1978). We rarely observed those species foraging in higher locations interacting with the species lower to the ground (De Aquino, unpublished data). The same individual may have been observed multiple times within the 1-min observation window and we recorded all locations that a bird was seen, irrespective of whether it was the same or a different individual. After point counts were conducted in all sections, we conducted potential prey surveys (see below). Round one of data collection concluded when prey surveys were complete. We would then wait 5 min before we drew out the swarm area again and began another round of point counts and prey surveys. We completed between 1 and 8 rounds (average = 2.63, SE = 0.166) of data collection at 1 swarm per day, depending upon weather, time of day, and raiding behaviors.

#### Space use: prey surveys

We surveyed potential prey using 2 methods—line transect surveys and quadrat surveys—to understand spatial variation in food availability (Figure S1). We used line transect surveys to obtain information on prey that were stationary (one of the strategies prey employ to avoid predation) and prey that were captured by ants (resulting in clumps of ants attacking prey) (Wrege et al. 2005). We used quadrat surveys to obtain information on prey that were escaping the swarm (the prey on the perimeter of the swarm), including prey that ran or flew away (another strategy commonly employed by prey; Touchton and Smith 2011). Overall, both survey methods aimed to gather information on where potential prey items were found both within and on the perimeter of a swarm (defined as within 5 m of the swarm edge). We also conducted transect and quadrat surveys on potential prey outside the swarm (>15 m from the swarm edge) to evaluate the difference of food availability within swarms versus away from swarms (Martínez et al. 2018). We verified that our potential prey surveys were an adequate representation of the types of prey consumed by our bird species using our behavioral observation data (see Table S2 for details). For details on how prey biomass was calculated, see Table S4 and Section S4.

#### Transects

We spaced transects every 2 meters, walked the entire length of the swarm until 2 meters past the front of the swarm, and in the direction that the ants were moving. The number of transects within the swarm depended upon the width of the swarm, ranging from 1 to 8 transects (average = 3.24, SE = 0.159) within the swarm. We walked transects slowly at a rate of ∼30 s per meter and looked for potential prey or prey being consumed within 1 m of either side of the transect line. We took data on the size of live prey or the size of a clump of ants (indicating a prey being consumed), location within the swarm based on the gridded paper, and prey order, if it was detectable (Wrege et al. 2005). We conducted 1 to 2 transects outside the swarm. These transects were roughly parallel to the ones conducted inside the swarm. For smaller swarms where only one inside transect was conducted (swarms <4 meters in width), only one outside transect was conducted. For larger swarms, 2 outside transects were conducted. Outside transects were conducted during each round.

#### Quadrats

We used 0.5 m^2^ quadrats laid out along the edge of the entire swarm every 5 meters. The number of quadrats varied based on the size of the swarm, ranging from 1 to 8 quadrats (average = 4.15, SE = 0.15). The observer watched the quadrat for 1 min to observe any potential prey within or going through the quadrat. We disturbed the leaf litter within the quadrat during observations to identify any prey hiding underneath. We took the size and order of the potential prey, if detectable. For each quadrat along the edge of the swarm, there was an equivalent quadrat that was evaluated outside of the swarm, from 1 to 8 quadrats (average = 4.15, SE = 0.150). Each outside quadrat was placed at the same angle as its equivalent edge quadrat. We conducted 1 to 8 outside quadrats during each round (Figure S1).

### Analyses

To examine spatial variation of food availability and space use by bird species, we split the swarm into 7 different spatially explicit areas (Fig. 1b). The center (C) is composed of the middle 25% of the swarm. The interior front (IF) and back (IB) refer to the front and back 25% of the swarm, respectively. The interior side (IS) refers to 12.5% of the swarm area on either side of the center, not within the IF or back. All perimeters, front perimeter (FP), side perimeter (SP), and back perimeter (BP), are the outside of the swarm, up to 5 m, outside their associated interior location. Outside the swarm is defined as greater than 15 m away from the edge of the swarm.

We used R 4.2.1 programming language (R Core Team 2022) to analyze the data and conduct simulations. See Material S3 for all packages used.

#### Hypothesis 1: dominance hierarchy

We calculated the percentage of wins between 2 species based on the number of wins out of the total number of pairwise interactions. This was done for all pairwise combinations across the 4 species examined.

#### Hypothesis 2: spatial variation in food availability

We used hierarchical Bayesian models to analyze the variation of prey biomass within (using the different sections described above) and outside the swarm (as a control). To do so, we modeled prey biomass per quadrat –our sampling unit– as a function of its location at the swarm, swarm identity, sampling round, and date. We included an offset term to account for sampling effort variation among quadrats (See information S4.2). A log-normal distribution was defined as the likelihood function and partial pooling was used to estimate the effect of grouping factors. To account for the potential temporal correlation across sampling dates, we estimated the date parameter using Gaussian processes and programed a quadratic kernel to construct the covariance matrix of an *MVnormal* distribution to estimate correlated parameters (McElreath 2020) (material S4.2). We used skeptical priors of strong effects, parameterized to encompass values within the possible range of prey biomass. To set up the Hamiltonian MCMC algorithm, we defined 3 chains, 2,000 sampling and 500 warming iterations, and a thinning rate of 3 (material S4.4). Finally, we conducted sampling diagnostics of the model through visual assessment of chain convergence, *Rhat* < 1.05 and *ess* > 100 per chain (material S4.5). The quality of the models' fit was assessed through posterior predictive checks (material S4.6). See material S4 for a detailed mathematical description of the models, prior probabilities, and Stan code.

#### Hypothesis 3: spatial variation in ant-follower presence at swarms and relationship to prey

We first examined spatial variation in ant-follower presence across all 4 bird species (material S6.2 and S6.7). This model combined all observations of all ant followers to assess how ant-followers use space. Swarm area was included since it impacts the number of ant-followers and prey biomass (Gómez-Murillo et al. 2026). This model encompasses a set of linear equations denoting the causal structure among variables (eg *swarm area* → *prey biomass*, *swarm area* → *ant-followers*, and *swarm area* → *prey biomass* → *ant-follower presence*). We used a Student-T distribution as the likelihood function to model the *swarm area* and *prey biomass*. Those variables were modeled as a function of swarm identity, sampling round, site, and date; however, the prey biomass model also included the effect of swarm area stratified by the swarm section and an offset term to account for variation in sampling effort (material S6.2). We used a Bernoulli distribution to model the presence of ant followers, including the swarm section as predictors, sampling round, site, date, swarm area, and prey biomass (material S6.2). We used skeptical priors of strong effects for all models, partial pooling to estimate grouping factors, and Gaussian processes to account for temporal correlation among sampling dates. We set the HMC algorithm, conducted sampling diagnostics, and assessed the models' fit as described above. See material S5–S7 for a detailed mathematical description of the models, prior probabilities, and Stan code.

#### Hypothesis 4: influence of dominant species on subdominant space use

We evaluated patterns of space use at the species level, as done in Willis and Oniki (1978), to evaluate how dominance influenced space use (material S6.2 and S6.7). This model follows the same structure as the model from above regarding the likelihood, grouping predictors, and Gaussian processes, but also accounts for the effect of co-occurrence of the most dominant species *i* at the swarm section *j* (see material S6.2 and S7.2 for a detailed mathematical notation of species and assembly-level model, respectively). After verifying sampling diagnostics and model fits, we used the species-level model to explore the effect of dominance on space use by ant-followers through the simulation of 2 types of interventions: (i) probability of observing a *subordinate species i* alone at the *swarm section j*, and (ii) probability of observing a *subordinate species i* when *N dominant species* co-occur at the *swarm section j*. To conduct the interventions, we conditioned the effect of swarm area and prey biomass on average values. We focused on *G. bicolor* since we had little data for *P. mcleannani*, *E. penicillata* and *H. naevioides*, and the coefficients were highly variable (material S27.7.3.1, S7.7.3.2 and S7.7.3.4). See material S7.7.3 for the R code to conduct simulations.

## Results

We studied 38 different swarms from February to July 2023 (details on number of swarms and observations per month are found in Table S1). We took data on competitive interactions for 270 h across the 38 swarms. We collected data on 14 swarms from May to July 2023 (during the wet season only) to understand space use and food availability, for a total of 96 observational rounds (details on number of swarms and observations per month are found in Table S1).

### Dominance hierarchy among species

We found that the large obligate antbird, *P. mcleannani*, won most pairwise interactions by chasing or displacing all other species (Fig. 2). Interestingly, the tanager *E. penicillata*, was the second most dominant species at swarms, winning nearly 90% of interactions with the other 2 species (*G. bicolor* and *H. naevioides*). The subordinate obligate, *G. bicolor,* typically won against the facultative *H. naevioides* (91.9% of the time), but rarely won against the other 2 species. Finally, *H. naevioides* was the least dominant of the 4 species, only winning 8.3% of the time (4 out of 48 interactions).

**Figure 2:**
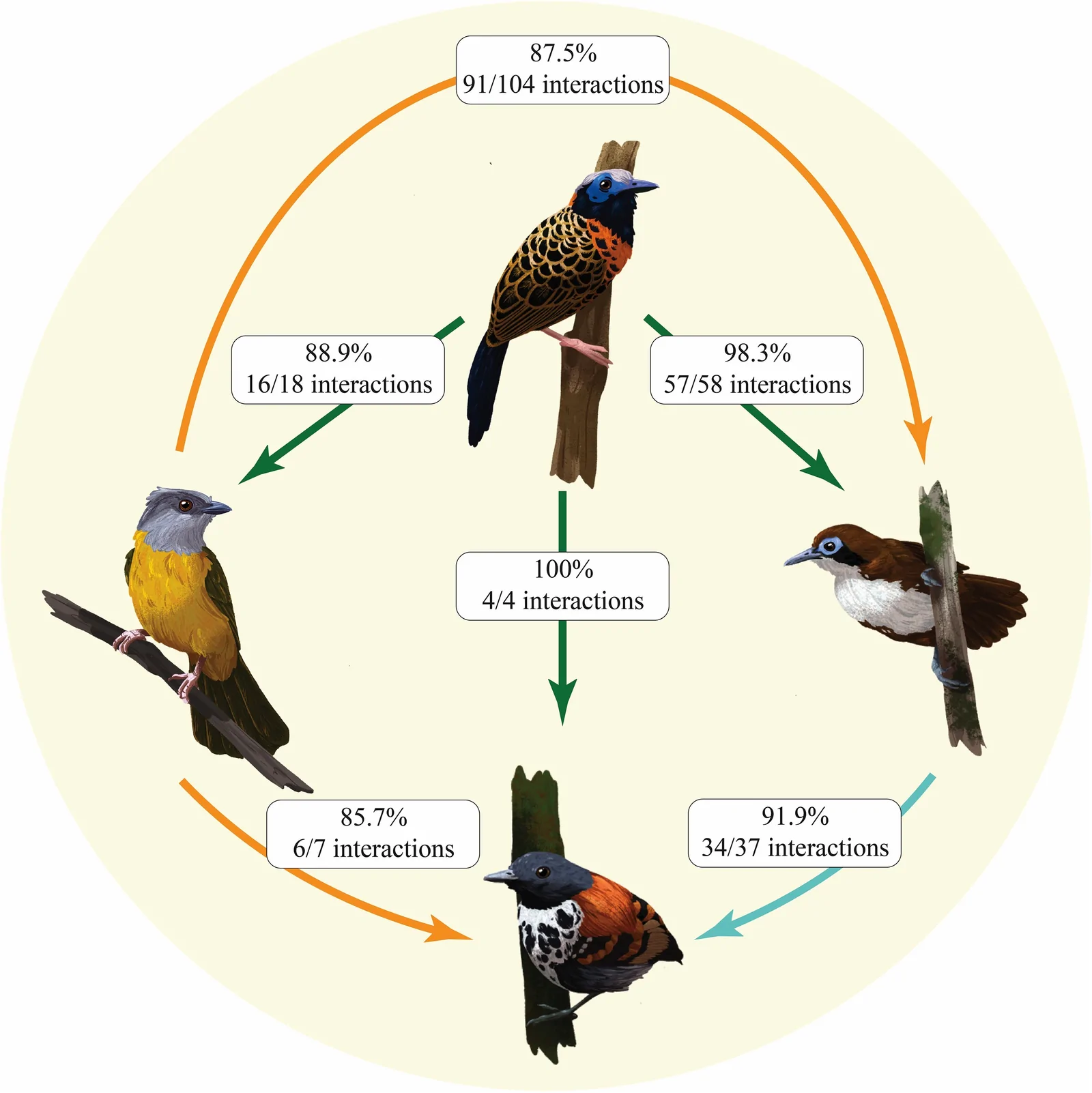
A diagram displaying outcomes of pairwise dominance interactions among the 4 focal species of ant-following birds observed attending ant swarms at the study sites in Central Panamá. Direction of arrows indicates which species displaced/chased the other, with percentage of interactions won between each focal species, *Phaenostictus mcleannani* (top), *Gymnopithys bicolor* (right), and *Hylophylax naevioides* (bottom), and *Eucometis penicillata* (left) listed for each pairwise interaction. Drawings done by Jose Riascos.

### Spatial variation in food availability

We found that food availability was highest at the perimeters of swarms compared with the center or interior (Fig. 3a, all details on probabilities are in material S4.7), in contrast to predictions. The FP of the swarm had the highest prey biomass compared with any other section. The FP had 79% more prey biomass compared with any section inside the swarm and 77% more prey biomass compared with outside the swarm. The FP also had higher biomass than the BP (25% higher) and the SP (17% higher). The SP had 54% more prey biomass compared with any section within the swarm (other than the back and front perimeters) and 52% more biomass compared with outside the swarm. Finally, the BP had 43% more prey biomass compared with any section within the swarm (other than the side and front perimeters) and 42% more biomass compared with outside the swarm. The prey biomass at the SP and BP tended to be similar, with the SP being only slightly higher (8% higher). The different sections inside the swarm did not differ in prey biomass, nor did they differ from outside the swarm (material S4.7).

**Figure 3:**
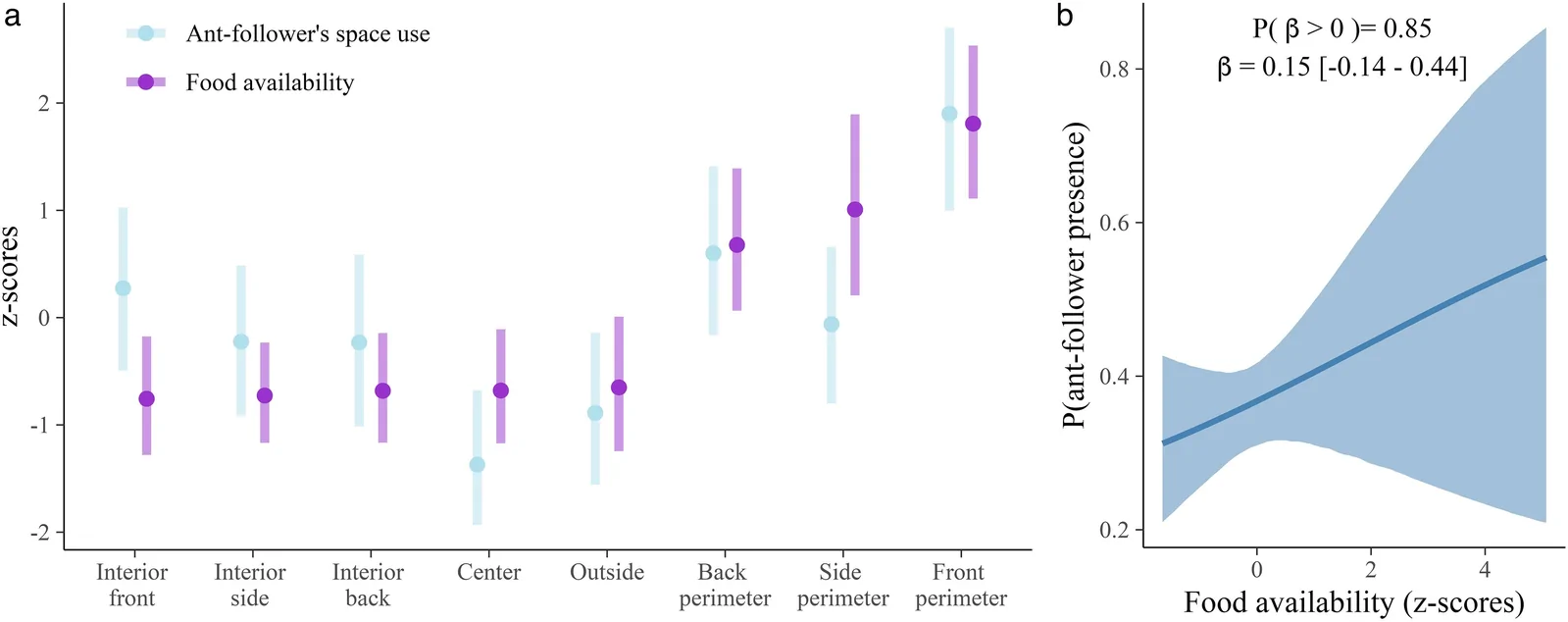
Food availability (ie, prey biomass) and space use (ie, probability of species' presence) of ant-follower birds across different areas within and outside the swarm, and conditional effect of food availability on ant-follower space use. a) Dots and bars indicate average and 95% credibility intervals of the posterior distributions. Values are *z*-scores scaled for visualization purposes and areas are ordered from lowest to highest food availability. b) Trend line and shaded region indicate average effect and the 95% credibility interval.

### Spatial variation in ant-follower presence at swarms and relationship to prey

We found that the probability of ant-follower presence (across the 4 bird species) increased with prey biomass (Fig. 3b), meaning the birds tended to use sections of the swarm with higher food availability (Fig. 3a, material S4.7 and S6.6.4). In particular, the FP had the highest probability of presence of ant-followers (65%), followed by the BP (46%), and the IF (42%, Fig. 3a). The swarm section with the lowest probability of being used by ant followers was the center (25%), which was the same as the outside of the swarm (18%, Fig. 3a). Correspondingly, the FP had the highest prey biomass and the center had low prey biomass (Fig. 3a). Consistency between the probability of ant-followers and food availability occurred in all sections of the swarm, except for 2—the IF and SP (Fig. 3). The IF section of the swarm had relatively low prey biomass, yet had the third-highest probability of observing ant-followers (Fig. 3a). The SP section had the second-highest prey biomass, yet the fourth-highest probability of observing ant-followers (Fig. 3a).

### Influence of dominant species on subdominant space use

We were only able to evaluate if 1 species, *G. bicolor,* altered its space use based on the presence of dominant species (*E. penicillata*, *P. mcleannani*), owing to the scarcity of data for the other species. The effects of more dominant species on *G. bicolor* were mainly detected on the FP of the swarm: the section with the highest food availability and highest probability of *G. bicolor* presence (Fig. 4). In particular, when *G. bicolor* was alone in the FP of the swarm, the probability of using this section was 44% (95% CI: 0.31 to 0.57). However, when either species (*E. penicillata* or *P. mcleannani*) dominant to *G. bicolor* co-occurred with *G. bicolor* in the same area, the probability of *G. bicolor* using the FP dropped to 26% (0.11 to 0.45) and 35% (0.16 to 0.57), respectively (Fig. 4). Notably, the decrease in probability was even more pronounced when both dominant species were present (dropping to 20%, 95% CI: 0.06 to 0.43, Fig. 4, material S7.7.3.3). Thus, *G. bicolor* shift their space use (to an area with lower food availability) when alone versus when near a dominant species.

**Figure 4:**
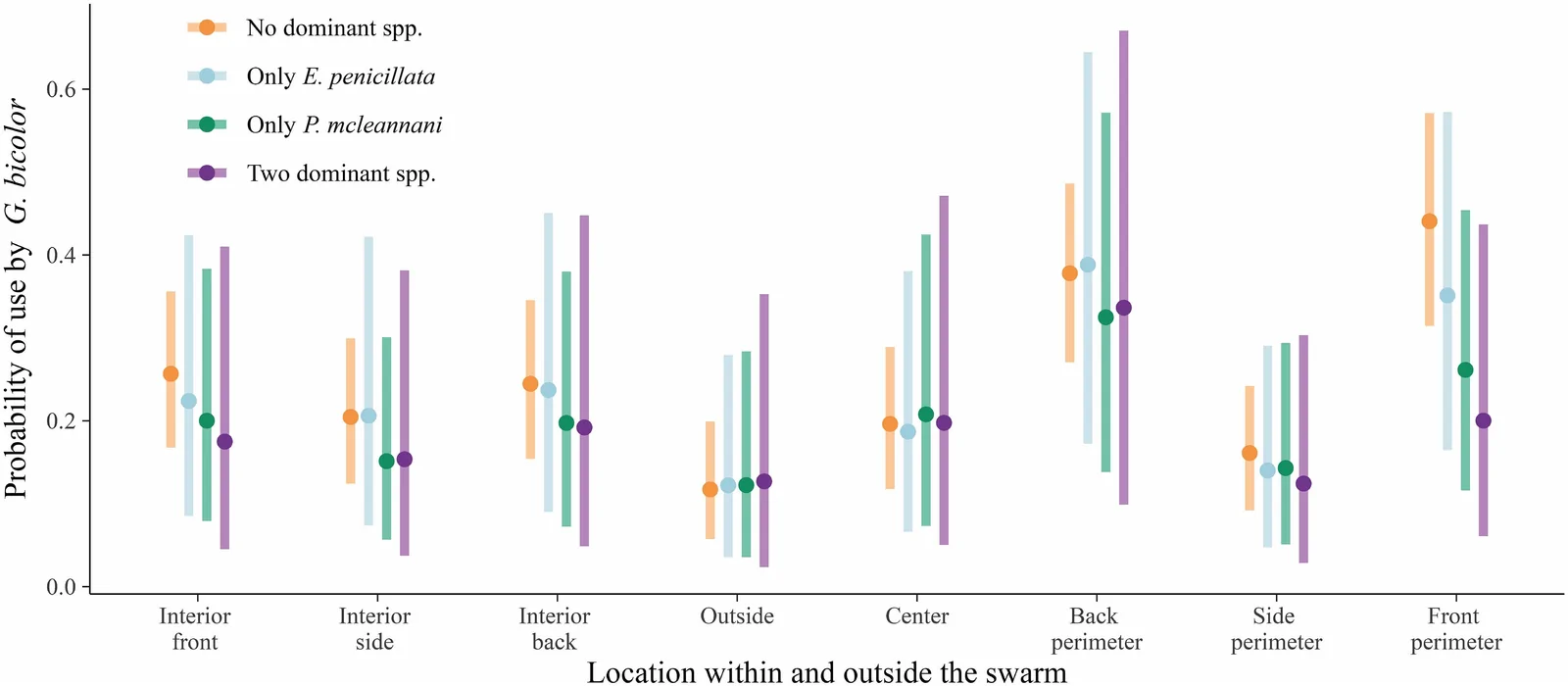
Probability of space use by the subordinate obligate ant-follower *G. bicolor* at different areas within and outside the swarm under different scenarios of potential competition (only *E. penicillata*, only *P. mcleannani*, or both dominant species). Areas are ordered from lowest to highest food availability.

## Discussion

Overall, our results support the role that dominance plays in altering space use and foraging in a mixed-species animal group (MSAG), highlighting the importance of interspecific competition in communities. Specifically, we found the following: (i) as predicted, food availability varied across space, and birds tended to use these areas of higher food availability; (ii) dominance was partially based upon dependency and body size, and (iii) dominance influenced space use, with the dominant species altering the use of the more profitable locations by the subdominant species, *G. bicolor*. Our results ran counter to some of the assumptions of Willis and Oniki (1978). In particular, we found the most profitable locations are on the perimeter of the swarm and most bird species are on the perimeter, rather than in the center or interior as predicted by Willis and Oniki (and as depicted in Fig. 1a). Further, we found that *E. penicillata* are dominant over *G. bicolor*, in contrast to previous predictions (Willis and Oniki 1978; Willis 1985). While further work is needed across a larger suite of ant-following birds, here, we provide one of the first quantitative studies evaluating the role of interspecific competition and food availability in structuring space use at army ant swarms.

### Dominance hierarchies among ant-following bird species

Interspecific dominance hierarchies often arise between species competing for similar resources and are predicted to favor behaviors that reduce interspecific aggression and minimize competition (Coppinger et al. 2023). Dominance is often linked to body size across species, with larger species having an advantage in aggressive contests and being dominant to smaller species (Murton et al. 1971). In MSAG, the role of species may also influence dominance; typically, species with critical roles to the formation and/or maintenance of groups (eg, nuclear species) are dominant over occasional species (Polis et al. 1989; Coppinger et al. 2023). Based on Willis and Oniki (1978), we predicted that dominance would be based on body size and dependency on the swarm, with larger-bodied, more obligate species being dominant to smaller-bodied, more facultative species. Our results were largely consistent with these predictions. The largest species and an obligate species, *P. mcleannani* was clearly dominant to all other species. Conversely, the smallest species and a facultative species, *H. naevioides*, was subordinate to all other species.

In contrast to the predictions for dependency class, however, *E. penicillata* was dominant to *G. bicolor*, even though *E. penicillata* is considered a facultative species. There are multiple potential explanations for these findings. First, we may be incorrectly defining *E. penicillata* as facultative. How dependency class is defined is not clear and roles may be context-dependent or vary along a continuum. For example, dependency has been defined based on different metrics including species occurrence at swarms, the presence of bivouac checking behaviors, the percentage of the species' diet that comes from swarms, or their role in recruiting other species to swarms (Oniki and Willis 1972; Swartz 2001; Pollock et al. 2017; Martínez et al. 2018). Depending on which definition we use, *E. penicillata* may or may not be facultative. Ideally, we would know how much time birds spend foraging outside of swarms, as well as at swarms; however, very few studies have this information, and thus we likely have an incomplete picture of dependency class. Second, dependency may not play a large role in dominance. For example, within antswarms, species tend to compete with those with similar foraging heights and behaviors (ie, use the same substrates). Within the subgroups that tend to compete more, dependency class may play less of a role than other traits about the species, such as their abundance, morphological traits, or other attributes that influence their ability to win fights (Chen et al. 2023). While *E. penicillata* and *G. bicolor* are <1 g different in body mass, *E. penicillata* have longer wings, wider and deeper bills, and longer tails (Tobias et al. 2022; Wilcox et al. 2024). While body mass is the focus of most studies on dominance, other morphological traits that result in an overall larger body size may be more important (Chen et al. 2023). A greater suite of species and traits need to be evaluated to determine the role of dependency versus other traits on dominance at ant swarms. Nevertheless, observing interactions between species when able, rather than assuming dominance based on traits, would be highly beneficial (Chen et al. 2023; Bassing et al. 2024).

Here we show that even though species do engage in competitive interactions with each other, these interactions are rare. For example, here we only observed 228 interactions across these 4 species out of the 270 h of observations at swarms. These results suggest that the dominance hierarchies at ant swarms, at least among regular ant-followers, may be well-defined and consistent, reducing the need to escalate interactions to the point of chasing or displacing. The function of many of the displacements observed here could be for stealing prey, rather than maintaining the dominance hierarchy. Maintenance of dominance hierarchies over longer time periods reduces the need to reinforce them on a frequent basis, saving individuals time, energy, and risk of injury (Tibbetts et al. 2022). Willis (1967, 1973) suggested that dominance is achieved (and perhaps maintained) through a series of aggressive and submissive displays, with chasing being a rare occurrence. We did not evaluate displays, but understanding these more subtle behaviors, in addition to the role of perch type and angle, would be useful for future studies. Additionally, competitive interactions may be rare because high food availability at swarms reduces the need to compete (O’Donnell 2017). For example, previous work in Central Panamá found that dominant species are more common at larger swarms and that larger swarms have more arthropod biomass (Gómez-Murillo and Tarwater 2026; Ramírez-Mejía et al. in review). Thus, competitive interactions may be more frequent at smaller swarms with fewer arthropods. Further work is needed to evaluate these hypotheses.

### Foraging in more profitable locations

One of the main benefits of joining MSAG is increased foraging, and at ant swarms, the flushing of arthropod prey by the army ants is a key benefit to the ant followers (Wrege et al. 2005; Goodale et al. 2020; Dominguez et al. 2023; Chen et al. 2025). Here we found that prey biomass is greater within the swarm (along the perimeters) compared with outside the ant swarm (>15 m away) and that ant-following bird species, in part, use potential prey biomass to determine where to forage within a swarm. Birds may base their foraging location on their own personal information or socially acquired information gathered on the current presence of prey and/or previous experience, as might occur in the species that regularly follow ant swarms (Pyke 1984; Dall et al. 2005). The 2 slight exceptions to birds using the more profitable locations are our findings that the IF had a high probability of bird use despite lower food availability and the SP had a lower probability of bird use despite higher food availability. These 2 inconsistencies may be caused by small-scale variation in predation risk or interspecific competition shifting what locations to use or to avoid. While further studies are needed, our results are congruent with theory predicting that species choose foraging patches with higher food availability (Pyke 1984; Bassing et al. 2024), and that they can do so at small spatial scales.

### Dominance of obligate species alters space use of subdominant species

One of the primary hypotheses invoked to explain how ecologically similar species may coexist is through resource partitioning, with one mechanism of partitioning being spatial segregation of species (MacArthur 1958; Karanth et al. 2017; Kent and Sherry 2020; Miller et al. 2025). Often, spatial segregation arises via the subdominant species avoiding the areas with dominant species or being chased by the dominant species out of those areas; these behaviors result in the dominant species occupying higher quality habitat and subdominants occupying lower quality habitat (Durant 1998; Acevedo et al. 2006; Vanak et al. 2013; Monterroso et al. 2020; Brivio et al. 2022; Basham et al. 2022). Within MSAG, spatial segregation of species may occur at small spatial scales (ie, within a single tree or a branch) and may lead to subdominant species altering their foraging stratum or leaving their current foraging area in the presence of dominant species (Morse 1978; Jablonski and Lee 2002; Chen et al. 2022; Coppinger et al. 2023). Similarly, we found that the more dominant species (*P. mcleannani*, *E. penicillata*) altered the space use of the subdominant species (*G. bicolor*), resulting in the subdominant species being less likely to occupy areas of higher food availability. This reduction in space use was even more pronounced when both dominant species were present, compared with just 1 species. While further work is needed on a greater number of species, our work highlights that interspecific dominance alters space use and may alter the benefits subdominants receive from attending MSAG.

### Reevaluating the Willis and Oniki (1978) paradigm

While our results are in general agreement with the predictions set forth by Willis and Oniki (1978), some initial assumptions were refuted. First, while food availability did vary spatially across swarms as they predicted, the best foraging locations in the swarm were on the perimeter, while the center/interior of the swarm had the lowest food availability. Further, bird species were more likely to be along the perimeter of the swarm, rather than the interior. Previous work on leaf litter arthropods suggests that >50% of organisms (depending on order) successfully escape the army ants, either by fleeing to overhead vegetation, moving out of the path of the ant swarm, or hiding deeper in the leaf litter (Otis et al. 1986). Here, our work supports the idea that arthropods move out of the path of the ants (to the perimeter), where birds are present to take advantage of the escaping nonant arthropods. It is possible that birds may adjust their space use to capture certain types of fleeing prey (depending on what prey are more easily captured by a given bird species), but more work is needed to test this. Second, the role of *E. penicillata* differs from the Willis studies. While the Willis and Oniki (1978) study assumed that *E. penicillata* did not compete with the antbird species examined here owing to differences in perch behavior, a subsequent study found that *E. penicillata* was subdominant to *G. bicolor* (based on work on an island missing *P. mcleannani,* Willis 1985). Our results contrast with both studies. Although there are some differences in where they perch, *E. penicillata* spend time near the forest floor, obtain prey from the leaf litter, and engage in many interactions, second only to *G. bicolor*. We argue that perching behavior may have a weaker influence on which species are competing compared with the substrate that they forage on (eg, leaf litter, bark, etc) and their foraging heights. The presence of *P. mcleannani* may alter competitive interactions compared with what was previously observed, as suggested by Willis (1985). Further studies are needed to examine competitive dynamics and how shifts in behavior and loss of species may alter these dynamics.

## Supplementary Material

arag111_Supplementary_Data

## Data Availability

Analyses reported in this article can be reproduced using the data provided by Howard et al. (2026).
