# Supplementary material for: Dominance and food availability alter avian space use at army ant swarms
Source: Behav Ecol. 2026 Oct 8;37(6):arag111. doi: 10.1093/beheco/arag111 (PMC13755867; doi:10.1093/beheco/arag111)
Supplement: Supplementary file 1 [file arag111_supplementary_data.docx]

Dominance and profitability alter avian space use in an iconic mixed-species group

## Elizabeth J. Howard, Andrés F. Ramírez-Mejía, Michael Castaño, Mary De Aquino, Henry S. Pollock, Jefferson Vizentin-Bugoni, and Corey E. Tarwater

Supporting information S1. Journal: Behavioral Ecology

Swarms were first drawn out. Point counts were then conducted, and location of birds were drawn out on swarms. Transects were then drawn out and walked to survey prey within swarms. Quadrat surveys were then drawn out and conducted. Finally, outside quadrats and transect surveys were conducted 15 m away from the swarm.

Figure S1: A diagram depicting methods used to survey bird location and potential prey lo-cation within swarms.

Table S1: Taxa seen consumed at swarms compared to taxa observed during prey surveys

| Taxa | Seen_consumed | Seen_during_prey_surveys | Prey_size_range | Sample_Size |
| --- | --- | --- | --- | --- |
| Anura | Yes | Yes | 10-48 | 3 |
| Aracnidae | Yes | Yes | <5-20 | 392 |
| Blattodea | Yes | No | <5-48 | 100 |
| Centipede | Yes | No | 40-56 | 8 |
| Coleoptera | Yes | Yes | <5-32 | 28 |
| Diptera | Yes | Yes | <5-16 | 1055 |
| Dyctioptera | Yes | Yes | <5-48 | 22 |
| Hemiptera | Yes | No | 8-32 | 9 |
| Heteroptera | Yes | No | 16 | 1 |
| Hymenoptera | Yes | Yes | <5-20 | 170 |
| Larvae | Yes | Yes | <5-16 | 25 |
| Lepidoptera | Yes | Yes | <5-32 | 48 |
| Myriapoda | Yes | No | 16-32 | 4 |
| Neuroptera | Yes | No | 16-32 | 3 |
| Odonata | Yes | Yes | <5-48 | 4 |
| Opillion | Yes | No | 32 | 1 |
| Isopoda | No | Yes | <5-10 | 4 |
| Orthoptera | Yes | Yes | <5-32 | 274 |
| Scorpiones | Yes | Yes | 10-40 | 14 |
| Vertebrate | No | Yes | <5 | 1 |

Prey observed being consumed by birds and potential prey observed during spatial surveys were similar in taxon and size. In particular, based on behavioral observations at swarms, we observed ant followers consuming prey from 20 different orders, with Diptera, Arachnidae, and Orthoptera being the most commonly consumed prey. Prey size varied substantially, with prey ranging from <5 mm to 58 mm in length (1.65mm ± 2.02mm). During prey surveys, prey ranged from <5mm to >20mm (5.16mm ± 3.14mm ). Eighteen of the 20 orders observed being consumed by birds were observed during the behavioral observations. The inly exception is one vertebrate seen in the prey surveys, while not being seen being consumed. During the potential prey surveys, we observed two additional orders that were not observed being consumed by ant followers.

Table S2: List of birds seen at swarms in Central Panama during 2023. Guild association based off Willis and Oniki 1978, Martinez et al. 2018

### English Common Name

**Scientific Name	Obligate, Fac-ultative, or Oc-casional**

Bicolored Antbird	*Gymnopithys bicolor*	Obligate Ocellated Antbird	*Phaenostictus mcleannani*	Obligate Grey-Headed Tanger	*Eucometis penicillata*	Facultative

Northern-barred Wood-	*Dendrocolaptes sanctithomae*	Facultative creeper

Spotted Antbird	*Hylophylax naevioides*	Facultative

Plain-Brown	Wood-	*Dendrocincla fuliginosa*	Facultative creeper

Cocoa Woodcreeper	*Xiphorhynchus susurrans*	Facultative Ruddy Woodcreeper	*Dendrocincla homochroa*	Occasional Black-bellied Wren	*Pheugopedius fasciatoventris* Occasional Dusky Antbird	*Cercomacra tyrannina*	Occasional

White-whiskered	Puff-	*Malacoptila panamensis*	Occasional bird

Yellow-rumped Cacique	*Cacicus cela*	Occasional Rufous Motmot	*Baryphthengus martii*	Occasional

White-breasted Wood-	*Henicorhina leucosticta*	Occasional Wren

Forest Elaenia	*Myiopagis gaimardii*	Occasional Red Capped Manakin	*Ceratopipra mentalis*	Occasional

Violet-bellied	Hum-mingbird

Chestnut-backed Antbird

*Chlorestes julie*	Occasional

*Poliocrania exsul*	Occasional

Song Wren	*Cyphorhinus phaeocephalus*	Occasional

Bay Wren	*Cantorchilus nigricapillus*	Occasional

Black-crowned Antshrike

*Thamnophilus atrinucha*	Occasional

Broad-billed Motmot *Electron platyrhynchum*	Occasional Southern Bentbill	*Oncostoma olivaceum*	Occasional Squirrel Cuckoo	*Piaya cayana*	Occasional

Scarlet-rumped Cacique

*Cacicus uropygialis*	Occasional

Cinnamon Woodpecker	*Celeus loricatus*	Occasional Black-faced Antthrush	*Formicarius analis*	Occasional

Table S2: Bird species observed at army ant swarms with their foraging relationships (contin-ued)

### English Common Name

Golden-crowned Spade-bill

### Scientific Name Obligate, Fac-ultative, or Oc-casional

*Platyrinchus coronatus*	Occasional

Black-breasted Puffbird *Notharchus pectoralis*	Occasional Dot-winged Antwren	*Microrhopias quixensis*	Occasional Stripe-throated Hermit *Phaethornis striigularis*	Occasional Keel-billed Toucan	*Ramphastos sulfuratus*	Occasional Bright-rumped Attila *Attila spadiceus*	Occasional Long-billed Gnatwren	*Ramphocaenus melanurus*	Occasional

Scaly-throated Leaftosser

Black-striped	Wood-creeper

*Sclerurus guatemalensis*	Occasional

*Xiphorhynchus lachrymosus*	Occasional

Lesser Greenlet	*Hylophilus decurtatus*	Occasional Black-throated Trogon *Trogon rufus*	Occasional

Slaty-tailed Trogon	*Trogon massena*	Occasional Long-billed Hermit	*Phaethornis longirostris*	Occasional Rufous Mourner	*Rhytipterna holerythra*	Occasional Brown-hooded Parrot	*Pyrilia haematotis*	Occasional

Checker-throated Stip-	*Epinecrophylla fulviventris*	Occasional plethroat

Scaly-breasted Wren	*Microcerculus marginatus*	Occasional

Purple-throated Fruitcrow

*Querula purpurata*	Occasional

Yellow-backed Oriole	*Icterus chrysater*	Occasional Green Shrike-Vireo	*Vireolanius pulchellus*	Occasional Olivaceous Flatbill	*Rhynchocyclus olivaceus*	Occasional Plain Xenops	*Xenops minutus*	Occasional Scrub Greenlet	*Hylophilus flavipes*	Occasional Purple-crowned Fairy	*Heliothryx barroti*	Occasional

Rufous-vented Ground	*Neomorphus geoffroyi*	Occasional Cuckoo

Spot-crowned Antvireo *Dysithamnus puncticeps*	Occasional Whooping Motmot	*Momotus subrufescens*	Occasional Masked Tityra	*Tityra semifasciata*	Occasional Crested Oropendola	*Psarocolius decumanus*	Occasional Crested Caracara	*Caracara plancus*	Occasional

Table S2: Bird species observed at army ant swarms with their foraging relationships (contin-ued)

### English Common Name

**Scientific Name	Obligate, Fac-ultative, or Oc-casional**

Fasciated Antshrike	*Cymbilaimus lineatus*	Occasional Tawny-faced Gnatwren	*Microbates cinereiventris*	Occasional

Ruddy-tailed	Fly-	*Terenotriccus erythrurus*	Occasional catcher

Stripe-throated Wren	*Cantorchilus leucopogon*	Occasional White-tailed Trogon	*Trogon chionurus*	Occasional

Great Tinamou	*Tinamus major*	Occasional

Buff-throated	Foliage-gleaner

Chestnut-headed Oropendola

*Automolus ochrolaemus*	Occasional

*Psarocolius wagleri*	Occasional

Crowned Woodnymph	*Thalurania colombica*	Occasional

White-shouldered Tan-	*Tachyphonus luctuosus*	Occasional ager

Velvety Manakin	*Lepidothrix coronata*	Occasional

Table S3: Table of TaxReg coeﬀicients used to calculate prey biomass.

| Taxonomic_Grouping | Coeﬀicient_Used |
| --- | --- |
| Anura | NA |
| Aracnidae | 2.6110 |
| Coleoptera | 2.6160 |
| Diptera | 2.2710 |
| Dyctioptera | 1.9130 |
| Hymenoptera | 2.7920 |
| Isopoda | 2.8390 |
| Larva | 0.1315 |
| Lepidoptera | 2.6370 |
| Odonata | 1.7030 |
| Orthoptera | 2.2050 |
| Other* | 2.3990 |
| Scorpiones | 2.0150 |
| Vertebrate | NA |

These coeﬀicients were used in the equation (log(mass)~TaxReg x log(length) from Solhström et al. 2018. TaxReg coeﬀicients were created to be a factorial variable based off taxonomic group and geographic region factorial independent variables (Solhström et al. 2018).

Dominance and profitability alter avian space use in an iconic mixed-species group

## Elizabeth J. Howard, Andrés F. Ramírez-Mejía, Michael Castaño, Mary De Aquino, Henry S. Pollock, Jefferson Vizentin-Bugoni, and Corey E. Tarwater

Supporting information S2. Journal: Behavioral Ecology

# Table of contents

S1 Introduction	2

S2 Model species	3

S2.1 Obligate species	3

S2.2 Facultative species	5

S3 Data wrangling	6

S3.1 Swarm area data	7

S3.2 Quadrants data	7

S3.3 Transect data	7

S3.4 Merging quadrant and transect data	8

S3.5 Birds data	8

S4 Model 1: prey biomass	10

S4.1 Data preparation	10

S4.2 Mathematical notation	12

S4.3 Stan code	12

S4.4 Fitting procedure	14

S4.5 Summary	15

S4.6 Sampling diagnostics	17

S4.7 Posterior distributions	18

S5 Merging prey and bird data	23

S6 Model 2: effect of prey biomass and swarm area on ant-followers space use (assemblage level)	25

S6.1 Data preparation	25

S6.2 Mathematical notation	26

S6.3 Fitting procedure	33

S6.4 Summary	33

S6.5 Sampling diagnostics	35

S6.6 Posterior distributions	36

S6.6.1 Effect of swarm on prey biomass	37

S6.6.2 Effect of swarm area on ant-followers	40

S6.6.3 Effect of prey biomass on ant-followers	42

S6.6.4 Space use across sections	44

S7 Model 3: effect of dominance on ant-followers space use (species level)	48

S7.1 Data preparation	48

S7.2 Mathematical notation	49

S7.3 Stan code	51

S7.4 Fitting procedure	60

S7.5 Summary	61

S7.6 Sampling diagnostics	65

S7.7 Posterior distributions	67

S7.7.1 Effect of swarm area on ant-follower species	69

S7.7.2 Effect of profitability on ant-followers presence	72

S7.7.3 Effect of dominance on the space use by ant followers	75

S7.7.3.1 𝑃 . 𝑚𝑐𝑙𝑒𝑎𝑛𝑛𝑎𝑛𝑖 space use	76

S7.7.3.2 𝐸. 𝑝𝑒𝑛𝑖𝑐𝑖𝑙𝑙𝑎𝑡𝑎 space use	77

S7.7.3.3 𝐺. 𝑏𝑖𝑐𝑜𝑙𝑜𝑟 space use	79

S7.7.3.3.1 Contrast when 𝐺. 𝑏𝑖𝑐𝑜𝑙𝑜𝑟 is alone	82

S7.7.3.3.2 Contrast when 𝐺. 𝑏𝑖𝑐𝑜𝑙𝑜𝑟 is with other species	84

S7.7.3.4 𝐻. 𝑛𝑎𝑒𝑣𝑖𝑜𝑖𝑑𝑒𝑠 space use	85

S8 Computational envionment	87

# S1 Introduction

This document reproduces the fitting procedure of Bayesian models from the article **Testing long standing assumptions about army ant swarms: how profitability and domi-nance alter space use in birds**. It is organized into sections showing model species, data wrangling operations for preparing the data, models in mathematical notation, Stan code, fitting procedure, models diagnostics, and simulations of interventions.

# S2 Model species

## S2.1 Obligate species

Figure S1: Phaenostictus mcleannan

Figure S2: Gymnopithys bicolor

## S2.2 Facultative species

Figure S3: Hylophylax naevioides

Figure S4: Eucometis penicillata

# S3 Data wrangling

We divided the swarm into eight sections (figure below) and estimated the variation of arthro-pod biomass among them. To do so, I considered the *quadrant* as the sampling unit and pulled together the data from quadrants and transects to fit the model.

The following code conduct wrangling processes to merge data from swarm area, individual quadrants and quadrants within transects. The quadrants holds the data on prey biomass.

pks <- c('readxl', 'dplyr', 'patchwork', 'tibble',

'lubridate', 'ggplot2', 'dplyr', 'forcats', 'magrittr', 'cmdstanr', 'patchwork', 'rethinking', 'tidyr', 'cowplot')

sapply(pks, library, character.only = T)

source('functions_mod_diagnostics.r') extrafont::loadfonts(device="win")

## S3.1 Swarm area data

Loading swarm area data. Here conducted formatting of date objects and columns filtering.

swarm_area <- read_xlsx('2023_General_Data.xlsx', col_names = T, sheet = 2)

colnames(swarm_area) <-

c('swarm_ID', 'day', 'month', 'year', 'round_number', 'time_round', 'area', 'center_square', 'notes')

swarm_area$date <- as.Date(swarm_area %$% paste('2023', month, day, sep = '-'),

format = '%Y-%b-%d')

swarm_area <- swarm_area[, c("date", "swarm_ID", "round_number", "area")]

## S3.2 Quadrants data

Code for formatting labels and date object.

quadrants <- as_tibble(read.table('quadrat_forandres.txt', sep = ',', dec = '.',

header = T))

quadrants <- split(quadrants,

quadrants %$% list(Antswarm_ID,

Day,

Month, Round))

t <- unlist(lapply(quadrants, **function**(x) nrow(x) > 0))

quadrants <- quadrants[t]

quadrants <- lapply(quadrants, FUN =

**function**(x) {

x <- x[, -c(7, 8)]

unique(x)

})

quadrants <- do.call('rbind', quadrants)

**for** (i **in** grep('Category', colnames(quadrants))) { quadrants[[i]] <- toupper(quadrants[[i]])

}

quadrants <- quadrants[quadrants$Category_Andres != '', ] colnames(quadrants)[1:4] <- c('swarm_ID', 'day', 'month', 'round_number') quadrants$date <- as.Date(quadrants %$% paste('2023', month, day, sep = '-'),

format = '%Y-%b-%d')

## S3.3 Transect data

transect <- as_tibble(read.table('transect_forandres.txt', sep = ',', dec = '.',

transect <- split(transect,

header = T))

transect %$% list(Antswarm_ID,

Day,

Month, Round))

t <- unlist(lapply(transect, **function**(x) nrow(x) > 0)) transect <- transect[t]

transect <- lapply(transect, FUN =

**function**(x) {

x <- x[, -c(8, 10)]

unique(x)

})

transect <- do.call('rbind', transect)

**for** (i **in** grep('Category', colnames(transect))) { transect[[i]] <- toupper(transect[[i]])

}

transect$date <- as.Date(transect %$% paste('2023', Month, Day, sep = '-'),

format = '%Y-%b-%d')

transect <-transect |>

group_by(date, Antswarm_ID, Round, Category_Corey, Transect_number) |> transmute(prey_biomass = mean(total_biomass_square_or_outside)) |> unique()

## S3.4 Merging quadrant and transect data

transect1 <- transect[, c("date", "Antswarm_ID", "Round", "Category_Corey",

"prey_biomass")]

colnames(transect1) <- c('date', 'swarm_ID', 'round_number', 'categories',

'prey_biomass')

quadrants1 <- quadrants[, c("date", "swarm_ID", "round_number",

"Category_Corey", "biomass_square")]

colnames(quadrants1) <- c('date', 'swarm_ID', 'round_number', 'categories',

'prey_biomass')

transect1$type <- 'transect' quadrants1$type <- 'quadrant'

prey <- rbind(transect1, quadrants1)

## S3.5 Birds data

In this section we join birds presence and swarm area. We conducted data formatting tasks to correct label of species categories, dates and sampling codes. Also we generated a distance sampling of bird sampling.

birds <- as_tibble(read.csv('bird_forandres.csv', header = T)) birds <- birds[, 1:grep('Notes', colnames(birds))] colnames(birds)[11] <- 'bird_category'

**for** (i **in** grep('Category', colnames(birds))) { birds[[i]] <- toupper(birds[[i]])

}

birds <- birds[birds$Category_Corey != '', ]

birds$Category_Andres[which(birds$Category_Andres == '')] <- 'FP' birds$Category_Andres[which(birds$Category_Andres == '0')] <- 'O' birds$Category_Corey[which(birds$Category_Corey == '0')] <- 'O' birds$bird_category[which(birds$bird_category == '0')] <- 'O'

birds$date <- as.Date(birds %$% paste('2023', Month, Day, sep = '-'),

format = '%Y-%b-%d')

birds <- birds[birds$Bird_Code != '0', ]

birds2 <- unique(birds[, c("date", "Antswarm_ID", "Round_Number")]) birds2 <- lapply(1:nrow(birds2), FUN =

**function**(x) {

codes <- unique(birds$Category_Corey)

x <- apply(birds2[x, ], 2, **function**(i) rep(i, length(codes))) x <- as_tibble(x)

x$categories <- codes d <- x$date[1]

cod <- x$Round_Number[1] df2 <-

birds[birds$date == d &

birds$Round_Number == cod, ] x$date <- as.Date(x$date)

df2 <- df2[, c("date", "Antswarm_ID", "Bird_Code", 'Category_Corey', 'bird_category')]

colnames(df2)[4] <- 'categories'

df3 <- full_join(df2, x, by = c('date', 'Antswarm_ID', 'categories')) df3$Bird_Code[is.na(df3$Bird_Code)] <- 'no_bird' df3$bird_category[is.na(df3$bird_category)] <- 'no_bird'

df3

})

birds2 <- do.call('rbind', birds2) birds2 <-

birds2 |>

mutate(total = ifelse(birds2$bird_category != 'no_bird', 1, 0), obligate = ifelse(birds2$bird_category == 'O', 1, 0), facultative = ifelse(birds2$bird_category == 'F', 1, 0), occasional = ifelse(birds2$bird_category == 'C', 1, 0), BIAN = ifelse(birds2$Bird_Code == 'BIAN', 1, 0),

GHET = ifelse(birds2$Bird_Code == 'GHET', 1, 0), OCAN = ifelse(birds2$Bird_Code == 'OCAN', 1, 0), SPAN = ifelse(birds2$Bird_Code == 'SPAN', 1, 0)) |>

group_by(date, Antswarm_ID, categories, Round_Number) |> mutate(n_total = sum(total),

n_obligate = sum(obligate),

BIAN_s = sum(Bird_Code == 'BIAN'), GHET_s = sum(Bird_Code == 'GHET'), OCAN_s = sum(Bird_Code == 'OCAN'), SPAN_s = sum(Bird_Code == 'SPAN'))

swarm_area$round_number <- as.character(swarm_area$round_number) birds2 <- split(birds2, list(birds2$date, birds2$Antswarm_ID))

birds2$`2023-06-14.MDAEB13`$Round_Number[is.na(birds2$`2023-06-14.MDAEB13`$Round_Number)] <- '3'

birds2$`2023-06-14.CCDEB06`$Round_Number[is.na(birds2$`2023-06-14.CCDEB06`$Round_Number)] <- '1' birds2 <- do.call('rbind', birds2)

swarm_area <- swarm_area[(swarm_area$date %in% birds2$date), ]

colnames(birds2)[c(1, 2, 6)] <- colnames(swarm_area)[1:3] birds3 <- right_join(swarm_area, birds2,

by = c('date', 'swarm_ID', 'round_number'))

colnames(birds3)[4] <- 'swarm_area' birds3$date_fct <- as.factor(birds3$date) days_birds <- sort(unique(birds3$date))

dist_day_bird <- # distance matrix of sampling days of bird observations

sapply(days_birds, FUN =

**function**(i) {

sapply(days_birds, FUN =

})

**function**(j) { abs(as.numeric(i - j))

})

colnames(dist_day_bird) <- levels(birds3$date_fct) rownames(dist_day_bird) <- levels(birds3$date_fct)

# S4 Model 1: prey biomass

This model assess the variation of prey biomass across swarm sections. Below, we provide the code for data formatting, to generate a distance matrix of sampling dates, model in mathe-matical notation, Stan code, fitting procedure and model diagnostics.

## S4.1 Data preparation

prey$date_fct <- as.factor(prey$date)

prey$type <- as.factor(prey$type) day_prey <- sort(unique(prey$date))

dist_day_prey <- # distance matrix

sapply(day_prey, FUN =

**function**(i) {

sapply(day_prey, FUN =

})

**function**(j) { abs(as.numeric(i - j))

})

colnames(dist_day_prey) <- levels(prey$date_fct) rownames(dist_day_prey) <- levels(prey$date_fct)

dat_prey <-

lapply(prey, FUN =

**function**(x) {

**if** (is.numeric(x)) x

**else** as.numeric(as.factor(as.character(x)))

})

dat_prey$z_prey_biomass <- as.vector(scale(dat_prey$prey_biomass)) dat_prey$N <- length(dat_prey$date)

dat_prey$N_date <- max(dat_prey$date) dat_prey$N_swarm <- max(dat_prey$swarm_ID) dat_prey$N_round_number <- max(dat_prey$round_number) dat_prey$N_categories <- max(dat_prey$categories) dat_prey$dist_day <- dist_day_prey

dat_prey$prey_biomass <- dat_prey$prey_biomass + 0.1 dat_prey$log_prey_biomass <- log(dat_prey$prey_biomass)

dat_prey$log_prey_biomass[which(dat_prey$log_prey_biomass <= 0)] <-0.001

dat_prey$samp_eff_std <- ifelse(dat_prey$type == 1, 0.5, 1) dat_prey$samp_eff_std <- dat_prey$samp_eff_std / mean(dat_prey$samp_eff_std)

## S4.2 Mathematical notation

𝑃 𝑟𝑒𝑦 𝑏𝑖𝑜𝑚𝑎𝑠𝑠 ∼ 𝐿𝑜𝑔𝑁𝑜𝑟𝑚𝑎𝑙(𝜇_𝑖_, 𝜎)

𝑙𝑜𝑔(𝜇_𝑖_) = 𝛼_𝑠𝑒𝑐𝑡𝑖𝑜𝑛_ _𝑖_ + 𝜃_𝑠𝑤𝑎𝑟𝑚_ _𝑖_ + 𝜏_𝑟𝑜𝑢𝑛𝑑_ _𝑖_ + 𝜙_𝑑𝑎𝑡𝑒_ _𝑖_ + 𝑙𝑜𝑔(𝑠𝑎𝑚𝑝𝑙𝑖𝑛𝑔 𝑒𝑓𝑓𝑜𝑟𝑡_𝑖_)

𝛼𝑠𝑒𝑐𝑡𝑖𝑜𝑛 𝑖 = 𝜇𝛼 + 𝑧𝛼 × 𝜎𝛼

𝑧_𝛼_ ∼ 𝑁𝑜𝑟𝑚𝑎𝑙(3.5, 0.5)

𝜇_𝛼_ ∼ 𝑁𝑜𝑟𝑚𝑎𝑙(0, 0.25)

𝜎_𝛼_ ∼ 𝐸𝑥𝑝𝑜𝑛𝑒𝑛𝑡𝑖𝑎𝑙(1)

𝜏𝑟𝑜𝑢𝑛𝑑 𝑖 = 𝜇𝜏 + 𝑧𝜏 × 𝜎𝜏

𝑧_𝜏_ ∼ 𝑁𝑜𝑟𝑚𝑎𝑙(0, 0.25)

𝜇_𝜏_ ∼ 𝑁𝑜𝑟𝑚𝑎𝑙(0, 0.25)

𝜎_𝜏_ ∼ 𝐸𝑥𝑝𝑜𝑛𝑒𝑛𝑡𝑖𝑎𝑙(1)

𝜃𝑠𝑤𝑎𝑟𝑚 𝑖 = 𝜇𝜃 + 𝑧𝜃 × 𝜎𝜃

𝑧_𝜃_ ∼ 𝑁𝑜𝑟𝑚𝑎𝑙(0, 0.25)

𝜇_𝜃_ ∼ 𝑁𝑜𝑟𝑚𝑎𝑙(0, 0.25)

𝜎_𝜃_ ∼ 𝐸𝑥𝑝𝑜𝑛𝑒𝑛𝑡𝑖𝑎𝑙(1)

⎛ 𝜙𝑑𝑎𝑡𝑒 1 ⎞

⎛ ⎡ 0 ⎤	⎞

⎜ 𝜙𝑑𝑎𝑡𝑒 2 ⎟ ∼ 𝑀𝑉 𝑁𝑜𝑟𝑚𝑎𝑙 ⎜ ⎢ 0 ⎥ 𝐾_𝜙_ ⎟

⎜	...	⎟	⎜ ⎢...⎥	⎟

⎝ 𝜙𝑑𝑎𝑡𝑒 𝑛 ⎠

⎝ ⎣ 0 ⎦	⎠

𝐾_𝜙_ = 𝜂2 𝑒𝑥𝑝(−𝜌2 𝐷2	)+ 𝛿_𝜙_ _𝑖𝑗_ × 0.01

𝜙	𝜙 𝑑𝑎𝑡𝑒𝑠 𝑖𝑗

𝜂_𝜙_ ∼ 𝐸𝑥𝑝𝑜𝑛𝑒𝑛𝑡𝑖𝑎𝑙(1)

𝜌_𝜙_ ∼ 𝐸𝑥𝑝𝑜𝑛𝑒𝑛𝑡𝑖𝑎𝑙(1)

𝜎 ∼ 𝐸𝑥𝑝𝑜𝑛𝑒𝑛𝑡𝑖𝑎𝑙(1)

The above Bayesian hierarchical model uses a 𝐿𝑜𝑔𝑁𝑜𝑟𝑚𝑎𝑙 likelihood to model log-scaled prey biomass as function of *swarm section*, *swarm identity*, *sampling round*, and *sampling site*. We used Gaussian processes to estimate to estimate 𝜙 parameter and account for temporal correlation. Other grouping factors were estimated using partial polling and a non-centered parametrization.

## S4.3 Stan code

cat(file = 'prey_model.stan',

"

functions {

matrix cov_GPL2(matrix x,

real eta, real rho,

real delta) {

int N = dims(x)[1];

matrix[N, N] k;

for (i in 1:(N-1)) { k[i, i] = eta + delta; for (j in (i + 1):N) {

k[i, j] = eta * exp(-rho * square(x[i, j])); k[j, i] = k[i, j];

}

}

}

data {

int N;

int N_date; int N_swarm;

k[N, N] = eta + delta; return k;

}

int N_round_number; int N_categories;

matrix[N_date, N_date] dist_day; array[N] int date;

array[N] int swarm_ID; array[N] int round_number; array[N] int categories; vector[N] prey_biomass; vector[N] z_prey_biomass; vector[N] log_prey_biomass; vector[N] samp_eff_std;

}

parameters { vector[N_categories] z_alpha; real mu_alpha;

real<lower = 0> sigma_alpha;

vector[N_round_number] z_tau; real mu_tau;

real<lower = 0> sigma_tau;

vector[N_swarm] z_theta; real mu_theta;

real<lower = 0> sigma_theta;

vector[N_date] z_phi; real<lower = 0> eta; real<lower = 0> rho;

} real<lower = 0> sigma;

transformed parameters { vector[N_date] phi; matrix[N_date, N_date] sigma_phi;

matrix[N_date, N_date] L_sigma_phi;

sigma_phi = cov_GPL2(dist_day, eta, rho, 0.001); L_sigma_phi = cholesky_decompose(sigma_phi);

phi = L_sigma_phi * z_phi;

vector[N_categories] alpha;

alpha = mu_alpha + z_alpha * sigma_alpha;

vector[N_round_number] tau;

tau = mu_tau + z_tau * sigma_tau;

vector[N_swarm] theta;

theta = mu_theta + z_theta * sigma_theta;

}

model { vector[N] mu;

z_alpha ~ normal(3.5, 0.5); mu_alpha ~ normal(0, 0.25); sigma_alpha ~ exponential(1);

z_tau ~ normal(0, 0.25); mu_tau ~ normal(0, 0.25); sigma_tau ~ exponential(1);

z_theta ~ normal(0, 0.25); mu_theta ~ normal(0, 0.25); sigma_theta ~ exponential(1);

z_phi ~ normal(0, 0.25); rho ~ exponential(1); eta ~ exponential(1);

sigma ~ exponential(1); for (i in 1:N) {

prey_biomass[i] ~ lognormal(alpha[categories[i]] +

theta[swarm_ID[i]] + tau[round_number[i]] + phi[date[i]] + log(samp_eff_std[i]), sigma);

}

}

generated quantities { vector[N] mu;

array[N] real ppcheck;

for (i in 1:N) {

mu[i] = alpha[categories[i]] + theta[swarm_ID[i]] + tau[round_number[i]] +

phi[date[i]] + log(samp_eff_std[i]);

}

ppcheck = lognormal_rng(mu, sigma);

} ")

## S4.4 Fitting procedure

file <- paste0(getwd(), '/prey_model.stan')

fit_prey <- cmdstan_model(file, compile = T)

mod_prey <-fit_prey$sample(

data = dat_prey, iter_warmup = 500, iter_sampling = 2e3, thin = 3,

chains = 3,

parallel_chains = 3,

refresh = 500,

seed = 555

)

## S4.5 Summary

summary_prey <-

mod_prey$summary(c('alpha', 'tau', 'theta', 'phi', 'sigma'))

text <- 'Estimated coefficients and sampling diagnostics

of the model evaluating the variation of prey biomass across swarm sections'

knitr::kable(

summary_prey, digits = 2, caption = text

)

Table S1: Estimated coeﬀicients and sampling diagnostics of the model evaluating the variation of prey biomass across swarm sections

| variable | mean | median | sd | mad | q5 | q95 | rhat | ess_bulk | ess_tail |
| --- | --- | --- | --- | --- | --- | --- | --- | --- | --- |
| alpha[1] | 3.52 | 3.52 | 0.33 | 0.32 | 2.99 | 4.07 | 1 | 1904.60 | 1500.83 |
| alpha[2] | 2.58 | 2.57 | 0.33 | 0.33 | 2.05 | 3.12 | 1 | 2091.07 | 1879.08 |
| alpha[3] | 3.71 | 3.70 | 0.32 | 0.32 | 3.18 | 4.24 | 1 | 2059.50 | 1762.18 |
| alpha[4] | 2.59 | 2.59 | 0.32 | 0.33 | 2.07 | 3.11 | 1 | 1981.66 | 1474.48 |
| alpha[5] | 2.56 | 2.55 | 0.32 | 0.33 | 2.03 | 3.09 | 1 | 1947.22 | 1391.58 |
| alpha[6] | 2.56 | 2.56 | 0.32 | 0.33 | 2.04 | 3.09 | 1 | 1959.95 | 1672.26 |
| alpha[7] | 3.03 | 3.02 | 0.33 | 0.33 | 2.50 | 3.57 | 1 | 2020.91 | 1569.95 |
| alpha[8] | 3.62 | 3.62 | 0.33 | 0.34 | 3.10 | 4.19 | 1 | 2076.24 | 1951.16 |
| tau[1] | -0.07 | -0.07 | 0.24 | 0.24 | -0.46 | 0.32 | 1 | 1943.22 | 1729.65 |
| tau[2] | -0.06 | -0.06 | 0.24 | 0.24 | -0.46 | 0.33 | 1 | 1928.23 | 1793.75 |
| tau[3] | -0.06 | -0.06 | 0.24 | 0.24 | -0.45 | 0.33 | 1 | 1949.79 | 1728.62 |
| tau[4] | -0.04 | -0.05 | 0.24 | 0.24 | -0.43 | 0.35 | 1 | 1923.70 | 1862.38 |
| tau[5] | -0.05 | -0.05 | 0.24 | 0.24 | -0.44 | 0.34 | 1 | 2021.97 | 1897.26 |
| tau[6] | -0.03 | -0.03 | 0.24 | 0.25 | -0.42 | 0.37 | 1 | 1943.36 | 1829.57 |
| tau[7] | -0.05 | -0.05 | 0.24 | 0.24 | -0.45 | 0.35 | 1 | 1963.22 | 1814.22 |

| variable | mean | median | sd | mad | q5 | q95 | rhat | ess_bulk | ess_tail |
| --- | --- | --- | --- | --- | --- | --- | --- | --- | --- |
| tau[8] | -0.05 | -0.06 | 0.24 | 0.24 | -0.45 | 0.34 | 1 | 1914.49 | 1829.03 |
| theta[1] | -0.03 | -0.04 | 0.25 | 0.26 | -0.44 | 0.38 | 1 | 2086.86 | 1899.49 |
| theta[2] | -0.06 | -0.05 | 0.25 | 0.25 | -0.46 | 0.35 | 1 | 2048.48 | 1945.40 |
| theta[3] | -0.05 | -0.05 | 0.25 | 0.25 | -0.46 | 0.37 | 1 | 2043.66 | 1814.49 |
| theta[4] | -0.01 | -0.01 | 0.25 | 0.25 | -0.41 | 0.41 | 1 | 2025.87 | 2002.76 |
| theta[5] | -0.09 | -0.08 | 0.26 | 0.26 | -0.50 | 0.33 | 1 | 2057.08 | 1800.60 |
| theta[6] | 0.03 | 0.02 | 0.26 | 0.26 | -0.40 | 0.47 | 1 | 1925.16 | 1828.13 |
| theta[7] | -0.03 | -0.04 | 0.25 | 0.26 | -0.45 | 0.38 | 1 | 2109.11 | 1967.45 |
| theta[8] | -0.04 | -0.04 | 0.25 | 0.25 | -0.44 | 0.37 | 1 | 2003.01 | 1871.00 |
| theta[9] | -0.08 | -0.07 | 0.25 | 0.25 | -0.49 | 0.34 | 1 | 2032.78 | 1867.96 |
| theta[10] | -0.06 | -0.05 | 0.26 | 0.25 | -0.49 | 0.36 | 1 | 1927.42 | 1750.82 |
| theta[11] | -0.04 | -0.03 | 0.25 | 0.25 | -0.45 | 0.37 | 1 | 2019.84 | 1939.30 |
| theta[12] | -0.07 | -0.07 | 0.25 | 0.25 | -0.47 | 0.35 | 1 | 1961.81 | 1918.36 |
| theta[13] | -0.13 | -0.12 | 0.26 | 0.25 | -0.55 | 0.30 | 1 | 1973.76 | 1895.10 |
| theta[14] | -0.02 | -0.02 | 0.25 | 0.25 | -0.42 | 0.39 | 1 | 2002.09 | 1895.27 |
| phi[1] | -0.20 | -0.20 | 0.13 | 0.13 | -0.41 | 0.01 | 1 | 1777.37 | 1969.52 |
| phi[2] | -0.20 | -0.20 | 0.15 | 0.16 | -0.44 | 0.04 | 1 | 1539.83 | 1862.75 |
| phi[3] | -0.34 | -0.33 | 0.17 | 0.16 | -0.64 | -0.07 | 1 | 1667.78 | 1812.26 |
| phi[4] | -0.10 | -0.10 | 0.15 | 0.15 | -0.35 | 0.15 | 1 | 1876.25 | 1940.93 |
| phi[5] | 0.12 | 0.12 | 0.14 | 0.14 | -0.10 | 0.37 | 1 | 1963.23 | 1745.87 |
| phi[6] | -0.09 | -0.10 | 0.12 | 0.12 | -0.30 | 0.10 | 1 | 1919.48 | 1638.03 |
| phi[7] | -0.01 | -0.02 | 0.15 | 0.14 | -0.25 | 0.23 | 1 | 2051.40 | 1853.34 |
| phi[8] | -0.02 | -0.02 | 0.14 | 0.14 | -0.25 | 0.22 | 1 | 1945.77 | 1820.27 |
| phi[9] | 0.10 | 0.09 | 0.13 | 0.13 | -0.12 | 0.32 | 1 | 1873.13 | 2002.28 |
| phi[10] | -0.06 | -0.05 | 0.12 | 0.12 | -0.26 | 0.14 | 1 | 1958.97 | 1962.61 |
| phi[11] | -0.17 | -0.16 | 0.15 | 0.14 | -0.41 | 0.06 | 1 | 1888.22 | 1812.52 |
| phi[12] | -0.09 | -0.09 | 0.14 | 0.13 | -0.33 | 0.13 | 1 | 2019.74 | 1856.25 |
| phi[13] | -0.04 | -0.03 | 0.14 | 0.13 | -0.26 | 0.18 | 1 | 1943.40 | 1736.71 |
| phi[14] | 0.03 | 0.03 | 0.12 | 0.12 | -0.17 | 0.23 | 1 | 1924.16 | 1889.72 |
| phi[15] | 0.13 | 0.12 | 0.13 | 0.13 | -0.08 | 0.35 | 1 | 1639.45 | 1890.11 |
| phi[16] | -0.03 | -0.03 | 0.13 | 0.13 | -0.24 | 0.19 | 1 | 2037.51 | 2046.99 |
| phi[17] | -0.11 | -0.11 | 0.12 | 0.12 | -0.30 | 0.09 | 1 | 1793.92 | 1809.54 |
| phi[18] | -0.08 | -0.08 | 0.14 | 0.13 | -0.32 | 0.13 | 1 | 1678.16 | 1879.66 |
| phi[19] | 0.04 | 0.04 | 0.12 | 0.11 | -0.15 | 0.23 | 1 | 2059.63 | 2016.35 |
| phi[20] | -0.06 | -0.06 | 0.13 | 0.12 | -0.27 | 0.14 | 1 | 1966.51 | 2054.08 |
| phi[21] | -0.14 | -0.13 | 0.18 | 0.17 | -0.46 | 0.12 | 1 | 1662.76 | 1774.41 |
| phi[22] | 0.03 | 0.03 | 0.14 | 0.13 | -0.20 | 0.25 | 1 | 1950.39 | 1986.73 |
| phi[23] | 0.08 | 0.08 | 0.12 | 0.11 | -0.10 | 0.29 | 1 | 1904.87 | 1777.90 |
| phi[24] | 0.11 | 0.11 | 0.12 | 0.12 | -0.08 | 0.31 | 1 | 1906.67 | 1766.43 |
| phi[25] | 0.19 | 0.19 | 0.12 | 0.12 | -0.01 | 0.39 | 1 | 1929.23 | 1969.96 |
| phi[26] | 0.09 | 0.09 | 0.12 | 0.12 | -0.11 | 0.29 | 1 | 1782.51 | 1807.04 |

Rhat

0.999

1.001

| variable | mean | median | sd | mad | q5 | q95 | rhat | ess_bulk | ess_tail |
| --- | --- | --- | --- | --- | --- | --- | --- | --- | --- |
| phi[27] | -0.03 | -0.03 | 0.11 | 0.11 | -0.21 | 0.16 | 1 | 2094.28 | 1857.46 |
| phi[28] | 0.30 | 0.31 | 0.15 | 0.14 | 0.05 | 0.53 | 1 | 1744.36 | 1717.01 |
| sigma | 0.76 | 0.76 | 0.02 | 0.02 | 0.73 | 0.79 | 1 | 2112.20 | 1930.08 |

## S4.6 Sampling diagnostics

mod_diagnostics(mod_prey, summary_prey)

0	500	1000	1500	2000

ESS parameters (tail and bulk)

Figure S5: Bulk and tail effective sampling size (ESS) of the model evaluating the variation of prey biomass across swarm sections

ppcheck_prey <- mod_prey$draws('ppcheck', format = 'matrix')

plot(density(dat_prey$prey_biomass), main = '', xlab = 'Prey biomass')

**for** (i **in** 1:100) { lines(density(ppcheck_prey[i, ]), lwd = 0.1)

}

lines(density(dat_prey$prey_biomass), col = 'red')

0.030

0	50	100	150

Density

0.000

0.015

Prey biomass

Figure S6: Posterior predictive checks of the model evaluating the variation of prey biomass across swarm sections

## S4.7 Posterior distributions

Here we extract the posterior distributions of the parameters and used the model structure to plot the effect of swarm section on prey biomass while marginalizing grouping predictors.

post_prey <-

mod_prey$draws(c('alpha', 'tau', 'theta', 'phi', 'sigma'), format = 'df')

post_prey <-

list(alpha = post_prey[, grep('alpha', colnames(post_prey))], tau = post_prey[, grep('tau', colnames(post_prey))], theta = post_prey[, grep('theta', colnames(post_prey))], phi = post_prey[, grep('phi', colnames(post_prey))], sigma = post_prey$sigma)

est_prey <-

lapply(seq_along(post_prey$alpha), FUN =

**function**(x) {

indx <- which(dat_prey$categories == x) dates <- unique(dat_prey$date[indx]) s_id <- unique(dat_prey$swarm_ID[indx])

round_n <- unique(dat_prey$round_number[indx]) samp_eff <- dat_prey$samp_eff_std[indx]

mu <-

with(post_prey,

{

alpha[, x, drop = T] + apply(theta[, s_id], 1, mean) + apply(tau[, round_n], 1, mean) + apply(phi[, dates], 1, mean) + log(mean(samp_eff))

})

tibble(mean = exp(mu),

category = unique(prey$categories[indx]))

})

est_prey1 <- do.call('rbind', est_prey)

est_prey1 |>

ggplot(aes(fct_reorder(category, mean), mean)) + geom_boxplot() +

labs(y = 'Prey biomass (gr)', x = NULL)

35

30

25

Prey biomass (gr)

20

15

IF	IS	O	IB	C	BP	SP	FP

Figure S7: Arthropod biomass (gr) across eight sections of army-ant swarms. O = outside, IF

= interior front, IS = interior side, C = center, IB = interior border, BP = back perimeter, SP = side perimeter, FP = front perimeter

Contrast of prey biomass between swarm sections

codes_categories <-

unique(tibble(x = dat_prey$categories,

x2 = prey$categories))

difference; diff_

codes_categories <- codes_categories[order(codes_categories$x), ] names(est_prey) <- codes_categories$x2

p_prey <-

lapply(names(est_prey), FUN =

**function**(name) { do.call('rbind',

lapply(est_prey, FUN =

})

**function**(x) {

j <- ((est_prey[[name]]$mean - x$mean)/x$mean)*100

df <-

tibble(mu = mean(j),

li = quantile(j, 0.025), ls = quantile(j, 0.975),

p_higher = mean(est_prey[[name]]$mean > x$mean), category = paste0(name, '-', x$category[1]),

var = name)

df[df$mu != 0, ]

}))

names(p_prey) <- names(est_prey)

knitr::kable(do.call('rbind', p_prey), digits = 3,

caption = 'Contrast of prey biomass among sections of the swarm. diff_mu: average

Table S2: Contrast of prey biomass among sections of the swarm. diff_mu: average difference; diff_sd = SD of the difference; p: probability that the diference section i and section j is above zero; section: section used to estimated the contrast. O = outside, IF

= interior front, IS = interior side, C = center, IB = interior border, BP = back perimeter, SP = side perimeter, FP = front perimeter

| mu | li | ls | p_higher | category | var |
| --- | --- | --- | --- | --- | --- |
| 41.615 | 10.976 | 75.899 | 0.999 | BP-C | BP |
| -19.381 | -32.448 | -3.952 | 0.006 | BP-FP | BP |
| 42.479 | 16.038 | 73.285 | 1.000 | BP-IB | BP |
| 45.907 | 17.583 | 79.185 | 1.000 | BP-IF | BP |
| 44.324 | 17.696 | 73.424 | 1.000 | BP-IS | BP |
| 42.415 | 16.072 | 73.765 | 0.999 | BP-O | BP |
| -6.060 | -24.084 | 14.601 | 0.265 | BP-SP | BP |
| -28.362 | -43.149 | -9.891 | 0.001 | C-BP | C |
| -42.530 | -53.997 | -28.612 | 0.000 | C-FP | C |
| 1.571 | -20.269 | 27.687 | 0.512 | C-IB | C |
| 4.015 | -19.132 | 32.434 | 0.596 | C-IF | C |
| 2.878 | -19.579 | 29.051 | 0.572 | C-IS | C |
| 1.502 | -20.131 | 28.210 | 0.527 | C-O | C |
| -33.040 | -47.930 | -15.120 | 0.000 | C-SP | C |

| mu | li | ls | p_higher | category | var |
| --- | --- | --- | --- | --- | --- |
| 25.025 | 4.114 | 48.034 | 0.994 | FP-BP | FP |
| 76.183 | 40.080 | 117.377 | 1.000 | FP-C | FP |
| 77.270 | 47.606 | 111.407 | 1.000 | FP-IB | FP |
| 81.606 | 48.656 | 120.088 | 1.000 | FP-IF | FP |
| 79.591 | 49.501 | 113.687 | 1.000 | FP-IS | FP |
| 77.243 | 44.755 | 111.738 | 1.000 | FP-O | FP |
| 16.927 | -5.295 | 41.154 | 0.932 | FP-SP | FP |
| -29.072 | -42.292 | -13.822 | 0.000 | IB-BP | IB |
| -0.042 | -21.683 | 25.422 | 0.488 | IB-C | IB |
| -43.095 | -52.698 | -32.252 | 0.000 | IB-FP | IB |
| 3.002 | -17.623 | 26.988 | 0.581 | IB-IF | IB |
| 1.855 | -16.756 | 22.958 | 0.546 | IB-IS | IB |
| 0.549 | -18.899 | 24.372 | 0.493 | IB-O | IB |
| -33.671 | -46.983 | -17.824 | 0.000 | IB-SP | IB |
| -30.693 | -44.192 | -14.954 | 0.000 | IF-BP | IF |
| -2.332 | -24.491 | 23.658 | 0.404 | IF-C | IF |
| -44.376 | -54.564 | -32.731 | 0.000 | IF-FP | IF |
| -1.722 | -21.252 | 21.393 | 0.419 | IF-IB | IF |
| -0.434 | -20.740 | 21.941 | 0.471 | IF-IS | IF |
| -1.732 | -22.867 | 22.053 | 0.424 | IF-O | IF |
| -35.176 | -49.134 | -19.493 | 0.000 | IF-SP | IF |
| -30.018 | -42.338 | -15.035 | 0.000 | IS-BP | IS |
| -1.383 | -22.512 | 24.345 | 0.428 | IS-C | IS |
| -43.846 | -53.203 | -33.111 | 0.000 | IS-FP | IS |
| -0.789 | -18.671 | 20.129 | 0.454 | IS-IB | IS |
| 1.646 | -17.993 | 26.166 | 0.529 | IS-IF | IS |
| -0.797 | -19.439 | 20.276 | 0.454 | IS-O | IS |
| -34.549 | -47.529 | -18.835 | 0.000 | IS-SP | IS |
| -29.027 | -42.451 | -13.847 | 0.001 | O-BP | O |
| -0.005 | -22.003 | 25.205 | 0.473 | O-C | O |
| -43.040 | -52.772 | -30.918 | 0.000 | O-FP | O |
| 0.657 | -19.596 | 23.302 | 0.507 | O-IB | O |
| 3.104 | -18.069 | 29.645 | 0.576 | O-IF | O |
| 1.958 | -16.858 | 24.129 | 0.546 | O-IS | O |
| -33.624 | -47.325 | -18.039 | 0.000 | O-SP | O |
| 7.633 | -12.741 | 31.724 | 0.735 | SP-BP | SP |
| 51.667 | 17.814 | 92.048 | 1.000 | SP-C | SP |
| -13.610 | -29.155 | 5.591 | 0.068 | SP-FP | SP |
| 52.655 | 21.690 | 88.620 | 1.000 | SP-IB | SP |
| 56.366 | 24.212 | 96.595 | 1.000 | SP-IF | SP |
| 54.654 | 23.206 | 90.581 | 1.000 | SP-IS | SP |

mu	li	ls	p_higher	category	var 52.604	22.009	89.843	1.000	SP-O	SP

text <-

of the swarm and the outside. diff_mu: average difference; diff_sd = SD of the difference; p: probability that the diference section i and section j is above zero; section: section used

to estimated the contrast. O = outside, IF = interior front,

IS = interior side, C = center, IB = interior border, BP = back perimeter, SP = side perimeter, FP = front perimeter'

knitr::kable(

'Contrast of prey biomass among the inside sections

lapply(1, FUN =

**function**(x) {

j <- do.call('rbind', p_prey[c('C', 'IB', 'IF', 'IS')]) indx <-

**function**(i) {

grepl(i, j$category)

})

j[!as.logical(apply(indx, 1, sum)), ]

})[[1]],

digits = 3, caption = text)

sapply(c('FP', 'SP', 'BP'), FUN =

Table S3: Contrast of prey biomass among the inside sections of the swarm and the outside. diff_mu: average difference; diff_sd = SD of the difference; p: probability that the diference section i and section j is above zero; section: section used to estimated the contrast. O = outside, IF = interior front, IS = interior side, C = center, IB = interior border, BP = back perimeter, SP = side perimeter, FP = front perimeter

| mu | li | ls | p_higher | category | var |
| --- | --- | --- | --- | --- | --- |
| 1.571 | -20.269 | 27.687 | 0.512 | C-IB | C |
| 4.015 | -19.132 | 32.434 | 0.596 | C-IF | C |
| 2.878 | -19.579 | 29.051 | 0.572 | C-IS | C |
| 1.502 | -20.131 | 28.210 | 0.527 | C-O | C |
| -0.042 | -21.683 | 25.422 | 0.488 | IB-C | IB |
| 3.002 | -17.623 | 26.988 | 0.581 | IB-IF | IB |
| 1.855 | -16.756 | 22.958 | 0.546 | IB-IS | IB |
| 0.549 | -18.899 | 24.372 | 0.493 | IB-O | IB |
| -2.332 | -24.491 | 23.658 | 0.404 | IF-C | IF |
| -1.722 | -21.252 | 21.393 | 0.419 | IF-IB | IF |
| -0.434 | -20.740 | 21.941 | 0.471 | IF-IS | IF |
| -1.732 | -22.867 | 22.053 | 0.424 | IF-O | IF |
| -1.383 | -22.512 | 24.345 | 0.428 | IS-C | IS |
| -0.789 | -18.671 | 20.129 | 0.454 | IS-IB | IS |
| 1.646 | -17.993 | 26.166 | 0.529 | IS-IF | IS |

mu	li	ls	p_higher	category	var

-0.797	-19.439	20.276	0.454	IS-O	IS

# S5 Merging prey and bird data

The most relevant task conducted in this section was merging the data sets of prey biomass and ant-followers space use. In those cases where we had missing prey biomass at the 𝑠𝑒𝑐𝑡𝑖𝑜𝑛_𝑖_ for a specific date and swarm, we used the *Model 1* to impute NA values by marginalizing 𝑑𝑎𝑡𝑒 and 𝑠𝑤𝑎𝑟𝑚′𝑠 𝑠𝑒𝑐𝑡𝑖𝑜𝑛, or 𝑠𝑤𝑎𝑟𝑚 𝑖𝑑𝑒𝑛𝑡𝑖𝑡𝑦 and 𝑠𝑤𝑎𝑟𝑚′𝑠 𝑠𝑒𝑐𝑡𝑖𝑜𝑛 parameters depending on the case. Through this method, we constructed a relational data set to fit the following models.

birds3$round_number <- as.numeric(birds3$round_number) prey$indx <- 1:nrow(prey)

extract_prey_posterior <-

**function**(date_ = 1,

swarm_ID_ = 1,

round_number_ = 1,

category_ = 1,

seed = 555) {

date_ <- birds3$date[date_]

swarm_ID_ <- birds3$swarm_ID[swarm_ID_] round_number_ <- birds3$round_number[round_number_] category_ <- birds3$categories[category_]

indx <- prey[prey$date == date_ &

prey$swarm_ID == swarm_ID_ & prey$round_number == round_number_ & prey$categories == category_, ]$indx

**if** (length(indx) == 0) {

warning('round number not found, averaging') indx2 <- prey[prey$date == date_ &

prey$swarm_ID == swarm_ID_ & prey$categories == category_, ]$indx

**if** (length(indx2) == 0) {

warning('round number not found, averaging')

indx3 <- prey[prey$categories == category_, ]$indx

posterior <- as.vector(ppcheck_prey[, indx3]) set.seed(seed)

posterior <- sample(posterior, 1e3) tibble(date = date_,

swarm_ID = swarm_ID_, round_number = round_number_, categories = category_,

mu_prey_biomas = median(posterior), ci1_prey_biomas = quantile(posterior, 0.025), ci2_prey_biomas = quantile(posterior, 0.975), PreBio_est = 'over_category')

} **else** {

posterior <- as.vector(ppcheck_prey[, indx2]) set.seed(seed)

posterior <- sample(posterior, 1e3) tibble(date = date_,

swarm_ID = swarm_ID_, round_number = round_number_, categories = category_,

mu_prey_biomas = median(posterior), ci1_prey_biomas = quantile(posterior, 0.025), ci2_prey_biomas = quantile(posterior, 0.975), PreBio_est = 'over_rounds')

}

} **else** {

warning('round number found') indx

posterior <- as.vector(ppcheck_prey[, indx]) set.seed(seed)

posterior <- sample(posterior, 1e3) tibble(date = date_,

swarm_ID = swarm_ID_, round_number = round_number_, categories = category_,

mu_prey_biomas = median(posterior), ci1_prey_biomas = quantile(posterior, 0.025), ci2_prey_biomas = quantile(posterior, 0.975), PreBio_est = 'found')

}

}

prey_bird <- lapply(1:nrow(birds3), FUN =

**function**(x) {

extract_prey_posterior(x, x, x, x, seed = x)

})

prey_bird <- do.call('rbind', prey_bird) qua_cat <-

full_join(unique(prey_bird[, c("date", "swarm_ID", "categories",

"round_number")]), unique(prey[, c("date", "swarm_ID", "categories",

"round_number", "type")]), by = c('date', 'swarm_ID', 'categories', 'round_number'))

qua_cat <- split(qua_cat, list(qua_cat$date,

qua_cat$swarm_ID, qua_cat$categories, qua_cat$round_number))

qua_cat <- qua_cat[!(unlist(lapply(qua_cat, nrow)) == 0)]

qua_cat1 <- lapply(qua_cat[unlist(lapply(qua_cat, nrow), use.names = F) == 2], FUN =

**function**(x) { x$type <- 'avg' x[1, ]

})

qua_cat1 <-

rbind(do.call('rbind', qua_cat[unlist(lapply(qua_cat, nrow), use.names = F) == 1]), do.call('rbind', qua_cat1))

birds4 <- as_tibble(cbind(birds3, prey_bird[, -c(1:4)])) qua_cat1$type[which(is.na(qua_cat1$type))] <- 'avg' qua_cat1$samp_eff <- ifelse(qua_cat1$type == 'quadrant', 0.5,

ifelse(qua_cat1$type == 'avg', mean(c(1, 0.5)), 1))

qua_cat1$samp_eff_std <- qua_cat1$samp_eff / mean(qua_cat1$samp_eff)

birds4 <- full_join(birds4, qua_cat1[, -c(5,6)],

by = c('date', 'swarm_ID', 'categories', 'round_number'))

spp_names <- read_xlsx('alpha_code_to_scientific_name.xlsx', sheet = 1, col_names = T) spp_names <- spp_names[!is.na(spp_names$alpha_code), ]

spp_names <- spp_names[spp_names$alpha_code %in% unique(birds4$Bird_Code), ]

sites <- read_xlsx('2023_General_Data.xlsx', sheet = 1)

New names:

- `UTM Easting` -> `UTM Easting...6`
- `UTM Easting` -> `UTM Easting...7`

sites$date <- as.Date(sites %$% paste('2023', Month, Day, sep = '-'),

format = '%Y-%b-%d')

sites <- sites[, c('date', "Antswarm ID", "Site")] colnames(sites) <- c('date', 'swarm_ID', 'site')

birds4 <- full_join(birds4, sites, by = c('date', 'swarm_ID'))

birds4 <- na.omit(birds4)

# S6 Model 2: effect of prey biomass and swarm area on ant-followers space use (assemblage level)

This model assess the variation of prey biomass across swarm sections. Below, we provide the code for data formatting, model in mathematical notation, Stan code, fitting procedure and model diagnostics.

## S6.1 Data preparation

dat_generative2 <-

**function**(x) { x

})

**for** (i **in** 1:5) dat_generative2[[i]] <-as.numeric(as.factor(as.character(dat_generative2[[i]])))

dat_generative2$N <- length(dat_generative2$date) dat_generative2$N_days <- max(dat_generative2$date) dat_generative2$N_site <- max(dat_generative2$site)

lapply(birds4[, c("date", "swarm_ID", "site", "round_number", "categories", "swarm_area", "mu_prey_biomas",

"obligate", "facultative", "occasional", "total", "samp_eff_std")],

dat_generative2$N_swarm <- max(dat_generative2$swarm_ID) dat_generative2$N_round_number <- max(dat_generative2$round_number) dat_generative2$N_categories <- max(dat_generative2$categories) dat_generative2$dist_day <- dist_day_bird

dat_generative2$z_swarm_area <- as.vector(scale(dat_generative2$swarm_area)) dat_generative2$z_prey_biomass <- as.vector(scale(dat_generative2$mu_prey_biomas))

## S6.2 Mathematical notation

The following Bayesian hierarchical model asses the interrelated processes affecting the space use by the ant-followers assemblage (i.e. 𝑠𝑤𝑎𝑟𝑚 → 𝑝𝑟𝑒𝑦 𝑏𝑖𝑜𝑚𝑎𝑠𝑠; 𝑠𝑤𝑎𝑟𝑚 → 𝑏𝑖𝑟𝑑𝑠; 𝑠𝑤𝑎𝑟𝑚 →

𝑝𝑟𝑒𝑦 𝑏𝑖𝑜𝑚𝑎𝑠𝑠 → 𝑏𝑖𝑟𝑑𝑠; 𝑝𝑟𝑒𝑦 𝑏𝑖𝑜𝑚𝑎𝑠𝑠 → 𝑏𝑖𝑟𝑑𝑠. Figure below).We used a 𝑆𝑡𝑢𝑑𝑒𝑛𝑡-𝑇 likelihood for modelling z-scores of swarm area and prey biomass, and employed a 𝐵𝑒𝑟𝑛𝑜𝑢𝑙𝑙𝑖 likelihood to model the probability of observing the ant-followers.In all models we used Gaussian processes to estimate 𝜃 parameters and account for temporal correlation. Other grouping factors were estimated using partial polling and a non-centered parametrization.

𝑆𝑤𝑎𝑟𝑚 𝑎𝑟𝑒𝑎𝑖 ∼ 𝑆𝑡𝑢𝑑𝑒𝑛𝑡𝑇(𝜈 = 2, 𝜇2𝑖, 𝜎2)

𝜇1𝑖 = 𝛼3[𝑠𝑤𝑎𝑟𝑚 𝑖] + 𝜓3[𝑠𝑖𝑡𝑒 𝑖]^+^

𝜏3[𝑟𝑜𝑢𝑛𝑑 𝑖] + 𝜃3[𝑑𝑎𝑡𝑒 𝑖]

𝑃𝑟𝑒𝑦 𝑏𝑖𝑜𝑚𝑎𝑠𝑠𝑖 ∼ 𝑆𝑡𝑢𝑑𝑒𝑛𝑡𝑇(𝜈 = 2, 𝜇1𝑖, 𝜎1)

𝜇1𝑖 = 𝛼2[𝑠𝑤𝑎𝑟𝑚 𝑖] + 𝜓2[𝑠𝑖𝑡𝑒 𝑖] + 𝛾[𝑠𝑒𝑐𝑡𝑖𝑜𝑛 𝑖]^+^

𝜏2[𝑟𝑜𝑢𝑛𝑑 𝑖] + 𝜃2[𝑑𝑎𝑡𝑒 𝑖]

𝛽3[𝑠𝑒𝑐𝑡𝑖𝑜𝑛 𝑖] × 𝑠𝑤𝑎𝑟𝑚 𝑎𝑟𝑒𝑎𝑖 + 𝑠𝑎𝑚𝑝𝑙𝑖𝑛𝑔 𝑒𝑓𝑓𝑜𝑟𝑡𝑖

𝐴𝑛𝑡 𝑓𝑜𝑙𝑙𝑜𝑤𝑒𝑟𝑖 ∼ 𝐵𝑒𝑟𝑛𝑜𝑢𝑙𝑙𝑖(𝑝𝑖)

𝑙𝑜𝑔𝑖𝑡(𝑝𝑖) = 𝛼1[𝑠𝑒𝑐𝑡𝑖𝑜𝑛 𝑖] + 𝜓1[𝑠𝑖𝑡𝑒 𝑖] + 𝛿𝑠𝑤𝑎𝑟𝑚 𝑖

𝜏1[𝑟𝑜𝑢𝑛𝑑 𝑖] + 𝜃1[𝑑𝑎𝑡𝑒 𝑖]^+^

𝛽1 × 𝑠𝑤𝑎𝑟𝑚 𝑎𝑟𝑒𝑎𝑖+

𝛽2 × 𝑝𝑟𝑒𝑦 𝑏𝑖𝑜𝑚𝑎𝑠𝑠𝑖

𝛼1...𝛼3 = 𝜇𝛼1...𝛼3 + 𝑧𝛼1...𝛼3 × 𝜎𝛼1...𝛼3

𝜇𝛼_1_...𝛼_3_ ∼ 𝑁𝑜𝑟𝑚𝑎𝑙(0, 1)

𝑧𝛼_1_...𝛼_3_ ∼ 𝑁𝑜𝑟𝑚𝑎𝑙(0, 1)

𝜎𝛼_1_...𝛼_3_ ∼ 𝐸𝑥𝑝𝑜𝑛𝑒𝑛𝑡𝑖𝑎𝑙(1)

𝜏1...𝜏3 = 𝜇𝜏1...𝜏3 + 𝑧𝜏1...𝜏3 × 𝜎𝜏1...𝜏3

𝜇𝜏_1_...𝜏_3_ ∼ 𝑁𝑜𝑟𝑚𝑎𝑙(0, 1)

𝑧𝜏_1_...𝜏_3_ ∼ 𝑁𝑜𝑟𝑚𝑎𝑙(0, 1)

𝜎𝜏_1_...𝜏_3_ ∼ 𝐸𝑥𝑝𝑜𝑛𝑒𝑛𝑡𝑖𝑎𝑙(1)

𝜓1...𝜓3 = 𝜇𝜓1...𝜓3 + 𝑧𝜓1...𝜓3 × 𝜎𝜓1...𝜓3

𝜇𝜓_1_...𝜓_3_ ∼ 𝑁𝑜𝑟𝑚𝑎𝑙(0, 1)

𝑧𝜓_1_...𝜓_3_ ∼ 𝑁𝑜𝑟𝑚𝑎𝑙(0, 1)

𝜎𝜓_1_...𝜓_3_ ∼ 𝐸𝑥𝑝𝑜𝑛𝑒𝑛𝑡𝑖𝑎𝑙(1)

𝛿𝑠𝑤𝑎𝑟𝑚 𝑖 = 𝜇𝛿 + 𝑧𝛿 × 𝜎𝛿

𝜇𝛿 ∼ 𝑁𝑜𝑟𝑚𝑎𝑙(0, 1)

𝑧𝛿 ∼ 𝑁𝑜𝑟𝑚𝑎𝑙(0, 1)

𝜎𝛿 ∼ 𝐸𝑥𝑝𝑜𝑛𝑒𝑛𝑡𝑖𝑎𝑙(1)

𝛾𝑠𝑒𝑐𝑡𝑖𝑜𝑛 𝑖 = 𝜇𝛾 + 𝑧𝛾 × 𝜎𝛾

𝜇𝛾 ∼ 𝑁𝑜𝑟𝑚𝑎𝑙(0, 0.5)

𝑧𝛾 ∼ 𝑁𝑜𝑟𝑚𝑎𝑙(0, 0.5)

𝜎𝛾 ∼ 𝐸𝑥𝑝𝑜𝑛𝑒𝑛𝑡𝑖𝑎𝑙(1)

⎛ 𝜃1𝑑𝑎𝑦 1 ⎞

⎛ 𝜃3𝑑𝑎𝑦 1 ⎞

⎛	^0^	⎞⎟

⎜ 𝜃1𝑑𝑎𝑦 2

⎟ , ..., ⎜

𝜃3𝑑𝑎𝑦 2

⎟ ∼ 𝑀𝑉 𝑁𝑜𝑟𝑚𝑎𝑙 ⎜ ⎡⎢ 0 ⎤⎥ 𝐾𝜃1...𝜃3 ⎟

⎜	...	⎟

⎜	...	⎟

⎢...⎥

⎝ 𝜃1𝑑𝑎𝑦 𝑛 ⎠

𝜃1...𝜃3

⎝ 𝜃3𝑑𝑎𝑦 𝑛 ⎠

⎝ ⎣ 0 ⎦	⎠

𝐾𝜃1...𝜃3 = 𝜂2

𝜃1...𝜃3

𝑒𝑥𝑝(−𝜌^2^

2

𝑑𝑎𝑦𝑠 𝑖𝑗

𝐷

) + 𝛿𝜃1...𝜃3 𝑖𝑗 × 0.01

\## Stan code

𝜂𝜃1...𝜃3 ∼ 𝐸𝑥𝑝𝑜𝑛𝑒𝑛𝑡𝑖𝑎𝑙(3)

𝜌𝜃1...𝜃3 ∼ 𝐸𝑥𝑝𝑜𝑛𝑒𝑛𝑡𝑖𝑎𝑙(3)

𝛽1, ..., 𝛽3 ∼ 𝑁𝑜𝑟𝑚𝑎𝑙(0, 1)

cat(file = 'total.stan',

"

functions {

matrix cov_GPL2(matrix x,

real eta, real rho, real delta) {

int N = dims(x)[1];

matrix[N, N] k;

for (i in 1:(N-1)) { k[i, i] = eta + delta; for (j in (i + 1):N) {

k[i, j] = eta * exp(-rho * square(x[i, j])); k[j, i] = k[i, j];

}

}

}

data {

int N;

int N_swarm; int N_site;

k[N, N] = eta + delta; return k;

}

int N_round_number; int N_days;

int N_categories;

matrix[N_days, N_days] dist_day; array[N] int date;

array[N] int swarm_ID; array[N] int site; array[N] int round_number; array[N] int categories; vector[N] z_prey_biomass; vector[N] z_swarm_area; vector[N] samp_eff_std; array[N] int total;

}

parameters {

// ant swarm

vector[N_swarm] z_alpha_swarm; real mu_alpha_swarm;

real<lower = 0> sigma_alpha_swarm;

vector[N_site] z_psi_swarm; real mu_psi_swarm;

real<lower = 0> sigma_psi_swarm;

vector[N_round_number] z_tau_swarm; real mu_tau_swarm;

real<lower = 0> sigma_tau_swarm;

vector[N_days] z_theta_swarm; real<lower = 0> eta_theta_swarm; real<lower = 0> rho_theta_swarm;

real<lower = 0> sigma_swarm;

// prey biomass

vector[N_swarm] z_alpha_prey; real mu_alpha_prey;

real<lower = 0> sigma_alpha_prey;

vector[N_site] z_psi_prey; real mu_psi_prey;

real<lower = 0> sigma_psi_prey;

vector[N_round_number] z_tau_prey; real mu_tau_prey;

real<lower = 0> sigma_tau_prey;

vector[N_categories] z_gamma_prey; real mu_gamma_prey;

real<lower = 0> sigma_gamma_prey;

vector[N_days] z_theta_prey; real<lower = 0> eta_theta_prey; real<lower = 0> rho_theta_prey;

vector[N_categories] beta_swarm_prey; real<lower = 0> sigma_prey;

// Obligate

real beta_swarmO; real beta_preyO;

vector[N_categories] z_alphaO; real mu_alphaO;

real<lower = 0> sigma_alphaO;

vector[N_days] z_thetaO; real<lower = 0> eta_thetaO; real<lower = 0> rho_thetaO;

vector[N_round_number] z_tauO; real mu_tauO;

real<lower = 0> sigma_tauO;

vector[N_site] z_psiO; real mu_psiO;

real<lower = 0> sigma_psiO;

vector[N_swarm] z_deltaO; real mu_deltaO;

real<lower = 0> sigma_deltaO;

}

transformed parameters {

// ant swarm

vector[N_days] theta_swarm; matrix[N_days, N_days] sigma_theta_swarm;

matrix[N_days, N_days] L_sigma_theta_swarm; sigma_theta_swarm = cov_GPL2(dist_day,

eta_theta_swarm, rho_theta_swarm, 0.01);

L_sigma_theta_swarm = cholesky_decompose(sigma_theta_swarm); theta_swarm = L_sigma_theta_swarm * z_theta_swarm;

vector[N_swarm] alpha_swarm;

alpha_swarm = mu_alpha_swarm + z_alpha_swarm * sigma_alpha_swarm;

vector[N_site] psi_swarm;

psi_swarm = mu_psi_swarm + z_psi_swarm * sigma_psi_swarm;

vector[N_round_number] tau_swarm;

tau_swarm = mu_tau_swarm + z_tau_swarm * sigma_tau_swarm;

// prey biomass vector[N_days] theta_prey;

matrix[N_days, N_days] sigma_theta_prey;

matrix[N_days, N_days] L_sigma_theta_prey; sigma_theta_prey = cov_GPL2(dist_day,

eta_theta_prey, rho_theta_prey, 0.01);

L_sigma_theta_prey = cholesky_decompose(sigma_theta_prey); theta_prey = L_sigma_theta_prey * z_theta_prey;

vector[N_swarm] alpha_prey;

alpha_prey = mu_alpha_prey + z_alpha_prey * sigma_alpha_prey;

vector[N_site] psi_prey;

psi_prey = mu_psi_prey + z_psi_prey * sigma_psi_prey;

vector[N_round_number] tau_prey;

tau_prey = mu_tau_prey + z_tau_prey * sigma_tau_prey;

vector[N_round_number] gamma_prey;

gamma_prey = mu_tau_prey + z_gamma_prey * sigma_gamma_prey;

// Obligate vector[N_days] thetaO;

matrix[N_days, N_days] sigma_thetaO;

matrix[N_days, N_days] L_sigma_thetaO;

sigma_thetaO = cov_GPL2(dist_day, eta_thetaO, rho_thetaO, 0.01); L_sigma_thetaO = cholesky_decompose(sigma_thetaO);

thetaO = L_sigma_thetaO * z_thetaO;

vector[N_round_number] tauO;

tauO = mu_tauO + z_tauO * sigma_tauO;

vector[N_site] psiO;

psiO = mu_psiO + z_psiO * sigma_psiO;

vector[N_swarm] deltaO;

deltaO = mu_deltaO + z_deltaO * sigma_deltaO;

vector[N_categories] alphaO;

alphaO = mu_alphaO + z_alphaO * sigma_alphaO;

}

model {

// ant swarm

z_alpha_swarm ~ normal(0, 1); mu_alpha_swarm ~ normal(0, 1); sigma_alpha_swarm ~ exponential(1);

z_psi_swarm ~ normal(0, 1); mu_psi_swarm ~ normal(0, 1); sigma_psi_swarm ~ exponential(1);

z_theta_swarm ~ normal(0, 1); eta_theta_swarm ~ exponential(3); rho_theta_swarm ~ exponential(3);

z_tau_swarm ~ normal(0, 1);

mu_tau_swarm ~ normal(0, 1); sigma_tau_swarm ~ exponential(1);

sigma_swarm ~ exponential(1);

// prey biomass

beta_swarm_prey ~ normal(0, 1);

z_alpha_prey ~ normal(0, 1); mu_alpha_prey ~ normal(0, 1); sigma_alpha_prey ~ exponential(1);

z_psi_prey ~ normal(0, 1); mu_psi_prey ~ normal(0, 1); sigma_psi_prey ~ exponential(1);

z_theta_prey ~ normal(0, 1); eta_theta_prey ~ exponential(3); rho_theta_prey ~ exponential(3);

z_tau_prey ~ normal(0, 0.5); mu_tau_prey ~ normal(0, 0.5); sigma_tau_prey ~ exponential(1);

z_gamma_prey ~ normal(0, 0.5); mu_gamma_prey ~ normal(0, 0.5); sigma_gamma_prey ~ exponential(1);

sigma_prey ~ exponential(1);

// total

beta_swarmO ~ normal(0, 1.5); beta_preyO ~ normal(0, 1.5);

z_alphaO ~ normal(0, 1); mu_alphaO ~ normal(0, 1); sigma_alphaO ~ exponential(1);

z_thetaO ~ normal(0, 1); eta_thetaO ~ exponential(3); rho_thetaO ~ exponential(3);

z_tauO ~ normal(0, 1); mu_tauO ~ normal(0, 1); sigma_tauO ~ exponential(1);

z_psiO ~ normal(0, 1); mu_psiO ~ normal(0, 1); sigma_psiO ~ exponential(1);

z_deltaO ~ normal(0, 1); mu_psiO ~ normal(0, 1); sigma_psiO ~ exponential(1);

for (i in 1:N) {

z_swarm_area[i] ~ student_t(2, alpha_swarm[swarm_ID[i]] +

psi_swarm[site[i]] + theta_swarm[date[i]] + tau_swarm[round_number[i]], sigma_swarm);

}

for (i in 1:N) {

z_prey_biomass[i] ~ student_t(2, alpha_prey[swarm_ID[i]] +

gamma_prey[categories[i]] +

}

for (i in 1:N) {

beta_swarm_prey[categories[i]]*z_swarm_area[i] + psi_prey[site[i]] +

theta_prey[date[i]] + tau_prey[round_number[i]] + samp_eff_std[i], sigma_prey);

}

total[i] ~ bernoulli(inv_logit(alphaO[categories[i]] +

beta_swarmO * z_swarm_area[i] + beta_preyO * z_prey_biomass[i] + psiO[site[i]] + deltaO[swarm_ID[i]] +

thetaO[date[i]] + tauO[round_number[i]]));

}

generated quantities { vector[N] muO; array[N] int ppcheckO; vector[N] mu_swarm;

array[N] real ppcheck_swarm; vector[N] mu_prey;

array[N] real ppcheck_prey;

for (i in 1:N) {

mu_swarm[i] = alpha_swarm[swarm_ID[i]] +

psi_swarm[site[i]] + theta_swarm[date[i]] + tau_swarm[round_number[i]];

}

ppcheck_swarm = student_t_rng(2, mu_swarm, sigma_swarm); for (i in 1:N) {

mu_prey[i] = alpha_prey[swarm_ID[i]] +

gamma_prey[categories[i]] + beta_swarm_prey[categories[i]]*z_swarm_area[i] + psi_prey[site[i]] +

theta_prey[date[i]] + tau_prey[round_number[i]] + samp_eff_std[i];

}

ppcheck_prey = student_t_rng(2, mu_prey, sigma_prey); for (i in 1:N) {

muO[i] = alphaO[categories[i]] +

beta_swarmO * z_swarm_area[i] + beta_preyO * z_prey_biomass[i] + psiO[site[i]] + deltaO[swarm_ID[i]] + thetaO[date[i]] + tauO[round_number[i]];

muO[i] = inv_logit(muO[i]);

}

ppcheckO = bernoulli_rng(muO);

} ")

## S6.3 Fitting procedure

file <- paste(getwd(), '/total.stan', sep ='') fit_total <- cmdstan_model(file, compile = T)

mod_total <-fit_total$sample(

data = dat_generative2, chains = 3,

iter_warmup = 500, iter_sampling = 4e3, parallel_chains = 3,

thin = 3,

refresh = 50,

seed = 123

)

## S6.4 Summary

pars <- c('beta_swarm', 'beta_prey', 'alpha', 'theta', 'tau', 'psi', 'delta')

pars2 <- c('alpha', 'theta', 'tau', 'psi', 'sigma') pars <- c(paste(pars2, '_swarm', sep = ''),

paste(pars2, '_prey', sep = ''),

paste(pars, 'O', sep = ''), 'gamma_prey', 'beta_swarm_prey')

summary_mod_total <- mod_total$summary(pars) knitr::kable(

rbind(summary_mod_total[grep('beta_swarm', summary_mod_total$variable), ],

summary_mod_total[grep('beta_prey', summary_mod_total$variable), ], summary_mod_total[grep('beta_swarm_prey', summary_mod_total$variable), ], summary_mod_total[grep('alpha', summary_mod_total$variable), ]),

digits = 2,

caption = 'Estimated coefficients and sampling diagnostics of the model assessing the interrelated processes

)

Table S4: Estimated coeﬀicients and sampling diagnostics of the model assessing the interre-lated processes affecting the space use by the ant-followers assemblage

| variable | mean | median | sd | mad | q5 | q95 | rhat | ess_bulk | ess_tail |
| --- | --- | --- | --- | --- | --- | --- | --- | --- | --- |
| beta_swarmO | 0.53 | 0.53 | 0.10 | 0.10 | 0.37 | 0.70 | 1 | 3903.59 | 3695.76 |
| beta_swarm_prey[1]-0.02 | | -0.02 | 0.03 | 0.03 | -0.06 | 0.03 | 1 | 4386.59 | 3604.42 |
| beta_swarm_prey[2]-0.07 | | -0.07 | 0.03 | 0.03 | -0.12 | -0.03 | 1 | 4694.55 | 3931.05 |
| beta_swarm_prey[3] 0.04 | | 0.04 | 0.02 | 0.02 | 0.01 | 0.07 | 1 | 4696.41 | 3608.73 |
| beta_swarm_prey[4]-0.04 | | -0.04 | 0.02 | 0.02 | -0.07 | 0.00 | 1 | 4257.94 | 3775.16 |
| beta_swarm_prey[5]-0.12 | | -0.12 | 0.03 | 0.03 | -0.17 | -0.07 | 1 | 4405.63 | 3971.79 |
| beta_swarm_prey[6]-0.01 | | -0.01 | 0.02 | 0.02 | -0.05 | 0.03 | 1 | 4583.39 | 3611.39 |
| beta_swarm_prey[7] 0.04 | | 0.04 | 0.03 | 0.03 | -0.01 | 0.08 | 1 | 4564.76 | 3670.36 |

| variable | mean | median | sd | mad | q5 | q95 | rhat | ess_bulk | ess_tail |
| --- | --- | --- | --- | --- | --- | --- | --- | --- | --- |
| beta_swarm_prey[8] 0.02 | | 0.02 | 0.04 | 0.04 | -0.04 | 0.09 | 1 | 4622.87 | 3922.88 |
| beta_preyO 0.16 | | 0.16 | 0.15 | 0.15 | -0.10 | 0.40 | 1 | 3794.31 | 3365.00 |
| beta_swarm_prey[1]-0.02 | | -0.02 | 0.03 | 0.03 | -0.06 | 0.03 | 1 | 4386.59 | 3604.42 |
| beta_swarm_prey[2]-0.07 | | -0.07 | 0.03 | 0.03 | -0.12 | -0.03 | 1 | 4694.55 | 3931.05 |
| beta_swarm_prey[3] 0.04 | | 0.04 | 0.02 | 0.02 | 0.01 | 0.07 | 1 | 4696.41 | 3608.73 |
| beta_swarm_prey[4]-0.04 | | -0.04 | 0.02 | 0.02 | -0.07 | 0.00 | 1 | 4257.94 | 3775.16 |
| beta_swarm_prey[5]-0.12 | | -0.12 | 0.03 | 0.03 | -0.17 | -0.07 | 1 | 4405.63 | 3971.79 |
| beta_swarm_prey[6]-0.01 | | -0.01 | 0.02 | 0.02 | -0.05 | 0.03 | 1 | 4583.39 | 3611.39 |
| beta_swarm_prey[7] 0.04 | | 0.04 | 0.03 | 0.03 | -0.01 | 0.08 | 1 | 4564.76 | 3670.36 |
| beta_swarm_prey[8] 0.02 | | 0.02 | 0.04 | 0.04 | -0.04 | 0.09 | 1 | 4622.87 | 3922.88 |
| alpha_swarm[1] 0.20 | | 0.21 | 0.90 | 0.89 | -1.28 | 1.71 | 1 | 4190.60 | 3811.20 |
| alpha_swarm[2] 0.45 | | 0.46 | 0.84 | 0.84 | -0.92 | 1.85 | 1 | 4182.92 | 3767.64 |
| alpha_swarm[3] -0.13 | | -0.13 | 0.89 | 0.88 | -1.61 | 1.36 | 1 | 4222.30 | 3919.36 |
| alpha_swarm[4] -0.12 | | -0.13 | 0.89 | 0.88 | -1.56 | 1.32 | 1 | 4114.45 | 3431.01 |
| alpha_swarm[5] -0.27 | | -0.26 | 0.89 | 0.90 | -1.71 | 1.20 | 1 | 4014.13 | 3575.96 |
| alpha_swarm[6] -0.05 | | -0.06 | 0.89 | 0.89 | -1.50 | 1.45 | 1 | 4182.91 | 3642.88 |
| alpha_swarm[7] -0.21 | | -0.22 | 0.89 | 0.88 | -1.68 | 1.28 | 1 | 4130.00 | 3833.88 |
| alpha_swarm[8] 0.09 | | 0.09 | 0.88 | 0.89 | -1.39 | 1.54 | 1 | 4052.07 | 3741.29 |
| alpha_swarm[9] -0.11 | | -0.13 | 0.90 | 0.90 | -1.61 | 1.40 | 1 | 4118.97 | 3702.41 |
| alpha_swarm[10] 0.03 | | 0.02 | 0.89 | 0.89 | -1.45 | 1.50 | 1 | 4089.27 | 3517.41 |
| alpha_swarm[11] -0.40 | | -0.39 | 0.84 | 0.85 | -1.79 | 1.01 | 1 | 4168.51 | 3841.20 |
| alpha_swarm[12] -0.14 | | -0.15 | 0.90 | 0.90 | -1.63 | 1.33 | 1 | 4152.97 | 3543.61 |
| alpha_swarm[13] 0.09 | | 0.08 | 0.87 | 0.87 | -1.33 | 1.53 | 1 | 4004.10 | 3413.81 |
| alpha_swarm[14] 0.10 | | 0.10 | 0.88 | 0.86 | -1.36 | 1.58 | 1 | 4110.52 | 3815.95 |
| alpha_prey[1] -0.29 | | -0.29 | 0.85 | 0.84 | -1.67 | 1.11 | 1 | 4303.83 | 3490.79 |
| alpha_prey[2] -0.36 | | -0.36 | 0.85 | 0.86 | -1.76 | 1.04 | 1 | 4211.05 | 3356.18 |
| alpha_prey[3] -0.32 | | -0.32 | 0.85 | 0.84 | -1.74 | 1.10 | 1 | 4312.80 | 3628.37 |
| alpha_prey[4] -0.32 | | -0.31 | 0.86 | 0.85 | -1.74 | 1.10 | 1 | 4366.78 | 3490.39 |
| alpha_prey[5] -0.46 | | -0.45 | 0.86 | 0.85 | -1.89 | 0.97 | 1 | 4223.44 | 3576.82 |
| alpha_prey[6] -0.15 | | -0.16 | 0.88 | 0.87 | -1.58 | 1.31 | 1 | 4136.09 | 3627.58 |
| alpha_prey[7] -0.36 | | -0.35 | 0.85 | 0.85 | -1.75 | 1.04 | 1 | 4386.39 | 3710.22 |
| alpha_prey[8] -0.36 | | -0.34 | 0.86 | 0.86 | -1.77 | 1.04 | 1 | 4312.30 | 3624.85 |
| alpha_prey[9] -0.43 | | -0.43 | 0.86 | 0.86 | -1.84 | 0.99 | 1 | 4351.20 | 3815.11 |
| alpha_prey[10] -0.45 | | -0.43 | 0.86 | 0.86 | -1.88 | 0.97 | 1 | 4331.74 | 3723.00 |
| alpha_prey[11] -0.33 | | -0.33 | 0.85 | 0.85 | -1.73 | 1.07 | 1 | 4238.32 | 3449.86 |
| alpha_prey[12] -0.39 | | -0.39 | 0.86 | 0.86 | -1.79 | 1.01 | 1 | 4259.90 | 3441.81 |
| alpha_prey[13] -0.53 | | -0.51 | 0.85 | 0.85 | -1.92 | 0.86 | 1 | 4334.42 | 3265.18 |
| alpha_prey[14] -0.33 | | -0.33 | 0.85 | 0.86 | -1.71 | 1.08 | 1 | 4322.38 | 3604.63 |
| alphaO[1] 0.41 | | 0.41 | 1.06 | 1.08 | -1.29 | 2.18 | 1 | 4205.21 | 3613.09 |
| alphaO[2] -0.56 | | -0.56 | 1.06 | 1.07 | -2.30 | 1.19 | 1 | 4007.46 | 3576.06 |
| alphaO[3] 1.18 | | 1.14 | 1.08 | 1.08 | -0.55 | 3.02 | 1 | 3998.92 | 3551.20 |

Rhat

1.000

1.006

| variable | mean | median | sd | mad | q5 | q95 | rhat | ess_bulk | ess_tail |
| --- | --- | --- | --- | --- | --- | --- | --- | --- | --- |
| alphaO[4] | -0.09 | -0.08 | 1.05 | 1.06 | -1.81 | 1.65 | 1 | 4057.19 | 3823.76 |
| alphaO[5] | 0.22 | 0.23 | 1.05 | 1.08 | -1.53 | 1.97 | 1 | 4187.31 | 3893.72 |
| alphaO[6] | -0.09 | -0.10 | 1.05 | 1.05 | -1.84 | 1.67 | 1 | 4074.70 | 3611.06 |
| alphaO[7] | -0.97 | -0.98 | 1.06 | 1.07 | -2.73 | 0.80 | 1 | 3944.86 | 3651.98 |
| alphaO[8] | 0.01 | 0.00 | 1.06 | 1.07 | -1.71 | 1.80 | 1 | 4092.63 | 3796.89 |

## S6.5 Sampling diagnostics

mod_diagnostics(mod_total, summary_mod_total)

0	1000	2000	3000	4000	5000

ESS parameters (tail and bulk)

Figure S8: Bulk and tail effective sampling size (ESS) of the model assessing the interrelated processes affecting the space use by the ant-followers assemblage

ppcheck_total <- mod_total$draws('ppcheckO', format = 'matrix')

par(mfrow = c(2, 2), mar = c(4, 4, 1, 1)) plot(density(dat_generative2$total), main = '', xlab = 'Ant-followers',

**for** (i **in** 1:100) lines(density(ppcheck_total[i, ]), lwd = 0.1) lines(density(dat_generative2$total), col = 'red', lwd = 2)

ylim = c(0, 3), lwd = 0)

Density

0.0

1.5

3.0

Density

0.0

0.4

0.8

ppcheck_swarm <- mod_total$draws('ppcheck_swarm', format = 'matrix') plot(density(dat_generative2$z_swarm_area), ylim = c(0, 0.8),

**for** (i **in** 1:100) lines(density(ppcheck_swarm[i, ]), lwd = 0.1) lines(density(dat_generative2$z_swarm_area), col = 'red', lwd = 3)

ppcheck_prey <- mod_total$draws('ppcheck_prey', format = 'matrix') plot(density(dat_generative2$z_prey_biomass), ylim = c(0, 0.8),

xlab = 'Swarm area', main = '')

**for** (i **in** 1:500) lines(density(ppcheck_prey[i, ]), lwd = 0.1) lines(density(dat_generative2$z_prey_biomass), col = 'red', lwd = 3) par(mfrow = c(1, 1))

xlab = 'Prey biomass', main = '')

0.0	0.5	1.0	−2	0	2	4

Ant−followers	Swarm area

Density

0.0

0.4

0.8

−2	0	2	4	6

Prey biomass

Figure S9: Posterior predictive checks of the model assessing the interrelated processes affect-ing the space use by the ant-followers assemblage

## S6.6 Posterior distributions

In this section we extract the posterior distributions and used the structure of the model to plot and estimate conditional effects

post_total <- mod_total$draws(pars, format = 'df')

post_total <-

lapply(pars, FUN =

**function**(x) {

post_total[grep(x, names(post_total))]

})

Warning: Dropping 'draws_df' class as required metadata was removed. Warning: Dropping 'draws_df' class as required metadata was removed. Warning: Dropping 'draws_df' class as required metadata was removed. Warning: Dropping 'draws_df' class as required metadata was removed. Warning: Dropping 'draws_df' class as required metadata was removed. Warning: Dropping 'draws_df' class as required metadata was removed. Warning: Dropping 'draws_df' class as required metadata was removed. Warning: Dropping 'draws_df' class as required metadata was removed. Warning: Dropping 'draws_df' class as required metadata was removed. Warning: Dropping 'draws_df' class as required metadata was removed. Warning: Dropping 'draws_df' class as required metadata was removed. Warning: Dropping 'draws_df' class as required metadata was removed. Warning: Dropping 'draws_df' class as required metadata was removed. Warning: Dropping 'draws_df' class as required metadata was removed. Warning: Dropping 'draws_df' class as required metadata was removed. Warning: Dropping 'draws_df' class as required metadata was removed. Warning: Dropping 'draws_df' class as required metadata was removed. Warning: Dropping 'draws_df' class as required metadata was removed. Warning: Dropping 'draws_df' class as required metadata was removed.

names(post_total) <- pars

group_codes <- lapply(dat_generative2[1:5], FUN =

**function**(x) {

x <- unique(x)

x <- as.factor(as.character(sort(x))) tibble(label = x,

code = as.numeric(x))

})

### S6.6.1 Effect of swarm on prey biomass

swarm_effect_prey <-

lapply(1, FUN =

**function**(x) {

x_seq <- seq(min(dat_generative2$z_swarm_area), max(dat_generative2$z_swarm_area), length.out = 100)

z <-

lapply(seq_along(post_total$beta_swarm_prey), FUN =

**function**(k) {

indx <- which(dat_generative2$categories == k) a <- unique(dat_generative2$swarm_ID[indx])

p <- unique(dat_generative2$site[indx]) t <- unique(dat_generative2$date[indx])

ta <- unique(dat_generative2$round_number[indx])

l <- which(group_codes$categories$code == k) l <- group_codes$categories$label[l]

samp_eff <- dat_generative2$samp_eff_std[indx]

m <-

sapply(x_seq, FUN =

**function**(i) {

with(post_total,

{

apply(alpha_prey[, a], 1, mean) + gamma_prey[[k]] + beta_swarm_prey[[k]] * i + apply(psi_prey[, p], 1, mean) + apply(theta_prey[, t], 1, mean) + apply(tau_prey[, ta], 1, mean) + mean(samp_eff)

})

})

m <- do.call('rbind',

apply(m, 2, FUN =

**function**(j) {

tibble(mu = mean(j),

li = quantile(j, 0.025), ls = quantile(j, 0.975))

}, simplify = 'list'))

})

m$x <- x_seq

m$pr <- mean(post_total$beta_swarm_prey[[k]] < 0) m$location <- l

m

do.call('rbind', z)

})

swarm_effect_prey <- do.call('rbind', swarm_effect_prey) plot_swarm_effect_prey <-

lapply(seq_along(unique(swarm_effect_prey$location)), FUN	=

**function**(x) {

d <- unique(swarm_effect_prey[, c('pr', "location")])

swarm_effect_prey[swarm_effect_prey$location == d$location[x], ] |> ggplot(aes(x, mu, ymin = li, ymax = ls)) +

geom_line() + geom_ribbon(alpha = 0.5) +

facet_wrap(~location, scales = 'free') + labs(x = 'Swarm area (z-scores)',

y = 'Prey biomass (z-scores)',

title = paste('Pr(slope < 0) = ', round(d$pr[x], 3)))

})

plot_grid(plot_swarm_effect_prey[[1]],

plot_swarm_effect_prey[[2]], plot_swarm_effect_prey[[3]], plot_swarm_effect_prey[[4]], ncol = 2)

Pr(slope < 0) = 0.744	Pr(slope < 0) = 0.995

2

Prey biomass (z−scores) Prey biomass (z−scores)

Prey biomass (z−scores) Prey biomass (z−scores)

0.4

0.2

0.0

0	2	4

Swarm area (z−scores)

−0.6

−0.8

1

−1.0

−1.2

0	2	4

Swarm area (z−scores)

Pr(slope < 0) = 0.024

3

Pr(slope < 0) = 0.943

4

1.4

1.3

1.2

1.1

1.0

0.9

−0.5

−0.6

−0.7

−0.8

−0.9

−1.0

0	2	4	0	2	4

Swarm area (z−scores)	Swarm area (z−scores)

Figure S10: Effect response of profitability at different sections of the sawrm, as function of the swarm area

plot_grid(plot_swarm_effect_prey[[5]],

plot_swarm_effect_prey[[6]], plot_swarm_effect_prey[[7]], plot_swarm_effect_prey[[8]], ncol = 2)

Pr(slope < 0) = 1	Pr(slope < 0) = 0.631

6

Prey biomass (z−scores) Prey biomass (z−scores)

Prey biomass (z−scores) Prey biomass (z−scores)

−0.4

−0.8

−1.2

−1.6

0	2	4

Swarm area (z−scores)

−0.5

−0.6

5

−0.7

−0.8

−0.9

−1.0

0	2	4

Swarm area (z−scores)

Pr(slope < 0) = 0.09

7

Pr(slope < 0) = 0.288

8

−0.6

−0.8

−1.0

1.0

0.8

0.6

0.4

0	2	4	0	2	4

Swarm area (z−scores)	Swarm area (z−scores)

Figure S11: Effect of swarm area on its profitability across different sections of the swarm

### S6.6.2 Effect of swarm area on ant-followers

est_total_swarm <-

lapply(1, FUN =

**function**(x) {

x_seq <- seq(min(dat_generative2$z_swarm_area), max(dat_generative2$z_swarm_area), length.out = 100)

p <-

lapply(x_seq, FUN =

**function**(i) { v <-

with(post_total,

{

apply(alphaO, 1, mean) + apply(thetaO, 1, mean) + beta_swarmO[[1]] * i + apply(beta_preyO, 1, mean) * 0 + apply(tauO, 1, mean) + apply(psiO, 1, mean) + apply(deltaO, 1, mean)

})

v <- inv_logit(v) tibble(mu = mean(v),

li = quantile(v, 0.025),

ls = quantile(v, 0.975))

})

p <- do.call('rbind', p) p$x <- x_seq

p

})

est_total_swarm <- do.call('rbind', est_total_swarm)

𝛽 parameter and probability of effect

mean(post_total$beta_swarmO$beta_swarmO)

[1] 0.52961

sd(post_total$beta_swarmO$beta_swarmO)

[1] 0.1015288

mean(post_total$beta_swarmO$beta_swarmO > 0)

[1] 1

est_total_swarm |>

ggplot(aes(x, mu, ymin = li, ymax = ls)) + geom_line() +

geom_ribbon(alpha = 0.5) +

labs(x = 'Swarm area (z-scores)',

y = 'Pr(ant-follower presence)')

0.75

Pr(ant−follower presence)

0.50

0.25

0	2	4

Swarm area (z−scores)

Figure S12: Effect of swarm area on the probability of a swarm being used by an ant-follower bird

### S6.6.3 Effect of prey biomass on ant-followers

est_total_prey <-

lapply(1, FUN =

**function**(E) {

x_seq <- seq(min(dat_generative2$z_prey_biomass), max(dat_generative2$z_prey_biomass), length.out = 100)

p <-

lapply(x_seq, FUN =

**function**(i) { v <-

with(post_total,

{

apply(alphaO, 1, mean) + apply(thetaO, 1, mean) + apply(beta_swarmO, 1, mean) * 0 + beta_preyO[[1]] * i +

apply(tauO, 1, mean) + apply(psiO, 1, mean) + apply(deltaO, 1, mean)

})

v <- inv_logit(v) tibble(mu = mean(v),

li = quantile(v, 0.025), ls = quantile(v, 0.975))

})

p <- do.call('rbind', p) p$x <- x_seq

p

})

est_total_prey <- do.call('rbind', est_total_prey) est_total_prey |>

ggplot(aes(x, mu, ymin = li, ymax = ls)) +

geom_line() + geom_ribbon(alpha = 0.5) +

labs(x = 'Prey biomass (z-scores)',

y = 'Pr(ant-follower presence)')

0.8

0.6

Pr(ant−follower presence)

0.4

0.2

0	2	4

Prey biomass (z−scores)

Figure S13: Effect of prey biomass on the probability of a swarm being used by an ant-follower bird

𝛽 parameter and probability of effect

mean(post_total$beta_preyO[[1]])

[1] 0.1559427

sd(post_total$beta_preyO[[1]])

[1] 0.150459

mean(post_total$beta_preyO[[1]] > 0)

[1] 0.8468266

### S6.6.4 Space use across sections

est_section_total <-

**function**(x) {

lab <- which(group_codes$categories$code == x) lab <- group_codes$categories$label[lab]

v <-with(post_total,

{

alphaO[, x, drop = T] + apply(thetaO, 1, mean) + apply(beta_swarmO, 1, mean) * 0 +

apply(beta_preyO, 1, mean) * 0 + apply(tauO, 1, mean) + apply(psiO, 1, mean) + apply(deltaO, 1, mean)

})

v <- inv_logit(v)

tibble(mu = sample(v, 1e3), section = lab)

})

est_section_total <- do.call('rbind', est_section_total) # ===== Plot prey biomass and space use ===== colnames(codes_categories) <- c('section', 'category')

codes_categories$section <- as.factor(codes_categories$section)

est_section_total <-full_join(est_section_total,

codes_categories, by = c('section'))

p_use <-est_section_total |> group_by(section) |>

transmute(mean = mean(mu),

li = quantile(mu, 0.025),

ls = quantile(mu, 0.975)) |>

unique()

lapply(seq_along(post_total$alphaO), FUN =

lab_temp <-

unique(tibble(lab = birds4$categories,

section = as.factor(dat_generative2$categories)))

est_section_total <- full_join(est_section_total,

lab_temp, by = 'section')[, c(1, 3)]

colnames(est_section_total) <- colnames(est_prey1) est_section_total$mu_std <-

as.vector(scale(est_section_total$mean))

est_prey1$mu_std <- as.vector(scale(est_prey1$mean))

est_prey1$class <- "Profitability" est_section_total$class <- "Ant-follower's space use"

zz <-

est_prey1 |> group_by(category) |> transmute(mu = mean(mean)) |> unique() |> ungroup()

zz <- zz[order(zz$mu), ] produ_space_use <-

rbind(est_prey1,

est_section_total)

produ_space_use$category <- as.factor(produ_space_use$category) produ_space_use$category <-

factor(produ_space_use$category, levels = zz$category)

text <-

using each section or the outside of the swarm. O = outside,

'Probability and 95% credibility intervals of ant-followers

IF = interior front, IS = interior side, C = center,

IB =interior border, BP = back perimeter, SP = side perimeter, FP = front perimeter'

knitr::kable(

p_use[order(p_use$mean, decreasing = T), ], digits = 2,

caption = text

)

Table S5: Probability and 95% credibility intervals of ant-followers using each section or the outside of the swarm. O = outside, IF = interior front, IS = interior side, C = center, IB =interior border, BP = back perimeter, SP = side perimeter, FP = front perimeter

| section | mean li ls |
| --- | --- |
| 3 | 0.65 0.52 0.77 |
| 1 | 0.46 0.35 0.58 |
| 5 | 0.42 0.31 0.53 |
| 8 | 0.37 0.26 0.47 |
| 6 | 0.35 0.25 0.45 |

|  | section | mean li ls |
| --- | --- | --- |
|  | 4 | 0.35 0.23 0.46 |
|  | 2 | 0.25 0.16 0.36 |
|  | 7 | 0.18 0.10 0.28 |
| effect_prof <- |  |  |

do.call('rbind',

lapply(unique(est_section_total$category), FUN =

**function**(x) {

i <- est_section_total[est_section_total$category == 'FP',]$mean
j <- est_section_total[est_section_total$category == x, ]$mean
k <- ((i-j)/j) * 100 tibble(`Perc. higher` = mean(k),

`CI 2.5` = quantile(k, 0.025),

`CI 97.5%` = quantile(k, 0.975),

`P(diff > 0)` = mean(k > 0), section = x)

}))

effect_prof2 <-lapply(1, FUN =

**function**(x) {

i <- est_section_total[est_section_total$category == 'FP',]$mean
j <- est_section_total[est_section_total$category != 'FP', ]$mean
k <- ((i-j)/j) * 100 tibble(`Perc. higher` = mean(k),

`CI 2.5` = quantile(k, 0.025),

`CI 97.5%` = quantile(k, 0.975),

`P(diff > 0)` = mean(k > 0), section = 'Total')

})

knitr::kable(

rbind(effect_prof, effect_prof2[[1]]), digits = 2,

caption = 'Probability contrast between front perimeter and other swarm sections. The column Perc. higher in

)

Table S6: Probability contrast between front perimeter and other swarm sections. The column Perc. higher indicate the probability difference as relative percentage; P(diff > 0) shows the probability that the difference is above zero.

Perc. higher	CI 2.5	CI 97.5%	P(diff > 0)	section

| 42.34 | 1.32 | 90.69 | 0.98 BP |
| --- | --- | --- | --- |
| 171.01 | 70.46 | 320.73 | 1.00 C |
| 0.00 | 0.00 | 0.00 | 0.00 FP |
| 94.52 | 32.11 | 184.10 | 1.00 IB |
| 58.71 | 12.13 | 116.80 | 1.00 IF |
| 92.80 | 31.97 | 179.55 | 1.00 IS |

Perc. higher	CI 2.5	CI 97.5%	P(diff > 0)	section

|  | 283.87 | 117.98 | 556.54 | 1.00 | O |
| --- | --- | --- | --- | --- | --- |
|  | 80.18 | 25.56 | 153.64 | 1.00 | SP |
|  | 117.63 | 16.33 | 386.99 | 1.00 | Total |
| produ_space_use \|> |  |  |  |  |  |

group_by(class, category) |> transmute(mu = mean(mu_std),

li = quantile(mu_std, 0.025),

ls = quantile(mu_std, 0.975)) |> unique() |>

ggplot(aes(category, mu,

ymin = li, ymax = ls, color = class)) +

geom_errorbar(width = 0,

position = position_dodge(width = 0.4), linewidth = 1.5, alpha = 0.5) +

geom_point(position = position_dodge(width = 0.4), size = 2) +

scale_color_manual(values = c('#718444', '#D9631E')) + scale_x_discrete(limits = levels(produ_space_use$category),

labels =

c('Interior \nfront ', 'Interior \nside ', 'Interior \nback ', 'Center',

"Outside",

'Back \nperimeter', 'Side \nperimeter', 'Front \nperimeter')) +

theme_classic() +

labs(y = 'z-scores', x = 'Location within and outside the swarm') + theme(panel.grid = element_blank(),

legend.title = element_blank(), legend.position = c(0.2, 0.9),

#legend.background = element_rect(fill = '#f9f4da'), strip.background = element_blank(),

axis.line = element_line(linewidth = 0.25), axis.ticks = element_line(linewidth = 0.25),

#axis.text.x = element_text(angle = 45, hjust = 1, vjust = 1), axis.text = element_text(size = 8.5),

#title = element_blank(), legend.margin = margin(c(0, 0, 0, 0)))

Warning: A numeric `legend.position` argument in `theme()` was deprecated in ggplot2 3.5.0.

i Please use the `legend.position.inside` argument of `theme()` instead.

2

Ant−follower's space use Profitability

1

z−scores

0

−1

−2

Interior front

Interior side

Interior back

Center	Outside	Back

perimeter

Side perimeter

Front perimeter

Location within and outside the swarm

Figure S14: Variation of profitability and ant-followers space use outside and across the swarms.

# S7 Model 3: effect of dominance on ant-followers space use (species level)

This model focus on answering if dominance status affect ant-followers space use within and outside the swarm. Below, we provide the code for data formatting, model in mathematical notation, Stan code, fitting procedure and model diagnostics.

## S7.1 Data preparation

dat_generative1 <-

lapply(birds4[, c("date", "swarm_ID", "site", "round_number", "categories", "swarm_area", "mu_prey_biomas","BIAN", "GHET",

"OCAN", "SPAN", 'samp_eff_std')],

**function**(x) { x

})

group_codes <- lapply(dat_generative1[1:5], FUN =

**function**(x) {

x <- unique(x)

x <- as.factor(as.character(sort(x)))

})

tibble(label = x,

code = as.numeric(x))

**for** (i **in** 1:5) dat_generative1[[i]] <-as.numeric(as.factor(as.character(dat_generative1[[i]])))

dat_generative1$N <- length(dat_generative1$date) dat_generative1$N_days <- max(dat_generative1$date) dat_generative1$N_site <- max(dat_generative1$site) dat_generative1$N_swarm <- max(dat_generative1$swarm_ID) dat_generative1$N_round_number <- max(dat_generative1$round_number) dat_generative1$N_categories <- max(dat_generative1$categories) dat_generative1$dist_day <- dist_day_bird

dat_generative1$z_swarm_area <- as.vector(scale(dat_generative1$swarm_area)) dat_generative1$z_prey_biomass <- as.vector(scale(dat_generative1$mu_prey_biomas))

## S7.2 Mathematical notation

The following Bayesian hierarchical model asses the interrelated processes affecting the space use by the ant-followers assemblage (i.e. 𝑠𝑤𝑎𝑟𝑚 → 𝑝𝑟𝑒𝑦 𝑏𝑖𝑜𝑚𝑎𝑠𝑠; 𝑠𝑤𝑎𝑟𝑚 → 𝑏𝑖𝑟𝑑𝑠; 𝑠𝑤𝑎𝑟𝑚 →

𝑝𝑟𝑒𝑦 𝑏𝑖𝑜𝑚𝑎𝑠𝑠 → 𝑏𝑖𝑟𝑑𝑠; 𝑝𝑟𝑒𝑦 𝑏𝑖𝑜𝑚𝑎𝑠𝑠 → 𝑏𝑖𝑟𝑑𝑠; 𝑠𝑝𝑒𝑐𝑖𝑒𝑠_𝑖_ → 𝑠𝑝𝑒𝑐𝑖𝑒𝑠_𝑗_. Figure below).We used a

𝑆𝑡𝑢𝑑𝑒𝑛𝑡-𝑇 likelihood for modelling z-scores of swarm area and prey biomass, and employed a

𝐵𝑒𝑟𝑛𝑜𝑢𝑙𝑙𝑖 likelihood to model the probability of observing four ant-followers.In all models we used Gaussian processes to estimate 𝜃 parameters and account for temporal correlation. Other grouping factors were estimated using partial polling and a non-centered parametrization.

The DAG below explains the ant-followers space use by accounting for the hierarchy on the species dominance (figure below).

Mathematical notation of the above DAG

𝑆𝑤𝑎𝑟𝑚 𝑎𝑟𝑒𝑎𝑖 ∼ 𝑆𝑡𝑢𝑑𝑒𝑛𝑡𝑇(𝜈 = 2, 𝜇1𝑖, 𝜎1𝑖)

𝜇1𝑖 = 𝛼1[𝑠𝑤𝑎𝑟𝑚 𝑖] + 𝜓1[𝑠𝑖𝑡𝑒 𝑖]^+^

𝜏1[𝑟𝑜𝑢𝑛𝑑 𝑖] + 𝜃1[𝑑𝑎𝑡𝑒 𝑖]

𝑃𝑟𝑒𝑦 𝑏𝑖𝑜𝑚𝑎𝑠𝑠𝑖 ∼ 𝑆𝑡𝑢𝑑𝑒𝑛𝑡𝑇(𝜈 = 2, 𝜇1𝑖, 𝜎2𝑖)

𝜇1𝑖 = 𝛼2[𝑠𝑤𝑎𝑟𝑚 𝑖] + 𝜓2[𝑠𝑖𝑡𝑒 𝑖] + 𝛾[𝑠𝑒𝑐𝑡𝑖𝑜𝑛 𝑖]^+^

𝜏2[𝑟𝑜𝑢𝑛𝑑 𝑖] + 𝜃2[𝑑𝑎𝑡𝑒 𝑖]

𝛽3[𝑠𝑒𝑐𝑡𝑖𝑜𝑛 𝑖] × 𝑠𝑤𝑎𝑟𝑚 𝑎𝑟𝑒𝑎𝑖 + 𝑠𝑎𝑚𝑝𝑙𝑖𝑛𝑔 𝑒𝑓𝑓𝑜𝑟𝑡𝑖

𝑃. 𝑚𝑐𝑙𝑒𝑎𝑛𝑛𝑎𝑛𝑖 ∼ 𝐵𝑒𝑟𝑛𝑜𝑢𝑙𝑙𝑖(𝛾1 𝑖)

𝑙𝑜𝑔𝑖𝑡(𝛾1 𝑖) = 𝛼3[𝑠𝑒𝑐𝑡𝑖𝑜𝑛 𝑖] + 𝜓3[𝑠𝑖𝑡𝑒 𝑖] + 𝛿1[𝑠𝑤𝑎𝑟𝑚 𝑖]

𝜏3[𝑟𝑜𝑢𝑛𝑑 𝑖] + 𝜃3[𝑑𝑎𝑡𝑒 𝑖]^+^

𝛽1 × 𝑠𝑤𝑎𝑟𝑚 𝑎𝑟𝑒𝑎𝑖+

𝛽2 × 𝑝𝑟𝑒𝑦 𝑏𝑖𝑜𝑚𝑎𝑠𝑠𝑖

𝐸. 𝑝𝑒𝑛𝑖𝑐𝑖𝑙𝑙𝑎𝑡𝑎𝑖 ∼ 𝐵𝑒𝑟𝑛𝑜𝑢𝑙𝑙𝑖(𝛾2 𝑖)

𝑙𝑜𝑔𝑖𝑡(𝛾2 𝑖) = 𝛼4[𝑠𝑒𝑐𝑡𝑖𝑜𝑛 𝑖] + 𝜓4[𝑠𝑖𝑡𝑒 𝑖] + 𝛿2[𝑠𝑤𝑎𝑟𝑚 𝑖]

𝜏4[𝑟𝑜𝑢𝑛𝑑 𝑖] + 𝜃4[𝑑𝑎𝑡𝑒 𝑖]^+^

𝛽3 × 𝑠𝑤𝑎𝑟𝑚 𝑎𝑟𝑒𝑎𝑖+

𝛽4 × 𝑝𝑟𝑒𝑦 𝑏𝑖𝑜𝑚𝑎𝑠𝑠𝑖+

𝛽5 × 𝑃. 𝑚𝑐𝑙𝑒𝑎𝑛𝑛𝑎𝑛𝑖

𝐺. 𝑏𝑖𝑐𝑜𝑙𝑜𝑟𝑖 ∼ 𝐵𝑒𝑟𝑛𝑜𝑢𝑙𝑙𝑖(𝛾3 𝑖)

𝑙𝑜𝑔𝑖𝑡(𝛾3 𝑖) = 𝛼5[𝑠𝑒𝑐𝑡𝑖𝑜𝑛 𝑖] + 𝜓5[𝑠𝑖𝑡𝑒 𝑖] + 𝛿3[𝑠𝑤𝑎𝑟𝑚 𝑖]

𝜏5[𝑟𝑜𝑢𝑛𝑑 𝑖] + 𝜃5[𝑑𝑎𝑡𝑒 𝑖]^+^

𝛽4 × 𝑠𝑤𝑎𝑟𝑚 𝑎𝑟𝑒𝑎𝑖+

𝛽5 × 𝑝𝑟𝑒𝑦 𝑏𝑖𝑜𝑚𝑎𝑠𝑠𝑖+

𝛽6 × 𝑃. 𝑚𝑐𝑙𝑒𝑎𝑛𝑛𝑎𝑛𝑖+

𝛽7 × 𝐸. 𝑝𝑒𝑛𝑖𝑐𝑖𝑙𝑙𝑎𝑡𝑎𝑖

𝐻. 𝑛𝑎𝑒𝑣𝑖𝑜𝑖𝑑𝑒𝑠𝑖 ∼ 𝐵𝑒𝑟𝑛𝑜𝑢𝑙𝑙𝑖(𝛾4 𝑖)

𝑙𝑜𝑔𝑖𝑡(𝛾4 𝑖) = 𝛼6[𝑠𝑒𝑐𝑡𝑖𝑜𝑛 𝑖] + 𝜓6[𝑠𝑖𝑡𝑒 𝑖] + 𝛿4[𝑠𝑤𝑎𝑟𝑚 𝑖]

𝜏6[𝑟𝑜𝑢𝑛𝑑 𝑖] + 𝜃6[𝑑𝑎𝑡𝑒 𝑖]^+^

𝛽5 × 𝑠𝑤𝑎𝑟𝑚 𝑎𝑟𝑒𝑎𝑖+

𝛽6 × 𝑝𝑟𝑒𝑦 𝑏𝑖𝑜𝑚𝑎𝑠𝑠𝑖+

𝛽7 × 𝑃. 𝑚𝑐𝑙𝑒𝑎𝑛𝑛𝑎𝑛𝑖+

𝛽8 × 𝐸. 𝑝𝑒𝑛𝑖𝑐𝑖𝑙𝑙𝑎𝑡𝑎𝑖+

𝛽9 × 𝐺. 𝑏𝑖𝑐𝑜𝑙𝑜𝑟𝑖

𝛼1...𝛼6 = 𝜇𝛼1...𝛼6 + 𝑧𝛼1...𝛼6 × 𝜎𝛼1...𝛼6

𝜇𝛼_1_...𝛼_6_ ∼ 𝑁𝑜𝑟𝑚𝑎𝑙(0, 1)

𝑧𝛼_1_...𝛼_6_ ∼ 𝑁𝑜𝑟𝑚𝑎𝑙(0, 1)

𝜎𝛼_1_...𝛼_6_ ∼ 𝐸𝑥𝑝𝑜𝑛𝑒𝑛𝑡𝑖𝑎𝑙(1)

𝜏1...𝜏6 = 𝜇𝜏1...𝜏6 + 𝑧𝜏1...𝜏6 × 𝜎𝜏1...𝜏6

𝜇𝜏_1_...𝜏_6_ ∼ 𝑁𝑜𝑟𝑚𝑎𝑙(0, 1)

𝑧𝜏_1_...𝜏_6_ ∼ 𝑁𝑜𝑟𝑚𝑎𝑙(0, 1)

𝜎𝜏_1_...𝜏_6_ ∼ 𝐸𝑥𝑝𝑜𝑛𝑒𝑛𝑡𝑖𝑎𝑙(1)

𝜓1...𝜓6 = 𝜇𝜓1...𝜓6 + 𝑧𝜓1...𝜓6 × 𝜎𝜓1...𝜓6

𝜇𝜓_1_...𝜓_6_ ∼ 𝑁𝑜𝑟𝑚𝑎𝑙(0, 1)

𝑧𝜓_1_...𝜓_6_ ∼ 𝑁𝑜𝑟𝑚𝑎𝑙(0, 1)

𝜎𝜓_1_...𝜓_6_ ∼ 𝐸𝑥𝑝𝑜𝑛𝑒𝑛𝑡𝑖𝑎𝑙(1)

𝛿1...𝛿4 = 𝜇𝛿1...𝛿4 + 𝑧𝛿1...𝛿4 × 𝜎𝛿1...𝛿4

𝜇𝛿_1_...𝛿_4_ ∼ 𝑁𝑜𝑟𝑚𝑎𝑙(0, 1)

𝑧𝛿_1_...𝛿_4_ ∼ 𝑁𝑜𝑟𝑚𝑎𝑙(0, 1)

𝜎𝛿_1_...𝛿_4_ ∼ 𝐸𝑥𝑝𝑜𝑛𝑒𝑛𝑡𝑖𝑎𝑙(1)

𝛾𝑠𝑒𝑐𝑡𝑖𝑜𝑛 𝑖 = 𝜇𝛾 + 𝑧𝛾 × 𝜎𝛾	50

𝜇𝛾 ∼ 𝑁𝑜𝑟𝑚𝑎𝑙(0, 0.5)

𝑧𝛾 ∼ 𝑁𝑜𝑟𝑚𝑎𝑙(0, 0.5)

𝜎𝛾 ∼ 𝐸𝑥𝑝𝑜𝑛𝑒𝑛𝑡𝑖𝑎𝑙(1)

⎛ 𝜃1𝑑𝑎𝑦 1 ⎞

⎛ 𝜃6𝑑𝑎𝑦 1 ⎞

⎛	^0^	⎞⎟

⎜ 𝜃1𝑑𝑎𝑦 2

⎟ , ..., ⎜

𝜃6𝑑𝑎𝑦 2

⎟ ∼ 𝑀𝑉 𝑁𝑜𝑟𝑚𝑎𝑙 ⎜ ⎡⎢ 0 ⎤⎥ 𝐾𝜃1...𝜃6 ⎟

⎜	...	⎟

⎜	...	⎟

⎢...⎥

⎝ 𝜃1𝑑𝑎𝑦 𝑛 ⎠

𝜃1...𝜃6

⎝ 𝜃6𝑑𝑎𝑦 𝑛 ⎠

⎝ ⎣ 0 ⎦	⎠

𝐾𝜃1...𝜃6 = 𝜂2

𝜃1...𝜃6

𝜂𝜃1...𝜃6 ∼ 𝐸𝑥𝑝𝑜𝑛𝑒𝑛𝑡𝑖𝑎𝑙(3)

𝑒𝑥𝑝(−𝜌^2^

2

𝑑𝑎𝑦𝑠 𝑖𝑗

𝐷

) + 𝛿𝜃1...𝜃6 𝑖𝑗 × 0.01

## S7.3 Stan code

cat(file = 'generative_mod1.stan',

"

functions {

}

matrix cov_GPL2(matrix x,

real eta, real rho, real delta) {

int N = dims(x)[1];

matrix[N, N] k;

for (i in 1:(N-1)) { k[i, i] = eta + delta; for (j in (i + 1):N) {

k[i, j] = eta * exp(-rho * square(x[i, j])); k[j, i] = k[i, j];

}

}

k[N, N] = eta + delta; return k;

}

data {

int N;

int N_swarm; int N_site;

int N_round_number; int N_days;

int N_categories;

matrix[N_days, N_days] dist_day; array[N] int date;

array[N] int swarm_ID; array[N] int site; array[N] int round_number; array[N] int categories; vector[N] z_prey_biomass; vector[N] z_swarm_area; vector[N] samp_eff_std; array[N] int BIAN; array[N] int GHET; array[N] int OCAN; array[N] int SPAN;

}

parameters {

// ant swarm

vector[N_swarm] z_alpha_swarm; real mu_alpha_swarm;

real<lower = 0> sigma_alpha_swarm;

vector[N_site] z_psi_swarm; real mu_psi_swarm;

real<lower = 0> sigma_psi_swarm;

vector[N_round_number] z_tau_swarm; real mu_tau_swarm;

real<lower = 0> sigma_tau_swarm;

vector[N_days] z_theta_swarm;

real<lower = 0> eta_theta_swarm; real<lower = 0> rho_theta_swarm;

real<lower = 0> sigma_swarm;

// prey biomass

vector[N_swarm] z_alpha_prey; real mu_alpha_prey;

real<lower = 0> sigma_alpha_prey;

vector[N_site] z_psi_prey; real mu_psi_prey;

real<lower = 0> sigma_psi_prey;

vector[N_round_number] z_tau_prey; real mu_tau_prey;

real<lower = 0> sigma_tau_prey;

vector[N_categories] z_gamma_prey; real mu_gamma_prey;

real<lower = 0> sigma_gamma_prey;

vector[N_days] z_theta_prey; real<lower = 0> eta_theta_prey; real<lower = 0> rho_theta_prey;

vector[N_categories] beta_swarm_prey; real<lower = 0> sigma_prey;

// P. mcleannani (OCAN) real beta_swarmO;

real beta_preyO;

vector[N_categories] z_alphaO; real mu_alphaO;

real<lower = 0> sigma_alphaO;

vector[N_days] z_thetaO; real<lower = 0> eta_thetaO; real<lower = 0> rho_thetaO;

vector[N_round_number] z_tauO; real mu_tauO;

real<lower = 0> sigma_tauO;

vector[N_site] z_psiO; real mu_psiO;

real<lower = 0> sigma_psiO;

vector[N_swarm] z_deltaO; real mu_deltaO;

real<lower = 0> sigma_deltaO;

// E. penicillata (GHET) real beta_swarmE;

real beta_preyE;

vector[N_categories] beta_OCAN_E;

vector[N_categories] z_alphaE; real mu_alphaE;

real<lower = 0> sigma_alphaE;

vector[N_days] z_thetaE; real<lower = 0> eta_thetaE; real<lower = 0> rho_thetaE;

vector[N_round_number] z_tauE; real mu_tauE;

real<lower = 0> sigma_tauE;

vector[N_site] z_psiE; real mu_psiE;

real<lower = 0> sigma_psiE;

vector[N_swarm] z_deltaE; real mu_deltaE;

real<lower = 0> sigma_deltaE;

// G. bicolor (BIAN)

real beta_swarmB; real beta_preyB;

vector[N_categories] beta_OCAN_B; vector[N_categories] beta_GHET_B;

vector[N_categories] z_alphaB; real mu_alphaB;

real<lower = 0> sigma_alphaB;

vector[N_days] z_thetaB; real<lower = 0> eta_thetaB; real<lower = 0> rho_thetaB;

vector[N_round_number] z_tauB; real mu_tauB;

real<lower = 0> sigma_tauB;

vector[N_site] z_psiB; real mu_psiB;

real<lower = 0> sigma_psiB;

vector[N_swarm] z_deltaB; real mu_deltaB;

real<lower = 0> sigma_deltaB;

// H. naevioides (SPAN) real beta_swarmS;

real beta_preyS;

vector[N_categories] beta_OCAN_S; vector[N_categories] beta_GHET_S; vector[N_categories] beta_BIAN_S;

vector[N_categories] z_alphaS; real mu_alphaS;

real<lower = 0> sigma_alphaS;

vector[N_days] z_thetaS; real<lower = 0> eta_thetaS; real<lower = 0> rho_thetaS;

vector[N_round_number] z_tauS; real mu_tauS;

real<lower = 0> sigma_tauS;

vector[N_site] z_psiS; real mu_psiS;

real<lower = 0> sigma_psiS;

vector[N_swarm] z_deltaS; real mu_deltaS;

real<lower = 0> sigma_deltaS;

}

transformed parameters {

// ant swarm

vector[N_days] theta_swarm; matrix[N_days, N_days] sigma_theta_swarm;

matrix[N_days, N_days] L_sigma_theta_swarm; sigma_theta_swarm = cov_GPL2(dist_day,

eta_theta_swarm, rho_theta_swarm, 0.01);

L_sigma_theta_swarm = cholesky_decompose(sigma_theta_swarm); theta_swarm = L_sigma_theta_swarm * z_theta_swarm;

vector[N_swarm] alpha_swarm;

alpha_swarm = mu_alpha_swarm + z_alpha_swarm * sigma_alpha_swarm;

vector[N_site] psi_swarm;

psi_swarm = mu_psi_swarm + z_psi_swarm * sigma_psi_swarm;

vector[N_round_number] tau_swarm;

tau_swarm = mu_tau_swarm + z_tau_swarm * sigma_tau_swarm;

// prey biomass vector[N_days] theta_prey;

matrix[N_days, N_days] sigma_theta_prey;

matrix[N_days, N_days] L_sigma_theta_prey; sigma_theta_prey = cov_GPL2(dist_day,

eta_theta_prey, rho_theta_prey, 0.01);

L_sigma_theta_prey = cholesky_decompose(sigma_theta_prey); theta_prey = L_sigma_theta_prey * z_theta_prey;

vector[N_swarm] alpha_prey;

alpha_prey = mu_alpha_prey + z_alpha_prey * sigma_alpha_prey;

vector[N_site] psi_prey;

psi_prey = mu_psi_prey + z_psi_prey * sigma_psi_prey;

vector[N_round_number] tau_prey;

tau_prey = mu_tau_prey + z_tau_prey * sigma_tau_prey;

vector[N_categories] gamma_prey;

gamma_prey = mu_tau_prey + z_gamma_prey * sigma_gamma_prey;

// P. mcleannani (OCAN)

vector[N_days] thetaO; matrix[N_days, N_days] sigma_thetaO;

matrix[N_days, N_days] L_sigma_thetaO;

sigma_thetaO = cov_GPL2(dist_day, eta_thetaO, rho_thetaO, 0.01); L_sigma_thetaO = cholesky_decompose(sigma_thetaO);

thetaO = L_sigma_thetaO * z_thetaO;

vector[N_round_number] tauO;

tauO = mu_tauO + z_tauO * sigma_tauO;

vector[N_site] psiO;

psiO = mu_psiO + z_psiO * sigma_psiO;

vector[N_swarm] deltaO;

deltaO = mu_deltaO + z_deltaO * sigma_deltaO;

vector[N_categories] alphaO;

alphaO = mu_alphaO + z_alphaO * sigma_alphaO;

// E. penicillata (GHET)

vector[N_days] thetaE; matrix[N_days, N_days] sigma_thetaE;

matrix[N_days, N_days] L_sigma_thetaE;

sigma_thetaE = cov_GPL2(dist_day, eta_thetaE, rho_thetaE, 0.01); L_sigma_thetaE = cholesky_decompose(sigma_thetaE);

thetaE = L_sigma_thetaE * z_thetaE;

vector[N_round_number] tauE;

tauE = mu_tauE + z_tauE * sigma_tauE;

vector[N_site] psiE;

psiE = mu_psiE + z_psiE * sigma_psiE;

vector[N_swarm] deltaE;

deltaE = mu_deltaE + z_deltaE * sigma_deltaE;

vector[N_categories] alphaE;

alphaE = mu_alphaE + z_alphaE * sigma_alphaE;

// G. bicolor (BIAN) vector[N_days] thetaB;

matrix[N_days, N_days] sigma_thetaB;

matrix[N_days, N_days] L_sigma_thetaB;

sigma_thetaB = cov_GPL2(dist_day, eta_thetaB, rho_thetaB, 0.01); L_sigma_thetaB = cholesky_decompose(sigma_thetaB);

thetaB = L_sigma_thetaB * z_thetaB;

vector[N_round_number] tauB;

tauB = mu_tauB + z_tauB * sigma_tauB;

vector[N_site] psiB;

psiB = mu_psiB + z_psiB * sigma_psiB;

vector[N_swarm] deltaB;

deltaB = mu_deltaB + z_deltaB * sigma_deltaB;

vector[N_categories] alphaB;

alphaB = mu_alphaB + z_alphaB * sigma_alphaB;

// H. naevioides (SPAN)

vector[N_days] thetaS; matrix[N_days, N_days] sigma_thetaS;

matrix[N_days, N_days] L_sigma_thetaS;

sigma_thetaS = cov_GPL2(dist_day, eta_thetaS, rho_thetaS, 0.01); L_sigma_thetaS = cholesky_decompose(sigma_thetaS);

thetaS = L_sigma_thetaS * z_thetaS;

vector[N_round_number] tauS;

tauS = mu_tauS + z_tauS * sigma_tauS;

vector[N_site] psiS;

psiS = mu_psiS + z_psiS * sigma_psiS;

vector[N_swarm] deltaS;

deltaS = mu_deltaS + z_deltaS * sigma_deltaS;

vector[N_categories] alphaS;

alphaS = mu_alphaS + z_alphaS * sigma_alphaS;

}

model {

// ant swarm

z_alpha_swarm ~ normal(0, 1); mu_alpha_swarm ~ normal(0, 1); sigma_alpha_swarm ~ exponential(1);

z_psi_swarm ~ normal(0, 1); mu_psi_swarm ~ normal(0, 1); sigma_psi_swarm ~ exponential(1);

z_theta_swarm ~ normal(0, 0.5); eta_theta_swarm ~ exponential(3); rho_theta_swarm ~ exponential(3);

z_tau_swarm ~ normal(0, 1); mu_tau_swarm ~ normal(0, 1); sigma_tau_swarm ~ exponential(1);

sigma_swarm ~ exponential(1);

// prey biomass

beta_swarm_prey ~ normal(0, 1);

z_alpha_prey ~ normal(0, 1); mu_alpha_prey ~ normal(0, 1); sigma_alpha_prey ~ exponential(1);

z_psi_prey ~ normal(0, 1); mu_psi_prey ~ normal(0, 1); sigma_psi_prey ~ exponential(1);

z_theta_prey ~ normal(0, 1); eta_theta_prey ~ exponential(3); rho_theta_prey ~ exponential(3);

z_tau_prey ~ normal(0, 0.5); mu_tau_prey ~ normal(0, 0.5); sigma_tau_prey ~ exponential(1);

z_gamma_prey ~ normal(0, 0.5); mu_gamma_prey ~ normal(0, 0.5); sigma_gamma_prey ~ exponential(1);

sigma_prey ~ exponential(1);

// P. mcleannani (OCAN)

beta_swarmO ~ normal(0, 1.5); beta_preyO ~ normal(0, 1.5);

z_alphaO ~ normal(0, 1); mu_alphaO ~ normal(0, 1); sigma_alphaO ~ exponential(1);

z_thetaO ~ normal(0, 1); eta_thetaO ~ exponential(3); rho_thetaO ~ exponential(3);

z_tauO ~ normal(0, 1);

mu_tauO ~ normal(0, 1); sigma_tauO ~ exponential(1);

z_psiO ~ normal(0, 1); mu_psiO ~ normal(0, 1); sigma_psiO ~ exponential(1);

z_deltaO ~ normal(0, 1); mu_psiO ~ normal(0, 1); sigma_psiO ~ exponential(1);

// E. penicillata (GHET)

beta_swarmE ~ normal(0, 1.5); beta_preyE ~ normal(0, 1.5); beta_OCAN_E ~ normal(0, 0.5);

z_alphaE ~ normal(0, 1); mu_alphaE ~ normal(0, 1); sigma_alphaE ~ exponential(1);

z_thetaE ~ normal(0, 1); eta_thetaE ~ exponential(3); rho_thetaE ~ exponential(3);

z_tauE ~ normal(0, 1); mu_tauE ~ normal(0, 1); sigma_tauE ~ exponential(1);

z_psiE ~ normal(0, 1); mu_psiE ~ normal(0, 1); sigma_psiE ~ exponential(1);

z_deltaE ~ normal(0, 1); mu_psiE ~ normal(0, 1); sigma_psiE ~ exponential(1);

// G. bicolor (BIAN)

beta_swarmB ~ normal(0, 1.5); beta_preyB ~ normal(0, 1.5); beta_OCAN_B ~ normal(0, 0.5); beta_GHET_B ~ normal(0, 0.5);

z_alphaB ~ normal(0, 1); mu_alphaB ~ normal(0, 1); sigma_alphaB ~ exponential(1);

z_thetaB ~ normal(0, 1); eta_thetaB ~ exponential(3); rho_thetaB ~ exponential(3);

z_tauB ~ normal(0, 1); mu_tauB ~ normal(0, 1); sigma_tauB ~ exponential(1);

z_psiB ~ normal(0, 1); mu_psiB ~ normal(0, 1); sigma_psiB ~ exponential(1);

z_deltaB ~ normal(0, 1); mu_psiB ~ normal(0, 1); sigma_psiB ~ exponential(1);

// H. naevioides (SPAN) beta_swarmS ~ normal(0, 1.5);

beta_preyS ~ normal(0, 1.5); beta_OCAN_S ~ normal(0, 0.5); beta_GHET_S ~ normal(0, 0.5); beta_BIAN_S ~ normal(0, 0.5);

z_alphaS ~ normal(0, 1); mu_alphaS ~ normal(0, 1); sigma_alphaS ~ exponential(1);

z_thetaS ~ normal(0, 1); eta_thetaS ~ exponential(3); rho_thetaS ~ exponential(3);

z_tauS ~ normal(0, 1); mu_tauS ~ normal(0, 1); sigma_tauS ~ exponential(1);

z_psiS ~ normal(0, 1); mu_psiS ~ normal(0, 1); sigma_psiS ~ exponential(1);

z_deltaS ~ normal(0, 1); mu_psiS ~ normal(0, 1); sigma_psiS ~ exponential(1);

for (i in 1:N) {

z_swarm_area[i] ~ student_t(2, alpha_swarm[swarm_ID[i]] +

psi_swarm[site[i]] + theta_swarm[date[i]] + tau_swarm[round_number[i]], sigma_swarm);

}

for (i in 1:N) {

z_prey_biomass[i] ~ student_t(2, alpha_prey[swarm_ID[i]] +

gamma_prey[categories[i]] + beta_swarm_prey[categories[i]]*z_swarm_area[i] + psi_prey[site[i]] +

theta_prey[date[i]] + tau_prey[round_number[i]] + samp_eff_std[i], sigma_prey);

}

for (i in 1:N) {

OCAN[i] ~ bernoulli(inv_logit(alphaO[categories[i]] +

beta_swarmO * z_swarm_area[i] + beta_preyO * z_prey_biomass[i] + psiO[site[i]] + deltaO[swarm_ID[i]] +

thetaO[date[i]] + tauO[round_number[i]]));

}

for (i in 1:N) {

GHET[i] ~ bernoulli(inv_logit(alphaE[categories[i]] +

beta_swarmE * z_swarm_area[i] + beta_OCAN_E[categories[i]] * OCAN[i] + beta_preyE * z_prey_biomass[i] + psiE[site[i]] + deltaE[swarm_ID[i]] + thetaE[date[i]] + tauE[round_number[i]]));

}

for (i in 1:N) {

BIAN[i] ~ bernoulli(inv_logit(alphaB[categories[i]] +

beta_swarmB * z_swarm_area[i] + beta_preyB * z_prey_biomass[i] + beta_OCAN_B[categories[i]] * OCAN[i] + beta_GHET_B[categories[i]] * GHET[i] +

}

for (i in 1:N) {

psiB[site[i]] + deltaB[swarm_ID[i]] + thetaB[date[i]] + tauB[round_number[i]]));

SPAN[i] ~ bernoulli(inv_logit(alphaS[categories[i]] +

beta_swarmS * z_swarm_area[i] + beta_preyS * z_prey_biomass[i] + beta_OCAN_S[categories[i]] * OCAN[i] + beta_GHET_S[categories[i]] * GHET[i] + beta_BIAN_S[categories[i]] * BIAN[i] + psiS[site[i]] + deltaS[swarm_ID[i]] +

thetaS[date[i]] + tauS[round_number[i]]));

}

}

generated quantities { vector[N] muB; vector[N] muO; array[N] int ppcheckO; array[N] int ppcheckB; vector[N] mu_swarm;

vector[N] ppcheck_swarm; vector[N] mu_prey; vector[N] ppcheck_prey; vector[N] muE;

array[N] int ppcheckE; vector[N] muS; array[N] int ppcheckS;

for (i in 1:N) {

mu_swarm[i] = alpha_swarm[swarm_ID[i]] +

psi_swarm[site[i]] + theta_swarm[date[i]] + tau_swarm[round_number[i]];

}

for (i in 1:N) {

ppcheck_swarm[i] = student_t_rng(2, mu_swarm[i], sigma_swarm);

}

for (i in 1:N) {

mu_prey[i] = alpha_prey[swarm_ID[i]] +

gamma_prey[categories[i]] + beta_swarm_prey[categories[i]]*z_swarm_area[i] + psi_prey[site[i]] +

theta_prey[date[i]] + tau_prey[round_number[i]] + samp_eff_std[i];

}

for (i in 1:N) {

ppcheck_prey[i] = student_t_rng(2, mu_prey[i], sigma_prey);

}

for (i in 1:N) {

muO[i] = alphaO[categories[i]] + beta_swarmO * z_swarm_area[i] + beta_preyO * z_prey_biomass[i] +

psiO[site[i]] + deltaO[swarm_ID[i]] + thetaO[date[i]] + tauO[round_number[i]];

muO[i] = inv_logit(muO[i]);

}

for (i in 1:N) {

}

ppcheckO[i] = bernoulli_rng(muO[i]);

for (i in 1:N) {

muE[i] = alphaE[categories[i]] + beta_swarmE * z_swarm_area[i] +

beta_OCAN_E[categories[i]] * OCAN[i] + beta_preyE * z_prey_biomass[i] + psiE[site[i]] + deltaE[swarm_ID[i]] + thetaE[date[i]] + tauE[round_number[i]];

muE[i] = inv_logit(muE[i]);

}

for (i in 1:N) {

ppcheckE[i] = bernoulli_rng(muE[i]);

}

for (i in 1:N) {

muB[i] = alphaB[categories[i]] + beta_swarmB * z_swarm_area[i] + beta_preyB * z_prey_biomass[i] +

beta_OCAN_B[categories[i]] * OCAN[i] + beta_GHET_B[categories[i]] * GHET[i] + psiB[site[i]] + deltaB[swarm_ID[i]] + thetaB[date[i]] + tauB[round_number[i]];

muB[i] = inv_logit(muB[i]);

}

for (i in 1:N) {

ppcheckB[i] = bernoulli_rng(muB[i]);

}

for (i in 1:N) {

muS[i] = alphaS[categories[i]] + beta_swarmS * z_swarm_area[i] + beta_preyS * z_prey_biomass[i] +

beta_OCAN_S[categories[i]] * OCAN[i] + beta_GHET_S[categories[i]] * GHET[i] + beta_BIAN_S[categories[i]] * BIAN[i] + psiS[site[i]] + deltaS[swarm_ID[i]] + thetaS[date[i]] + tauS[round_number[i]];

muS[i] = inv_logit(muS[i]);

}

for (i in 1:N) {

ppcheckS[i] = bernoulli_rng(muS[i]);

}

} ")

## S7.4 Fitting procedure

file <- paste(getwd(), '/generative_mod1.stan', sep ='') fit_generative1 <- cmdstan_model(file, compile = T)

mod <-

fit_generative1$sample( data = dat_generative1,

)

chains = 3,

iter_warmup = 500,

iter_sampling = 3500,

parallel_chains = 3,

thin = 3,

refresh = 50,

seed = 123

## S7.5 Summary

pars <- c('beta_swarm', 'beta_prey', 'alpha', 'theta', 'tau', 'psi', 'delta')

pars2 <- c('alpha', 'theta', 'tau', 'psi', 'sigma') pars <- c(paste(pars2, '_swarm', sep = ''),

paste(pars2, '_prey', sep = ''),

paste(pars, 'O', sep = ''),

paste(pars, 'B', sep = ''),

paste(pars, 'E', sep = ''),

paste(pars, 'S', sep = ''), 'gamma_prey', 'beta_swarm_prey', 'beta_OCAN_B', 'beta_OCAN_E', 'beta_GHET_B', 'beta_OCAN_S', 'beta_GHET_S', 'beta_BIAN_S')

summary_mod <- mod$summary(pars) knitr::kable(

rbind(summary_mod[grep('OCAN|GHET|BIAN', summary_mod$variable), ],

summary_mod[grep('beta_swarm', summary_mod$variable), ], summary_mod[grep('beta_prey', summary_mod$variable), ], summary_mod[grep('beta_swarm_prey', summary_mod$variable), ], summary_mod[grep('alpha', summary_mod$variable), ], summary_mod[grep('gamma', summary_mod$variable), ]),

digits = 2,

caption = 'Estimated coefficients and sampling diagnostics of the model assessing the interrelated processes

)

Table S7: Estimated coeﬀicients and sampling diagnostics of the model assessing the interre-lated processes affecting the space use by the ant-followers assemblage

| variable | mean | median | sd | mad | q5 | q95 | rhat | ess_bulk | ess_tail |
| --- | --- | --- | --- | --- | --- | --- | --- | --- | --- |
| beta_OCAN_B[1] | -0.27 | -0.27 | 0.49 | 0.48 | -1.07 | 0.54 | 1 | 4004.64 | 3090.50 |
| beta_OCAN_B[2] | 0.01 | 0.00 | 0.50 | 0.49 | -0.82 | 0.84 | 1 | 4157.64 | 2922.60 |
| beta_OCAN_B[3] | -0.85 | -0.85 | 0.42 | 0.40 | -1.52 | -0.16 | 1 | 4021.33 | 3194.29 |
| beta_OCAN_B[4] | -0.34 | -0.33 | 0.46 | 0.46 | -1.12 | 0.41 | 1 | 4011.32 | 3218.33 |
| beta_OCAN_B[5] | -0.38 | -0.37 | 0.46 | 0.46 | -1.13 | 0.36 | 1 | 3800.35 | 3261.19 |
| beta_OCAN_B[6] | -0.43 | -0.43 | 0.44 | 0.45 | -1.18 | 0.29 | 1 | 4286.61 | 3046.52 |
| beta_OCAN_B[7] | -0.04 | -0.03 | 0.49 | 0.48 | -0.85 | 0.77 | 1 | 4075.03 | 3412.18 |
| beta_OCAN_B[8] | -0.21 | -0.20 | 0.47 | 0.47 | -0.99 | 0.52 | 1 | 3819.92 | 3113.29 |
| beta_OCAN_E[1] | -0.01 | -0.01 | 0.50 | 0.49 | -0.84 | 0.82 | 1 | 3951.55 | 3239.68 |

| variable | mean | median | sd | mad | q5 | q95 | rhat | ess_bulk | ess_tail |
| --- | --- | --- | --- | --- | --- | --- | --- | --- | --- |
| beta_OCAN_E[2] | -0.01 | -0.02 | 0.50 | 0.50 | -0.83 | 0.80 | 1 | 4092.33 | 3306.98 |
| beta_OCAN_E[3] | -0.07 | -0.08 | 0.48 | 0.48 | -0.87 | 0.71 | 1 | 3880.82 | 3199.68 |
| beta_OCAN_E[4] | 0.00 | 0.00 | 0.51 | 0.51 | -0.85 | 0.86 | 1 | 4337.84 | 3308.27 |
| beta_OCAN_E[5] | -0.02 | -0.02 | 0.50 | 0.49 | -0.85 | 0.81 | 1 | 3547.11 | 3121.74 |
| beta_OCAN_E[6] | -0.02 | -0.01 | 0.49 | 0.49 | -0.83 | 0.78 | 1 | 3879.22 | 3233.03 |
| beta_OCAN_E[7] | -0.03 | -0.03 | 0.49 | 0.48 | -0.84 | 0.77 | 1 | 4305.91 | 2912.50 |
| beta_OCAN_E[8] | -0.06 | -0.06 | 0.50 | 0.50 | -0.86 | 0.78 | 1 | 4135.39 | 3221.16 |
| beta_GHET_B[1] | 0.02 | 0.02 | 0.50 | 0.49 | -0.81 | 0.85 | 1 | 3988.64 | 3209.00 |
| beta_GHET_B[2] | -0.12 | -0.13 | 0.48 | 0.48 | -0.91 | 0.67 | 1 | 3921.14 | 3253.42 |
| beta_GHET_B[3] | -0.41 | -0.41 | 0.44 | 0.45 | -1.13 | 0.32 | 1 | 3970.09 | 3421.09 |
| beta_GHET_B[4] | -0.10 | -0.10 | 0.47 | 0.46 | -0.86 | 0.69 | 1 | 3764.15 | 2958.35 |
| beta_GHET_B[5] | -0.24 | -0.23 | 0.46 | 0.46 | -1.01 | 0.53 | 1 | 4265.63 | 3066.37 |
| beta_GHET_B[6] | -0.06 | -0.06 | 0.50 | 0.50 | -0.87 | 0.75 | 1 | 4008.04 | 3192.31 |
| beta_GHET_B[7] | -0.04 | -0.03 | 0.49 | 0.49 | -0.85 | 0.76 | 1 | 3995.84 | 3350.84 |
| beta_GHET_B[8] | -0.24 | -0.24 | 0.47 | 0.46 | -1.02 | 0.53 | 1 | 4100.87 | 3190.62 |
| beta_OCAN_S[1] | -0.02 | -0.01 | 0.50 | 0.50 | -0.85 | 0.78 | 1 | 3884.70 | 3288.94 |
| beta_OCAN_S[2] | 0.01 | 0.02 | 0.51 | 0.52 | -0.85 | 0.84 | 1 | 4245.33 | 3309.81 |
| beta_OCAN_S[3] | -0.15 | -0.15 | 0.47 | 0.47 | -0.92 | 0.62 | 1 | 3926.51 | 3478.18 |
| beta_OCAN_S[4] | -0.04 | -0.04 | 0.48 | 0.48 | -0.82 | 0.76 | 1 | 3863.56 | 3268.73 |
| beta_OCAN_S[5] | -0.04 | -0.05 | 0.49 | 0.49 | -0.83 | 0.75 | 1 | 3967.10 | 3295.45 |
| beta_OCAN_S[6] | -0.04 | -0.04 | 0.49 | 0.48 | -0.84 | 0.76 | 1 | 4107.62 | 3093.14 |
| beta_OCAN_S[7] | 0.00 | 0.00 | 0.50 | 0.49 | -0.82 | 0.83 | 1 | 4054.00 | 3300.40 |
| beta_OCAN_S[8] | -0.09 | -0.08 | 0.48 | 0.48 | -0.87 | 0.70 | 1 | 3886.09 | 3176.32 |
| beta_GHET_S[1] | 0.00 | 0.00 | 0.50 | 0.49 | -0.84 | 0.82 | 1 | 4147.56 | 3416.89 |
| beta_GHET_S[2] | -0.02 | -0.03 | 0.50 | 0.50 | -0.85 | 0.81 | 1 | 4352.57 | 3190.58 |
| beta_GHET_S[3] | -0.06 | -0.05 | 0.49 | 0.48 | -0.85 | 0.74 | 1 | 4193.97 | 3333.23 |
| beta_GHET_S[4] | -0.01 | 0.00 | 0.50 | 0.50 | -0.82 | 0.79 | 1 | 4215.73 | 3084.56 |
| beta_GHET_S[5] | -0.02 | -0.02 | 0.50 | 0.50 | -0.83 | 0.77 | 1 | 4342.45 | 3380.39 |
| beta_GHET_S[6] | 0.00 | 0.00 | 0.51 | 0.51 | -0.84 | 0.84 | 1 | 4135.02 | 3099.53 |
| beta_GHET_S[7] | -0.02 | -0.02 | 0.50 | 0.50 | -0.83 | 0.82 | 1 | 4104.11 | 3173.87 |
| beta_GHET_S[8] | -0.05 | -0.06 | 0.49 | 0.49 | -0.85 | 0.76 | 1 | 4013.67 | 3377.45 |
| beta_BIAN_S[1] | -0.43 | -0.42 | 0.44 | 0.45 | -1.15 | 0.28 | 1 | 4101.05 | 3232.44 |
| beta_BIAN_S[2] | -0.10 | -0.09 | 0.47 | 0.46 | -0.88 | 0.66 | 1 | 4040.15 | 3332.36 |
| beta_BIAN_S[3] | -0.63 | -0.64 | 0.42 | 0.42 | -1.33 | 0.08 | 1 | 3703.41 | 2953.06 |
| beta_BIAN_S[4] | -0.12 | -0.12 | 0.48 | 0.47 | -0.91 | 0.66 | 1 | 4144.45 | 3334.68 |
| beta_BIAN_S[5] | -0.16 | -0.16 | 0.47 | 0.47 | -0.92 | 0.57 | 1 | 3451.65 | 3023.34 |
| beta_BIAN_S[6] | -0.11 | -0.11 | 0.47 | 0.46 | -0.86 | 0.64 | 1 | 3870.26 | 3161.96 |
| beta_BIAN_S[7] | -0.05 | -0.05 | 0.49 | 0.49 | -0.84 | 0.74 | 1 | 4024.47 | 3305.28 |
| beta_BIAN_S[8] | -0.18 | -0.17 | 0.47 | 0.48 | -0.97 | 0.58 | 1 | 4003.41 | 3063.19 |
| beta_swarmO | 0.63 | 0.63 | 0.22 | 0.22 | 0.28 | 1.00 | 1 | 3772.88 | 3585.55 |
| beta_swarmB | 0.35 | 0.36 | 0.10 | 0.09 | 0.20 | 0.51 | 1 | 3759.08 | 3538.18 |

| variable | mean | median | sd | mad | q5 | q95 | rhat | ess_bulk | ess_tail |
| --- | --- | --- | --- | --- | --- | --- | --- | --- | --- |
| beta_swarmE | -0.17 | -0.17 | 0.32 | 0.32 | -0.72 | 0.35 | 1 | 3602.83 | 3262.57 |
| beta_swarmS | 0.06 | 0.07 | 0.27 | 0.27 | -0.40 | 0.49 | 1 | 3390.67 | 3299.53 |
| beta_swarm_prey[1]-0.02 | | -0.02 | 0.03 | 0.03 | -0.06 | 0.03 | 1 | 3402.02 | 3281.40 |
| beta_swarm_prey[2]-0.07 | | -0.07 | 0.03 | 0.03 | -0.12 | -0.02 | 1 | 3561.22 | 3225.42 |
| beta_swarm_prey[3] 0.04 | | 0.04 | 0.02 | 0.02 | 0.01 | 0.07 | 1 | 3916.36 | 3193.65 |
| beta_swarm_prey[4]-0.04 | | -0.04 | 0.02 | 0.02 | -0.07 | 0.00 | 1 | 4105.89 | 3363.66 |
| beta_swarm_prey[5]-0.12 | | -0.12 | 0.03 | 0.03 | -0.17 | -0.07 | 1 | 3800.45 | 3499.44 |
| beta_swarm_prey[6]-0.01 | | -0.01 | 0.02 | 0.02 | -0.05 | 0.03 | 1 | 3938.44 | 3243.25 |
| beta_swarm_prey[7] 0.04 | | 0.04 | 0.03 | 0.03 | -0.01 | 0.08 | 1 | 4013.60 | 2836.02 |
| beta_swarm_prey[8] 0.02 | | 0.02 | 0.04 | 0.04 | -0.04 | 0.08 | 1 | 3963.43 | 3452.43 |
| beta_preyO 0.23 | | 0.24 | 0.27 | 0.27 | -0.24 | 0.66 | 1 | 3476.83 | 3232.61 |
| beta_preyB 0.06 | | 0.06 | 0.15 | 0.15 | -0.18 | 0.31 | 1 | 3566.87 | 3089.49 |
| beta_preyE 0.02 | | 0.04 | 0.34 | 0.31 | -0.57 | 0.53 | 1 | 3130.93 | 3000.10 |
| beta_preyS 0.56 | | 0.57 | 0.20 | 0.20 | 0.24 | 0.89 | 1 | 3109.56 | 2858.44 |
| beta_swarm_prey[1]-0.02 | | -0.02 | 0.03 | 0.03 | -0.06 | 0.03 | 1 | 3402.02 | 3281.40 |
| beta_swarm_prey[2]-0.07 | | -0.07 | 0.03 | 0.03 | -0.12 | -0.02 | 1 | 3561.22 | 3225.42 |
| beta_swarm_prey[3] 0.04 | | 0.04 | 0.02 | 0.02 | 0.01 | 0.07 | 1 | 3916.36 | 3193.65 |
| beta_swarm_prey[4]-0.04 | | -0.04 | 0.02 | 0.02 | -0.07 | 0.00 | 1 | 4105.89 | 3363.66 |
| beta_swarm_prey[5]-0.12 | | -0.12 | 0.03 | 0.03 | -0.17 | -0.07 | 1 | 3800.45 | 3499.44 |
| beta_swarm_prey[6]-0.01 | | -0.01 | 0.02 | 0.02 | -0.05 | 0.03 | 1 | 3938.44 | 3243.25 |
| beta_swarm_prey[7] 0.04 | | 0.04 | 0.03 | 0.03 | -0.01 | 0.08 | 1 | 4013.60 | 2836.02 |
| beta_swarm_prey[8] 0.02 | | 0.02 | 0.04 | 0.04 | -0.04 | 0.08 | 1 | 3963.43 | 3452.43 |
| alpha_swarm[1] 0.26 | | 0.23 | 0.91 | 0.89 | -1.20 | 1.78 | 1 | 3590.10 | 3312.70 |
| alpha_swarm[2] 0.49 | | 0.48 | 0.87 | 0.85 | -0.89 | 1.93 | 1 | 3730.34 | 3201.26 |
| alpha_swarm[3] -0.09 | | -0.10 | 0.89 | 0.87 | -1.54 | 1.39 | 1 | 3702.72 | 3296.10 |
| alpha_swarm[4] -0.12 | | -0.15 | 0.90 | 0.90 | -1.56 | 1.39 | 1 | 3876.32 | 3524.19 |
| alpha_swarm[5] -0.29 | | -0.30 | 0.90 | 0.89 | -1.78 | 1.22 | 1 | 3650.97 | 3324.24 |
| alpha_swarm[6] -0.01 | | -0.02 | 0.89 | 0.88 | -1.52 | 1.43 | 1 | 3721.72 | 3409.34 |
| alpha_swarm[7] -0.22 | | -0.23 | 0.90 | 0.89 | -1.69 | 1.28 | 1 | 3801.18 | 3451.42 |
| alpha_swarm[8] 0.13 | | 0.11 | 0.90 | 0.89 | -1.29 | 1.65 | 1 | 3698.60 | 3278.34 |
| alpha_swarm[9] -0.10 | | -0.11 | 0.89 | 0.87 | -1.55 | 1.40 | 1 | 3824.41 | 3335.36 |
| alpha_swarm[10] 0.07 | | 0.07 | 0.90 | 0.87 | -1.39 | 1.56 | 1 | 3681.38 | 2988.01 |
| alpha_swarm[11] -0.36 | | -0.37 | 0.87 | 0.85 | -1.77 | 1.08 | 1 | 3756.33 | 3098.81 |
| alpha_swarm[12] -0.15 | | -0.17 | 0.90 | 0.88 | -1.60 | 1.38 | 1 | 3675.13 | 3222.82 |
| alpha_swarm[13] 0.13 | | 0.11 | 0.88 | 0.86 | -1.27 | 1.58 | 1 | 3608.89 | 3222.64 |
| alpha_swarm[14] 0.16 | | 0.14 | 0.89 | 0.88 | -1.26 | 1.62 | 1 | 3815.57 | 3204.04 |
| alpha_prey[1] -0.28 | | -0.27 | 0.82 | 0.83 | -1.64 | 1.08 | 1 | 3649.21 | 3275.19 |
| alpha_prey[2] -0.34 | | -0.33 | 0.82 | 0.83 | -1.69 | 0.98 | 1 | 3691.52 | 3203.66 |
| alpha_prey[3] -0.31 | | -0.30 | 0.82 | 0.84 | -1.66 | 1.04 | 1 | 3684.04 | 3175.74 |
| alpha_prey[4] -0.30 | | -0.29 | 0.83 | 0.84 | -1.67 | 1.03 | 1 | 3663.62 | 3188.05 |
| alpha_prey[5] -0.43 | | -0.43 | 0.82 | 0.84 | -1.78 | 0.91 | 1 | 3675.53 | 3415.21 |

| variable | mean | median | sd | mad | q5 | q95 | rhat | ess_bulk | ess_tail |
| --- | --- | --- | --- | --- | --- | --- | --- | --- | --- |
| alpha_prey[6] | -0.13 | -0.13 | 0.85 | 0.86 | -1.54 | 1.26 | 1 | 3526.58 | 3096.25 |
| alpha_prey[7] | -0.34 | -0.33 | 0.82 | 0.83 | -1.70 | 0.99 | 1 | 3620.42 | 3344.75 |
| alpha_prey[8] | -0.35 | -0.32 | 0.82 | 0.83 | -1.71 | 1.00 | 1 | 3660.94 | 3260.26 |
| alpha_prey[9] | -0.41 | -0.40 | 0.82 | 0.82 | -1.77 | 0.94 | 1 | 3674.47 | 3180.75 |
| alpha_prey[10] | -0.42 | -0.41 | 0.82 | 0.83 | -1.77 | 0.89 | 1 | 3661.24 | 3266.78 |
| alpha_prey[11] | -0.31 | -0.30 | 0.82 | 0.83 | -1.66 | 1.02 | 1 | 3665.17 | 3346.40 |
| alpha_prey[12] | -0.37 | -0.37 | 0.82 | 0.83 | -1.71 | 0.98 | 1 | 3722.31 | 3420.38 |
| alpha_prey[13] | -0.51 | -0.51 | 0.82 | 0.82 | -1.84 | 0.83 | 1 | 3647.15 | 3222.99 |
| alpha_prey[14] | -0.31 | -0.30 | 0.82 | 0.84 | -1.68 | 1.01 | 1 | 3669.70 | 3374.58 |
| alphaO[1] | -0.46 | -0.43 | 1.14 | 1.15 | -2.38 | 1.40 | 1 | 3939.50 | 3341.76 |
| alphaO[2] | -0.67 | -0.63 | 1.26 | 1.23 | -2.78 | 1.30 | 1 | 3802.38 | 3131.32 |
| alphaO[3] | 0.24 | 0.23 | 1.11 | 1.11 | -1.61 | 2.07 | 1 | 3815.59 | 3501.34 |
| alphaO[4] | 0.10 | 0.08 | 1.12 | 1.12 | -1.73 | 1.97 | 1 | 3811.34 | 3294.60 |
| alphaO[5] | 0.24 | 0.26 | 1.10 | 1.09 | -1.56 | 2.03 | 1 | 3933.20 | 3537.77 |
| alphaO[6] | 0.48 | 0.47 | 1.12 | 1.10 | -1.41 | 2.32 | 1 | 3829.01 | 3494.34 |
| alphaO[7] | -0.35 | -0.33 | 1.18 | 1.18 | -2.32 | 1.52 | 1 | 4025.04 | 3384.94 |
| alphaO[8] | 0.27 | 0.26 | 1.09 | 1.10 | -1.54 | 2.07 | 1 | 3655.83 | 3304.44 |
| alphaB[1] | 0.71 | 0.68 | 1.06 | 1.05 | -1.05 | 2.47 | 1 | 3659.29 | 3297.80 |
| alphaB[2] | -0.22 | -0.21 | 1.06 | 1.05 | -1.99 | 1.51 | 1 | 3721.02 | 3242.04 |
| alphaB[3] | 0.97 | 0.97 | 1.07 | 1.08 | -0.82 | 2.75 | 1 | 3615.36 | 3059.86 |
| alphaB[4] | 0.07 | 0.04 | 1.05 | 1.03 | -1.66 | 1.80 | 1 | 3678.21 | 3232.01 |
| alphaB[5] | 0.13 | 0.12 | 1.06 | 1.04 | -1.64 | 1.90 | 1 | 3725.36 | 3080.42 |
| alphaB[6] | -0.17 | -0.18 | 1.06 | 1.03 | -1.93 | 1.58 | 1 | 3702.18 | 3032.45 |
| alphaB[7] | -0.86 | -0.86 | 1.08 | 1.08 | -2.65 | 0.92 | 1 | 3611.75 | 3217.67 |
| alphaB[8] | -0.47 | -0.45 | 1.05 | 1.05 | -2.21 | 1.30 | 1 | 3672.04 | 3186.15 |
| alphaE[1] | -0.70 | -0.62 | 1.26 | 1.22 | -2.90 | 1.25 | 1 | 3554.83 | 3201.01 |
| alphaE[2] | 0.12 | 0.10 | 1.13 | 1.10 | -1.71 | 1.97 | 1 | 3719.04 | 3111.20 |
| alphaE[3] | 0.24 | 0.22 | 1.15 | 1.12 | -1.59 | 2.19 | 1 | 3648.87 | 3291.90 |
| alphaE[4] | -0.10 | -0.10 | 1.12 | 1.11 | -1.94 | 1.71 | 1 | 3511.60 | 3259.40 |
| alphaE[5] | 0.41 | 0.38 | 1.14 | 1.11 | -1.46 | 2.32 | 1 | 3698.34 | 3217.71 |
| alphaE[6] | -0.37 | -0.34 | 1.17 | 1.16 | -2.36 | 1.54 | 1 | 3844.37 | 3437.46 |
| alphaE[7] | -0.37 | -0.32 | 1.17 | 1.14 | -2.33 | 1.46 | 1 | 3820.74 | 3380.97 |
| alphaE[8] | 0.60 | 0.60 | 1.16 | 1.15 | -1.26 | 2.59 | 1 | 3612.78 | 3298.31 |
| alphaS[1] | 0.07 | 0.07 | 1.03 | 1.01 | -1.66 | 1.75 | 1 | 3642.70 | 3089.90 |
| alphaS[2] | -0.18 | -0.15 | 1.06 | 1.04 | -1.93 | 1.53 | 1 | 3710.77 | 3339.29 |
| alphaS[3] | 0.02 | 0.03 | 1.03 | 1.01 | -1.70 | 1.69 | 1 | 3791.09 | 3157.03 |
| alphaS[4] | -0.09 | -0.08 | 1.04 | 1.02 | -1.80 | 1.60 | 1 | 3618.26 | 3162.50 |
| alphaS[5] | 0.08 | 0.08 | 1.05 | 1.05 | -1.64 | 1.79 | 1 | 3570.35 | 3157.11 |
| alphaS[6] | 0.08 | 0.07 | 1.04 | 1.01 | -1.61 | 1.83 | 1 | 3678.23 | 3146.04 |
| alphaS[7] | 0.01 | 0.01 | 1.04 | 1.00 | -1.70 | 1.68 | 1 | 3693.18 | 3188.96 |
| alphaS[8] | 0.16 | 0.16 | 1.04 | 1.05 | -1.53 | 1.84 | 1 | 3923.41 | 3350.88 |

Rhat

1.000

1.006

1.012

| variable | mean | median | sd | mad | q5 | q95 | rhat | ess_bulk | ess_tail |
| --- | --- | --- | --- | --- | --- | --- | --- | --- | --- |
| gamma_prey[1] | 0.51 | 0.51 | 0.50 | 0.50 | -0.31 | 1.34 | 1 | 3830.56 | 3331.90 |
| gamma_prey[2] | -0.84 | -0.84 | 0.50 | 0.50 | -1.67 | -0.01 | 1 | 3839.16 | 3331.76 |
| gamma_prey[3] | 1.19 | 1.19 | 0.50 | 0.50 | 0.36 | 2.01 | 1 | 3854.77 | 3331.76 |
| gamma_prey[4] | -0.85 | -0.84 | 0.50 | 0.49 | -1.67 | -0.03 | 1 | 3846.73 | 3294.14 |
| gamma_prey[5] | -0.89 | -0.89 | 0.50 | 0.50 | -1.72 | -0.07 | 1 | 3850.49 | 3370.74 |
| gamma_prey[6] | -0.92 | -0.92 | 0.50 | 0.50 | -1.75 | -0.09 | 1 | 3849.70 | 3331.19 |
| gamma_prey[7] | -0.84 | -0.83 | 0.50 | 0.50 | -1.66 | -0.01 | 1 | 3847.13 | 3219.80 |
| gamma_prey[8] | 0.86 | 0.86 | 0.50 | 0.50 | 0.03 | 1.69 | 1 | 3842.16 | 3256.81 |

## S7.6 Sampling diagnostics

mod_diagnostics(mod, summary_mod)

0	1000	2000	3000	4000

ESS parameters (tail and bulk)

Figure S15: Bulk and tail effective sampling size (ESS) of the model assessing the interrelated processes affecting the space use by the ant-followers assemblage

ppcheck_OCAN <- mod$draws('ppcheckO', format = 'matrix')

par(mfrow = c(2, 3), mar = c(4, 4, 1, 1)) plot(density(dat_generative1$OCAN), main = '', xlab = 'BIAN',

ylim = c(0, 10), lwd = 0)

**for** (i **in** 1:100) lines(density(ppcheck_OCAN[i, ]), lwd = 0.1) lines(density(dat_generative1$OCAN), col = 'red', lwd = 2)

ppcheck_BIAN <- mod$draws('ppcheckB', format = 'matrix') plot(density(dat_generative1$BIAN), main = '', xlab = 'BIAN',

ylim = c(0, 4), lwd = 0)

**for** (i **in** 1:100) lines(density(ppcheck_BIAN[i, ]), lwd = 0.1) lines(density(dat_generative1$BIAN), col = 'red', lwd = 2)

ppcheck_GHET <- mod$draws('ppcheckE', format = 'matrix') plot(density(dat_generative1$GHET), main = '', xlab = 'GHET',

ylim = c(0, 15), lwd = 0)

**for** (i **in** 1:100) lines(density(ppcheck_GHET[i, ]), lwd = 0.1) lines(density(dat_generative1$GHET), col = 'red', lwd = 2)

ppcheck_SPAN <- mod$draws('ppcheckS', format = 'matrix') plot(density(dat_generative1$SPAN), main = '',

xlab = 'SPAN', ylim = c(0, 15), lwd = 0)

**for** (i **in** 1:100) lines(density(ppcheck_SPAN[i, ]), lwd = 0.1) lines(density(dat_generative1$SPAN), col = 'red', lwd = 2)

ppcheck_swarm <- mod$draws('ppcheck_swarm', format = 'matrix') plot(density(dat_generative1$z_swarm_area), ylim = c(0, 0.8), main = '',

xlab = 'Swarm area')

**for** (i **in** 1:100) lines(density(ppcheck_swarm[i, ]), lwd = 0.1) lines(density(dat_generative1$z_swarm_area), col = 'red', lwd = 3)

ppcheck_prey <- mod$draws('ppcheck_prey', format = 'matrix') plot(density(dat_generative1$z_prey_biomass), ylim = c(0, 0.8),

main = '', xlab = 'Prey biomass')

**for** (i **in** 1:500) lines(density(ppcheck_prey[i, ]), lwd = 0.1) lines(density(dat_generative1$z_prey_biomass), col = 'red', lwd = 3)

0.0	0.4	0.8

Density

0

2	4	6	8

10

Density

0

1

2

3

4

Density

0.8

0

5

10

15

0.0	0.5	1.0

0.0	0.4	0.8

BIAN

Density

10

15

0.0	0.4	0.8

0

5

BIAN

−2	0	2	4

Density

0.0

0.2 0.4 0.6

0.8

GHET

−2	0	2	4	6

SPAN

Density

0.0

0.2 0.4 0.6

Swarm area

Prey biomass

Figure S16: Posterior predictive checks of the model assessing the interrelated processes af-fecting the space use by the ant-followers assemblage

par(mfrow = c(1, 1))

## S7.7 Posterior distributions

In this section we extract the posterior distributions and used the structure of the model to plot and estimate conditional effects, and conduct simulations to assess the effect of dominance.

post <- mod$draws(pars, format = 'df')

post <-

lapply(pars, FUN =

**function**(x) {

post[grep(x, names(post))]

})

Warning: Dropping 'draws_df' class as required metadata was removed. Warning: Dropping 'draws_df' class as required metadata was removed. Warning: Dropping 'draws_df' class as required metadata was removed. Warning: Dropping 'draws_df' class as required metadata was removed. Warning: Dropping 'draws_df' class as required metadata was removed. Warning: Dropping 'draws_df' class as required metadata was removed.

Warning: Dropping 'draws_df' class as required metadata was removed. Warning: Dropping 'draws_df' class as required metadata was removed. Warning: Dropping 'draws_df' class as required metadata was removed. Warning: Dropping 'draws_df' class as required metadata was removed. Warning: Dropping 'draws_df' class as required metadata was removed. Warning: Dropping 'draws_df' class as required metadata was removed. Warning: Dropping 'draws_df' class as required metadata was removed. Warning: Dropping 'draws_df' class as required metadata was removed. Warning: Dropping 'draws_df' class as required metadata was removed. Warning: Dropping 'draws_df' class as required metadata was removed. Warning: Dropping 'draws_df' class as required metadata was removed. Warning: Dropping 'draws_df' class as required metadata was removed. Warning: Dropping 'draws_df' class as required metadata was removed. Warning: Dropping 'draws_df' class as required metadata was removed. Warning: Dropping 'draws_df' class as required metadata was removed. Warning: Dropping 'draws_df' class as required metadata was removed. Warning: Dropping 'draws_df' class as required metadata was removed. Warning: Dropping 'draws_df' class as required metadata was removed. Warning: Dropping 'draws_df' class as required metadata was removed. Warning: Dropping 'draws_df' class as required metadata was removed. Warning: Dropping 'draws_df' class as required metadata was removed. Warning: Dropping 'draws_df' class as required metadata was removed. Warning: Dropping 'draws_df' class as required metadata was removed. Warning: Dropping 'draws_df' class as required metadata was removed. Warning: Dropping 'draws_df' class as required metadata was removed. Warning: Dropping 'draws_df' class as required metadata was removed. Warning: Dropping 'draws_df' class as required metadata was removed. Warning: Dropping 'draws_df' class as required metadata was removed. Warning: Dropping 'draws_df' class as required metadata was removed. Warning: Dropping 'draws_df' class as required metadata was removed. Warning: Dropping 'draws_df' class as required metadata was removed. Warning: Dropping 'draws_df' class as required metadata was removed. Warning: Dropping 'draws_df' class as required metadata was removed. Warning: Dropping 'draws_df' class as required metadata was removed. Warning: Dropping 'draws_df' class as required metadata was removed. Warning: Dropping 'draws_df' class as required metadata was removed. Warning: Dropping 'draws_df' class as required metadata was removed. Warning: Dropping 'draws_df' class as required metadata was removed. Warning: Dropping 'draws_df' class as required metadata was removed. Warning: Dropping 'draws_df' class as required metadata was removed.

names(post) <- pars

### S7.7.1 Effect of swarm area on ant-follower species

t1 <- Sys.time()

swarm_effect <-lapply(1, FUN =

**function**(z) {

x_seq <- seq(min(dat_generative1$z_swarm_area), max(dat_generative1$z_swarm_area), length.out = 100)

mS <-

sapply(x_seq, FUN =

**function**(i) { v <-

with(post,

{

apply(alphaS, 1, mean) + apply(thetaS, 1, mean) + beta_swarmS[[1]] * i + beta_preyS[[1]] * 0 + apply(beta_OCAN_S, 1, mean) * 0 +

apply(beta_GHET_S, 1, mean) * 0 +

apply(beta_BIAN_S, 1, mean) * 0 + apply(tauS, 1, mean) + apply(psiS, 1, mean) + apply(deltaS, 1, mean)

})

inv_logit(v)

})

mS <-

do.call('rbind',

apply(mS, 2, FUN =

**function**(j) { tibble(mu = mean(j),

li = quantile(j, 0.025), ls = quantile(j, 0.975))

}, simplify = 'list'))

mS$pr <- mean(post$beta_swarmS[[1]] > 0) mS$beta_mu <- mean(post$beta_swarmS[[1]]) mS$beta_sd <- sd(post$beta_swarmS[[1]]) mS$x <- x_seq

mS$spp <- 'H. naevioides'

mB <-

sapply(x_seq, FUN =

**function**(i) { v <-

with(post,

{

apply(alphaB, 1, mean) + apply(thetaB, 1, mean) + beta_swarmB[[1]] * i + beta_preyB[[1]] * 0 + apply(beta_OCAN_B, 1, mean) * 0 +

apply(beta_GHET_B, 1, mean) * 0 + apply(tauB, 1, mean) +

apply(psiB, 1, mean) + apply(deltaB, 1, mean)

})

mB <-

inv_logit(v)

})

do.call('rbind',

apply(mB, 2, FUN =

**function**(j) { tibble(mu = mean(j),

li = quantile(j, 0.025), ls = quantile(j, 0.975))

}, simplify = 'list'))

mB$pr <- mean(post$beta_swarmB[[1]] > 0) mB$beta_mu <- mean(post$beta_swarmB[[1]]) mB$beta_sd <- sd(post$beta_swarmB[[1]]) mB$x <- x_seq

mB$spp <- 'G. bicolor '

mE <-

sapply(x_seq, FUN =

**function**(i) { v <-

with(post,

{

apply(alphaE, 1, mean) + apply(thetaE, 1, mean) + beta_swarmE[[1]] * i + beta_preyE[[1]] * 0 + apply(beta_OCAN_E, 1, mean) * 0 + apply(tauE, 1, mean) + apply(psiE, 1, mean) + apply(deltaE, 1, mean)

})

mE <-

inv_logit(v)

})

do.call('rbind',

apply(mE, 2, FUN =

**function**(j) { tibble(mu = mean(j),

li = quantile(j, 0.025), ls = quantile(j, 0.975))

}, simplify = 'list'))

mE$pr <- mean(post$beta_swarmE[[1]] > 0) mE$beta_mu <- mean(post$beta_swarmE[[1]]) mE$beta_sd <- sd(post$beta_swarmE[[1]]) mE$x <- x_seq

mE$spp <- 'E. penicillata'

mO <-

sapply(x_seq, FUN =

**function**(i) { v <-

with(post,

{

apply(alphaO, 1, mean) + apply(thetaO, 1, mean) + beta_swarmO[[1]] * i + beta_preyO[[1]] * 0 + apply(tauO, 1, mean) + apply(psiO, 1, mean) + apply(deltaO, 1, mean)

})

})

inv_logit(v)

mO <-

do.call('rbind',

apply(mO, 2, FUN =

**function**(j) { tibble(mu = mean(j),

li = quantile(j, 0.025), ls = quantile(j, 0.975))

}, simplify = 'list'))

mO$pr <- mean(post$beta_swarmO[[1]] > 0) mO$beta_mu <- mean(post$beta_swarmO[[1]]) mO$beta_sd <- sd(post$beta_swarmO[[1]]) mO$x <- x_seq

mO$spp <- 'P. mcleannani'

rbind(mS, mB, mE, mO)

Sys.time() - t1

})

Time difference of 22.69759 secs

\# S Span, b bian, E ghet, O Ocan

swarm_effect <-

do.call('rbind', swarm_effect)

plot_swarm_effect <-

lapply(seq_along(unique(swarm_effect$spp)), FUN =

**function**(x) {

d <- unique(swarm_effect[, c('pr', "spp")])

swarm_effect[swarm_effect$spp == d$spp[x], ] |> ggplot(aes(x, mu, ymin = li, ymax = ls)) + geom_line() +

geom_ribbon(alpha = 0.5) + annotate(geom = 'text',

label = paste('Pr(slope > 0) = ', round(d$pr[x], 3)), x = 1, y = 0.1) +

facet_wrap(~spp, scales = 'free') + labs(x = 'Swarm area (z-scores)',

y = 'Pr(observation)')

})

plot_grid(plot_swarm_effect[[1]],

plot_swarm_effect[[2]], plot_swarm_effect[[3]], plot_swarm_effect[[4]], ncol = 2)

0.20

Pr(observation)

0.15

0.10

0.05

0.00

0	2	4

Swarm area (z−scores)

0.8

0.6

H. naevioides

Pr(slope > 0) = 0.601

Pr(observation)

0.4

0.2

0	2	4

G. bicolor

Pr(slope > 0) = 1

Swarm area (z−scores)

0.100

Pr(observation)

0.075

0.050

0.025

0.000

0	2	4

Swarm area (z−scores)

0.4

0.3

E. penicillata

Pr(slope > 0) = 0.295

Pr(observation)

0.2

0.1

0.0

0	2	4

P. mcleannani

Pr(slope > 0) = 0.999

Swarm area (z−scores)

Figure S17: Effect of swarm area on the probability of observing ant-follower species

### S7.7.2 Effect of profitability on ant-followers presence

t1 <- Sys.time()

prey_effect <-lapply(1, FUN =

**function**(z) {

x_seq <- seq(min(dat_generative1$z_prey_biomass), max(dat_generative1$z_prey_biomass), length.out = 100)

mS <-

sapply(x_seq, FUN =

**function**(i) { v <-

with(post,

{

apply(alphaS, 1, mean) + apply(thetaS, 1, mean) + beta_swarmS[[1]] * 0 + beta_preyS[[1]] * i + apply(beta_OCAN_S, 1, mean) * 0 +

apply(beta_GHET_S, 1, mean) * 0 +

apply(beta_BIAN_S, 1, mean) * 0 + apply(tauS, 1, mean) + apply(psiS, 1, mean) + apply(deltaS, 1, mean)

})

inv_logit(v)

})

mS <-

do.call('rbind',

apply(mS, 2, FUN =

**function**(j) { tibble(mu = mean(j),

li = quantile(j, 0.025), ls = quantile(j, 0.975))

}, simplify = 'list'))

mS$pr <- mean(post$beta_preyS[[1]] > 0) mS$beta_mu <- mean(post$beta_preyS[[1]]) mS$beta_sd <- sd(post$beta_preyS[[1]]) mS$x <- x_seq

mS$spp <- 'H. naevioides'

mB <-

sapply(x_seq, FUN =

**function**(i) { v <-

with(post,

{

apply(alphaB, 1, mean) + apply(thetaB, 1, mean) + beta_swarmB[[1]] * 0 + beta_preyB[[1]] * i + apply(beta_OCAN_B, 1, mean) * 0 +

apply(beta_GHET_B, 1, mean) * 0 + apply(tauB, 1, mean) + apply(psiB, 1, mean) + apply(deltaB, 1, mean)

})

mB <-

inv_logit(v)

})

do.call('rbind',

apply(mB, 2, FUN =

**function**(j) { tibble(mu = mean(j),

li = quantile(j, 0.025), ls = quantile(j, 0.975))

}, simplify = 'list'))

mB$pr <- mean(post$beta_preyB[[1]] > 0) mB$beta_mu <- mean(post$beta_preyB[[1]]) mB$beta_sd <- sd(post$beta_preyB[[1]]) mB$x <- x_seq

mB$spp <- 'G. bicolor '

mE <-

sapply(x_seq, FUN =

**function**(i) { v <-

with(post,

{

apply(alphaE, 1, mean) + apply(thetaE, 1, mean) + beta_swarmE[[1]] * 0 + beta_preyE[[1]] * i + apply(beta_OCAN_E, 1, mean) * 0 + apply(tauE, 1, mean) + apply(psiE, 1, mean) + apply(deltaE, 1, mean)

})

inv_logit(v)

})

mE <-

do.call('rbind',

apply(mE, 2, FUN =

**function**(j) { tibble(mu = mean(j),

li = quantile(j, 0.025), ls = quantile(j, 0.975))

}, simplify = 'list'))

mE$pr <- mean(post$beta_preyE[[1]] > 0) mE$beta_mu <- mean(post$beta_preyE[[1]]) mE$beta_sd <- sd(post$beta_preyE[[1]]) mE$x <- x_seq

mE$spp <- 'E. penicillata'

mO <-

sapply(x_seq, FUN =

**function**(i) { v <-

with(post,

{

apply(alphaO, 1, mean) + apply(thetaO, 1, mean) + beta_swarmO[[1]] * 0 + beta_preyO[[1]] * i + apply(tauO, 1, mean) + apply(psiO, 1, mean) + apply(deltaO, 1, mean)

})

inv_logit(v)

})

mO <-

do.call('rbind',

apply(mO, 2, FUN =

**function**(j) { tibble(mu = mean(j),

li = quantile(j, 0.025), ls = quantile(j, 0.975))

}, simplify = 'list'))

mO$pr <- mean(post$beta_preyO[[1]] > 0) mO$beta_mu <- mean(post$beta_preyO[[1]]) mO$beta_sd <- sd(post$beta_preyO[[1]]) mO$x <- x_seq

mO$spp <- 'P. mcleannani'

rbind(mS, mB, mE, mO)

Sys.time() - t1

})

Time difference of 23.21592 secs

\# S Span, b bian, E ghet, O Ocan

prey_effect <-

do.call('rbind', prey_effect)

plot_prey_effect <-

lapply(seq_along(unique(prey_effect$spp)), FUN =

**function**(x) {

d <- unique(prey_effect[, c('pr', "spp")])

prey_effect[prey_effect$spp == d$spp[x], ] |> ggplot(aes(x, mu, ymin = li, ymax = ls)) + geom_line() +

geom_ribbon(alpha = 0.5) + annotate(geom = 'text',

label = paste('Pr(slope > 0) = ', round(d$pr[x], 3)), x = 1, y = 0.1) +

facet_wrap(~spp, scales = 'free') + labs(x = 'Prey biomass (z-scores)',

y = 'Pr(observation)')

})

plot_grid(plot_prey_effect[[1]],

plot_prey_effect[[2]], plot_prey_effect[[3]], plot_prey_effect[[4]], ncol = 2)

0.6

Pr(observation)

0.4

0.2

0.0

0	2	4

Prey biomass (z−scores)

0.6

0.4

H. naevioides

Pr(slope > 0) = 0.994

Pr(observation)

0.2

0	2	4

G. bicolor

Pr(slope > 0) = 0.666

Prey biomass (z−scores)

0.15	0.15

Pr(slope > 0) = 0.556

E. penicillata

P. mcleannani

Pr(slope > 0) = 0.803

Pr(observation)

Pr(observation)

0.10	0.10

0.05	0.05

0.00

0	2	4

Prey biomass (z−scores)

0.00

0	2	4

Prey biomass (z−scores)

Figure S18: Effect of prey biomass on the probability of observing ant-follower species

### S7.7.3 Effect of dominance on the space use by ant followers

To explore the effect of dominance on the space use by each species, we use the model to design two types of interventions: (i) *species i* alone at the *swarm section j*, and (ii) *species i* with *N dominant species* at the *swarm section j*. To conduct the interventions I conditioned the effect of swarm area and prey biomass on average values.

Since we have little data for *P. mcleannani*, *E. penicillata* and *H. naevioides*, the coeﬀicients are very variable, and we can not come to any conclusion regarding the use of the sections across the swarm. The only thing that we can say with some confidence is that observing any of those species was very rare (𝑃 𝑟(𝑝𝑟𝑒𝑠𝑒𝑛𝑐𝑒) < 1%) (See the conditional plots below).

S7.7.3.1 𝑃 . 𝑚𝑐𝑙𝑒𝑎𝑛𝑛𝑎𝑛𝑖 space use

response_OCAN <-

lapply(1, FUN =

**function**(x) {

OCAN <-

lapply(seq_along(post$alphaO), FUN =

**function**(E) {

cat <- which(group_codes$categories$code == E) cat <- group_codes$categories$label[cat]

indx <- which(dat_generative1$categories == E) psi <- unique(dat_generative1$site[indx])

delta <- unique(dat_generative1$swarm_ID[indx]) theta <- unique(dat_generative1$date[indx])

tau <- unique(dat_generative1$round_number[indx])

p <- with(post,

{

alphaO[[E]] + beta_swarmO[[1]] * 0 + beta_preyO[[1]] * 0 +

apply(psiO[, psi], 1, mean) + apply(deltaO[, delta], 1, mean) + apply(thetaO[, theta], 1, mean) + apply(tauO[, tau], 1, mean)

})

p <- inv_logit(p)

})

tibble(mu = c(mean(p)),

li = c(quantile(p, 0.025)), ls = c(quantile(p, 0.975)), location = cat)

OCAN <- do.call('rbind', OCAN) OCAN

})

response_OCAN <- do.call('rbind', response_OCAN)

response_OCAN |>

ggplot(aes(fct_reorder(location, mu), mu, ymin = li, ymax = ls)) +

geom_point(position = position_dodge(width = 0.4)) + geom_errorbar(width = 0,

position = position_dodge(width = 0.4)) + labs(x = 'Location at the swarm',

y = 'Pr(observing P. mcleannani)')

0.02

Pr(observing P. mcleannani)

0.01

0.00

C	BP	O	IB	IF	FP	SP	IS

Location at the swarm

Figure S19: Probability of P. mcleannani using diferent sections within and outside the swarm

S7.7.3.2 𝐸. 𝑝𝑒𝑛𝑖𝑐𝑖𝑙𝑙𝑎𝑡𝑎 space use

response_GHET <-

lapply(1, FUN =

**function**(x) {

GHET <-

lapply(seq_along(post$alphaE), FUN =

**function**(E) {

cat <- which(group_codes$categories$code == E) cat <- group_codes$categories$label[cat]

indx <- which(dat_generative1$categories == E) psi <- unique(dat_generative1$site[indx])

delta <- unique(dat_generative1$swarm_ID[indx]) theta <- unique(dat_generative1$date[indx])

tau <- unique(dat_generative1$round_number[indx])

p <- with(post,

{

alphaE[[E]] + beta_swarmE[[1]] * 0 + beta_preyE[[1]] * 0 + beta_OCAN_E[[E]] * 1 +

apply(psiE[, psi], 1, mean) + apply(deltaE[, delta], 1, mean) + apply(thetaE[, theta], 1, mean) + apply(tauE[, tau], 1, mean)

})

p <- inv_logit(p)

p2 <- with(post,

{

alphaE[[E]] + beta_swarmE[[1]] * 0 + beta_preyE[[1]] * 0 + beta_OCAN_E[[E]] * 0 +

apply(psiE[, psi], 1, mean) + apply(deltaE[, delta], 1, mean) + apply(thetaE[, theta], 1, mean) + apply(tauE[, tau], 1, mean)

})

p2 <- inv_logit(p2)

})

tibble(mu = c(mean(p), mean(p2)), li = c(quantile(p, 0.025),

quantile(p2, 0.025)), ls = c(quantile(p, 0.975),

quantile(p2, 0.975)), location = cat,

intervention = c('P. mcleannani present',

'P. mcleannani absent'))

GHET <- do.call('rbind', GHET) GHET

})

response_GHET <- do.call('rbind', response_GHET)

response_GHET |>

ggplot(aes(fct_reorder(location, mu), mu, ymin = li, ymax = ls, color = intervention)) +

geom_point(position = position_dodge(width = 0.4)) + geom_errorbar(width = 0,

position = position_dodge(width = 0.4)) + labs(x = 'Location at the swarm',

y = 'Pr(observing E. penicillata)')

0.06

0.04

Pr(observing E. penicillata)

0.02

0.00

BP	IS	O	IB	C	FP	IF	SP

Location at the swarm

intervention

P. mcleannani absent

P. mcleannani present

Figure S20: Probability of E. penicillata using diferent sections within and outside the swarm considering diferent scenarios of potential competition

S7.7.3.3 𝐺. 𝑏𝑖𝑐𝑜𝑙𝑜𝑟 space use

response_BIAN <-

lapply(1:4, FUN =

**function**(z) {

com <- c('No dominant spp.',

'Only P. mcleannani', 'Only E. penicillata', 'Two dominant spp.')

est <- list(c(0, 0),

c(1, 0),

c(0, 1),

c(1, 1))

l <-

lapply(seq_along(post$alphaB), FUN =

**function**(x) {

cat <- which(group_codes$categories$code == x) cat <- group_codes$categories$label[cat]

indx <- which(dat_generative1$categories==x) indx_theta <- unique(dat_generative1$date[indx]) indx_psi <- unique(dat_generative1$site[indx]) indx_delta <- unique(dat_generative1$swarm_ID[indx]) indx_tau <- unique(dat_generative1$round_number[x])

v <-

})

with(post,

{

alphaB[[x]] +

apply(thetaB[, indx_theta], 1, mean) + beta_swarmB[[1]] * 0 +

beta_preyB[[1]] * 0 + beta_OCAN_B[[x]] * est[[z]][1] +

beta_GHET_B[[x]] * est[[z]][2] + apply(tauB[, indx_tau], 1, mean) + apply(psiB[, indx_psi], 1, mean) + apply(deltaB[, indx_delta], 1, mean)

}) |> inv_logit() tibble(location = cat,

mu = mean(v),

li = quantile(v, 0.025), ls = quantile(v, 0.975))

})

l <- do.call('rbind', l) l$intervention <- com[z] l

response_BIAN <- do.call('rbind', response_BIAN)

response_BIAN$location <- factor(response_BIAN$location,

levels = levels(produ_space_use$category))

response_BIAN |>

ggplot(aes(location, mu, ymin = li,

ymax = ls, color = intervention)) + geom_point(position = position_dodge(width = 0.5)) + geom_errorbar(width = 0,

position = position_dodge(width = 0.5)) + labs(x = 'Location at the swarm',

y = 'Pr(observing G. bicolor)') + theme(legend.position = 'top')

rvention	No dominant spp.	Only E. penicillata	Only P. mcleannani	Two d

0.6

Pr(observing G. bicolor)

0.4

0.2

0.0

IF	IS	IB	O	C	BP	SP	FP

Location at the swarm

levels(response_BIAN$location)

[1] "IF" "IS" "IB" "O"	"C"	"BP" "SP" "FP"

response_BIAN |>

ggplot(aes(location, mu,

ymin = li, ymax = ls, color = intervention)) +

geom_errorbar(width = 0,

position = position_dodge(width = 0.4), linewidth = 1.5, alpha = 0.5) +

geom_point(position = position_dodge(width = 0.4), size = 2) +

scale_color_manual(values = c('#4F4C5D', '#8EBAA1',

'#E4AB66', '#D9631E')) +

scale_x_discrete(limits = levels(response_BIAN$location), labels =

c("Outside",

'Interior \nfront ', 'Interior \nside ', 'Center',

'Interior \nback ', 'Back \nperimeter', 'Side \nperimeter', 'Front \nperimeter')) +

theme_classic() +

labs(y = expression('Probability of use by '~italic('G. bicolor')), x = 'Location within and outside the swarm') +

theme(panel.grid = element_blank(), legend.title = element_blank(), legend.position = c(0.15, 0.85),

#legend.background = element_rect(fill = '#f9f4da'),

strip.background = element_blank(), axis.line = element_line(linewidth = 0.25), axis.ticks = element_line(linewidth = 0.25),

#axis.text.x = element_text(angle = 45, hjust = 1, vjust = 1), axis.text = element_text(size = 8.5),

#title = element_blank(), legend.margin = margin(c(0, 0, 0, 0)))

0.6

No dominant spp. Only E. penicillata Only P. mcleannani Two dominant spp.

Probability of use by *G. bicolor*

0.4

0.2

0.0

Outside	Interior

Interior

Center	Interior

Back

Side

Front

front

side

back

perimeter perimeter perimeter

Location within and outside the swarm

Figure S21: Probability of G. bicolor using diferent sections within and outside the swarm considering diferent scenarios of potential competition

S7.7.3.3.1 Contrast when 𝐺. 𝑏𝑖𝑐𝑜𝑙𝑜𝑟 is alone

BIAN_est <-

lapply(1:4, FUN =

**function**(z) {

com <- c('No dominant spp.',

'Only P. mcleannani', 'Only E. penicillata', 'Two dominant spp.')

est <- list(c(0, 0),

c(1, 0),

c(0, 1),

c(1, 1))

l <-

lapply(seq_along(post$alphaB), FUN =

**function**(x) {

cat <- which(group_codes$categories$code == x) cat <- group_codes$categories$label[cat]

indx <- which(dat_generative1$categories==x) indx_theta <- unique(dat_generative1$date[indx]) indx_psi <- unique(dat_generative1$site[indx]) indx_delta <- unique(dat_generative1$swarm_ID[indx]) indx_tau <- unique(dat_generative1$round_number[x])

v <-with(post,

{

alphaB[[x]] +

apply(thetaB[, indx_theta], 1, mean) + beta_swarmB[[1]] * 0 +

beta_preyB[[1]] * 0 + beta_OCAN_B[[x]] * est[[z]][1] +

beta_GHET_B[[x]] * est[[z]][2] + apply(tauB[, indx_tau], 1, mean) + apply(psiB[, indx_psi], 1, mean) + apply(deltaB[, indx_delta], 1, mean)

}) |> inv_logit() tibble(location = cat,

est = v)

})

l <- do.call('rbind', l) l$intervention <- com[z] l

})

BIAN_est <- do.call('rbind', BIAN_est)

cats <- unique(BIAN_est[, c("location", "intervention")]) BIAN_fun <-

**function**(location1, intervention1,

location2, intervention2, compare = T, statistics = T) {

**if** (compare) {

df <- BIAN_est[BIAN_est$location == location1 &

BIAN_est$intervention == intervention1, ]

df1 <- BIAN_est[BIAN_est$location == location2 &

BIAN_est$intervention == intervention2, ]

**if** (statistics) {

d <- tribble(~location, ~intervention, ~mu,	~li,	~ls,

location1, intervention1, mean(df$est), quantile(df$est, 0.025), quantile(df$est, 0.975), location2, intervention2, mean(df1$est), quantile(df1$est, 0.025), quantile(df1$est, 0.975)

d$Pr1_Pr2 <- mean(df$est > df1$est) d

} **else** {

z <- ((df$est-df1$est)/df1$est)*100 tibble(mu = mean(z),

lower_CI = quantile(z, 0.025), upper_CI = quantile(z, 0.975),

Pr_higher = round(mean(df$est > df1$est), 3), comparison = paste('Probability increase (%) from',

location2, 'to', location1), intervention = intervention1)

}

} **else** {

df <- BIAN_est[BIAN_est$location == location1 &

BIAN_est$intervention == intervention1, ]

df

}

}

interventions <- unique(BIAN_est$intervention) table1 <-

**function**(x) {

BIAN_fun(location1 = "FP", intervention1 = interventions[1], location2 = x, intervention2 = interventions[1], compare = T, statistics = F)

})

table1 <- do.call('rbind', table1)

lapply(group_codes$categories$label, FUN =

knitr::kable(table1[-3, ],

digits = 3,

caption = "Contrast of probabilities (in percentage) between FP and each of the other sections (col

Table S8: Contrast of probabilities (in percentage) between FP and each of the other sections (column mu and its credibility intervals: lower_CI - upper_CI)

mu	lower_CI upper_CI Pr_higher comparison	intervention

| 18.212 | -16.336 | 63.934 | 0.823 | Probability increase (%) from BP | No dominant |
| --- | --- | --- | --- | --- | --- |
|  |  |  |  | to FP | spp. |
| 137.706 | 31.689 | 310.767 | 0.998 | Probability increase (%) from C | No dominant |
|  |  |  |  | to FP | spp. |
| 88.097 | 13.550 | 210.349 | 0.993 | Probability increase (%) from IB | No dominant |
|  |  |  |  | to FP | spp. |
| 78.597 | 6.009 | 188.414 | 0.988 | Probability increase (%) from IF | No dominant |
|  |  |  |  | to FP | spp. |
| 126.626 | 32.676 | 290.554 | 0.999 | Probability increase (%) from IS | No dominant |
|  |  |  |  | to FP | spp. |
| 320.084 | 94.152 | 734.371 | 1.000 | Probability increase (%) from O | No dominant |
|  |  |  |  | to FP | spp. |
| 186.862 | 78.557 | 361.448 | 1.000 | Probability increase (%) from SP | No dominant |
|  |  |  |  | to FP | spp. |

S7.7.3.3.2 Contrast when 𝐺. 𝑏𝑖𝑐𝑜𝑙𝑜𝑟 is with other species

interventions <- interventions[c(1, 3, 2, 4)]

table2 <-

lapply(c('IF', 'IB', 'FP', 'BP'), FUN =

**function**(j) {

tau <- lapply(interventions, FUN =

**function**(x) {

BIAN_fun(location1 = j, intervention1 = interventions[1], location2 = j, intervention2 = x,

compare = T, statistics = F)

})

tau <- do.call('rbind', tau) tau$intervention <- interventions tau$swarm_section <- j

tau[-1, -5]

})

table2 <- do.call('rbind', table2)

section i under

knitr::kable(table2,

digits = 3,

caption = "Contrast of probabilities (in percentage) of G. bicolor using the swarm

Table S9: Contrast of probabilities (in percentage) of G. bicolor using the swarm section i under different scenarios of potential competition.

| mu | lower_CI | upper_CI | Pr_higher | intervention | swarm_section |
| --- | --- | --- | --- | --- | --- |
| 30.388 | -36.143 | 163.219 | 0.694 | Only E. penicillata | IF |
| 45.904 | -29.846 | 184.747 | 0.797 | Only P. mcleannani | IF |
| 95.618 | -37.339 | 435.339 | 0.826 | Two dominant spp. | IF |
| 17.314 | -41.996 | 138.286 | 0.586 | Only E. penicillata | IB |
| 42.112 | -32.149 | 190.772 | 0.767 | Only P. mcleannani | IB |
| 68.441 | -44.943 | 348.699 | 0.741 | Two dominant spp. | IB |
| 36.310 | -19.969 | 140.853 | 0.816 | Only E. penicillata | FP |
| 85.854 | 1.676 | 238.106 | 0.979 | Only P. mcleannani | FP |
| 177.251 | 4.644 | 574.694 | 0.982 | Two dominant spp. | FP |
| 6.972 | -40.532 | 97.805 | 0.489 | Only E. penicillata | BP |
| 29.755 | -31.681 | 151.313 | 0.718 | Only P. mcleannani | BP |
| 40.527 | -42.698 | 262.016 | 0.636 | Two dominant spp. | BP |

S7.7.3.4 𝐻. 𝑛𝑎𝑒𝑣𝑖𝑜𝑖𝑑𝑒𝑠 space use

response_SPAN <-

lapply(1:8, FUN =

**function**(z) {

com <- c('No dominant spp.',

'Only P. mcleannani', 'Only E. penicillata', 'Only G. bicolor',

'P. mcleannani & E. penicillata', 'P. mcleannani & G. bicolor',

'E. penicillata & G. bicolor',

'Three dominant spp.')

est <- list(c(0, 0, 0),

c(1, 0, 0),

c(0, 1, 0),

c(0, 0, 1),

c(1, 1, 0),

c(1, 0, 1),

c(0, 1, 1),

c(1, 1, 1))

l <-

})

**function**(x) {

cat <- which(group_codes$categories$code == x) cat <- group_codes$categories$label[cat]

indx <- which(dat_generative1$categories==x) indx_theta <- unique(dat_generative1$date[indx]) indx_psi <- unique(dat_generative1$site[indx]) indx_delta <- unique(dat_generative1$swarm_ID[indx]) indx_tau <- unique(dat_generative1$round_number[x])

v <-with(post,

{

alphaS[[x]] +

apply(thetaS[, indx_theta], 1, mean) + beta_swarmS[[1]] * 0 +

beta_preyS[[1]] * 0 + beta_OCAN_S[[x]] * est[[z]][1] +

beta_GHET_S[[x]] * est[[z]][2] +

beta_BIAN_S[[x]] * est[[z]][3] + apply(tauS[, indx_tau], 1, mean) + apply(psiS[, indx_psi], 1, mean) + apply(deltaS[, indx_delta], 1, mean)

}) |> inv_logit() tibble(location = cat,

mu = mean(v),

li = quantile(v, 0.025), ls = quantile(v, 0.975))

})

l <- do.call('rbind', l) l$intervention <- com[z] l

lapply(seq_along(post$alphaB), FUN =

response_SPAN <- do.call('rbind', response_SPAN)

response_SPAN$intervention <- as.factor(response_SPAN$intervention) response_SPAN$intervention <- factor(response_SPAN$intervention,

levels =

c('No dominant spp.', 'Only P. mcleannani', 'Only E. penicillata', 'Only G. bicolor',

'P. mcleannani & E. penicillata', 'P. mcleannani & G. bicolor',

'E. penicillata & G. bicolor', 'Three dominant spp.'))

response_SPAN |>

ggplot(aes(fct_reorder(location, mu), mu, ymin = li, ymax = ls, color = intervention)) +

geom_point(position = position_dodge(width = 0.7)) + geom_errorbar(width = 0,

position = position_dodge(width = 0.7)) + labs(x = 'Location at the swarm',

y = 'Pr(observing H. naevioides)') + theme(legend.position = 'top')

 No dominant spp.

n

Only P. mcleannani

 Only E. penicillata Only G. bicolor

P. mcleannani & E. penicillata E.

P. mcleannani & G. bicolor	Th

0.100

Pr(observing H. naevioides)

0.075

0.050

0.025

0.000

FP	C	BP	IB	IF	SP	O	IS

Location at the swarm

Figure S22: Probability of H. naevioides using diferent sections within and outside the swarm considering diferent scenarios of potential competition

# S8 Computational envionment

sessionInfo()

R version 4.5.1 (2025-06-13)

Platform: aarch64-apple-darwin20 Running under: macOS Sequoia 15.5

Matrix products: default

BLAS:	/Library/Frameworks/R.framework/Versions/4.5-arm64/Resources/lib/libRblas.0.dylib	LAPACK: /Library/Frameworks/R.framework/Versions/4.5-arm64/Resources/lib/libRlapack.dylib;

locale:

[1] en_US.UTF-8/en_US.UTF-8/en_US.UTF-8/C/en_US.UTF-8/en_US.UTF-8

time zone: America/Sao_Paulo tzcode source: internal

attached base packages:

[1] parallel	stats	graphics	grDevices utils	datasets	methods

[8] base

other attached packages:

[1] cowplot_1.2.0	tidyr_1.3.1	rethinking_2.42

[4] posterior_1.6.1	cmdstanr_0.9.0.9000 magrittr_2.0.3

[7] forcats_1.0.0	ggplot2_3.5.2	lubridate_1.9.4

[10] tibble_3.3.0	patchwork_1.3.1	dplyr_1.1.4

[13] readxl_1.4.5

loaded via a namespace (and not attached):

| [1] | tensorA_0.36.2.1 | generics_0.1.4 | shape_1.4.6.1 |
| --- | --- | --- | --- |
| [4] | lattice_0.22-7 | extrafontdb_1.0 | digest_0.6.37 |
| [7] | evaluate_1.0.4 | grid_4.5.1 | timechange_0.3.0 |
| [10] | RColorBrewer_1.1-3 | mvtnorm_1.3-3 | fastmap_1.2.0 |
| [13] | cellranger_1.1.0 | jsonlite_2.0.0 | processx_3.8.6 |
| [16] | backports_1.5.0 | ps_1.9.1 | purrr_1.0.4 |
| [19] | scales_1.4.0 | abind_1.4-8 | cli_3.6.5 |
| [22] | rlang_1.1.6 | withr_3.0.2 | yaml_2.3.10 |
| [25] | tools_4.5.1 | checkmate_2.3.2 | coda_0.19-4.1 |
| [28] | vctrs_0.6.5 | R6_2.6.1 | matrixStats_1.5.0 |
| [31] | lifecycle_1.0.4 | MASS_7.3-65 | pkgconfig_2.0.3 |
| [34] | pillar_1.11.0 | gtable_0.3.6 | loo_2.8.0 |
| [37] | glue_1.8.0 | xfun_0.52 | tidyselect_1.2.1 |
| [40] | rstudioapi_0.17.1 | knitr_1.50 | extrafont_0.19 |
| [43] | farver_2.1.2 | htmltools_0.5.8.1 | labeling_0.4.3 |
| [46] | rmarkdown_2.29 | Rttf2pt1_1.3.12 | compiler_4.5.1 |
| [49] | distributional_0.5.0 |  |  |
